# Design, synthesis, and evaluation of cyclic C7-bridged monocarbonyl curcumin analogs containing an *o*-methoxy phenyl group as potential agents against gastric cancer

**DOI:** 10.1080/14756366.2024.2314233

**Published:** 2024-02-22

**Authors:** Xin Gan, Yuna Wu, Min Zhu, Bo Liu, Miaomiao Kong, Zixuan Xi, Ke Li, Haibao Wang, Tiande Su, Jiali Yao, Fatehi Khushafah, Baozhu Yi, Jiabing Wang, Wulan Li, Jianzhang Wu

**Affiliations:** a The Second Affiliated Hospital and Yuying Children’s Hospital of the Wenzhou Medical University, Wenzhou, China; b Oujiang Laboratory (Zhejiang Lab for Regenerative Medicine, Vision and Brain Health), Wenzhou, China; c School of Pharmaceutical Sciences, Wenzhou Medical University, Wenzhou, China; d The Eye Hospital, School of Ophthalmology & Optometry, Wenzhou Medical University, Wenzhou, China; e The First Affiliated Hospital of Wenzhou Medical University, Wenzhou, China; f Municipal Hospital Affiliated to Taizhou University, Taizhou, China

**Keywords:** Cyclic C7 bridged monocarbonyl curcumin analogues, synthesis, anticancer activity, QSAR based on artificial intelligence, AKT/STAT3 inhibitor

## Abstract

The structure-activity relationship (SAR) between toxicity and the types of linking ketones of C7 bridged monocarbonyl curcumin analogs (MCAs) was not clear yet. In the pursuit of effective and less cytotoxic chemotherapeutics, we conducted a SAR analysis using various diketene skeletons of C7-bridged MCAs, synthesized cyclic C7-bridged MCAs containing the identified low-toxicity cyclopentanone scaffold and an *o*-methoxy phenyl group, and assessed their anti-gastric cancer activity and safety profile. Most compounds exhibited potent cytotoxic activities against gastric cancer cells. We developed a quantitative structure-activity relationship model (*R*^2^ > 0.82) by random Forest method, providing important information for optimizing structure. An optimized compound 2 exhibited *in vitro* and *in vivo* anti-gastric cancer activity partly through inhibiting the AKT and STAT3 pathways, and displayed a favorable *in vivo* safety profile. In summary, this paper provided a promising class of MCAs and a potential compound for the development of chemotherapeutic drugs.

## Introduction

**Figure 1.**
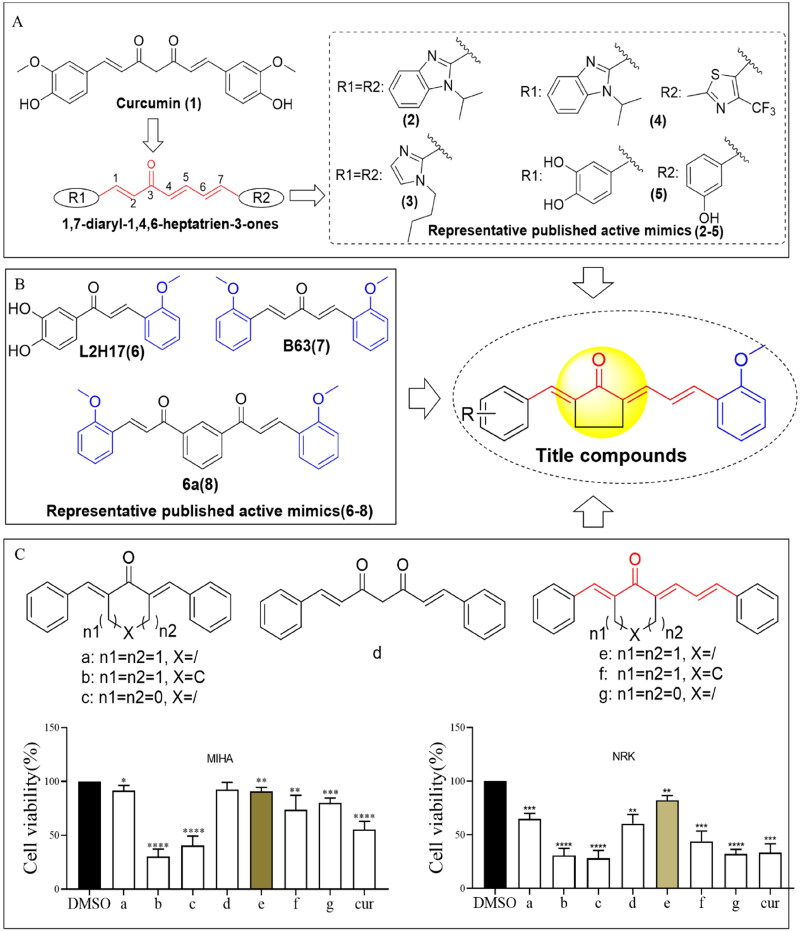
Design of 1,7-diphenylhepta-1,4,6-trien-3-one analogs comprising an *o*-methoxy phenyl group as anticancer agents. (A) Structure of curcumin and MCAs with anticancer activity featuring the 1,4,6-trien-3-one moiety. (B) Compounds exhibiting anticancer activity containing an *o*-methoxy phenyl ring. (C) Cytotoxicity of curcumin (cur) and its analog scaffold at 20 µM against MIHA and NRK cells. *p < 0.05, **p < 0.01, ***p < 0.001, ****p < 0.0001 vs. the DMSO group.

Gastric cancer, a highly prevalent and lethal malignancy, is commonly treated with chemotherapy. However, severe adverse effects hinder its effectiveness^1^. Natural products and their structural analogs provide potent cancer treatment with reduced toxicity^2^^,^^3^. Curcumin (Figure 1(A), 1), a major active ingredient from *Curcuma longa* of the *Zingiberaceae* family, is a staple in Asian diets, functioning as a yellow spice in curries^4^. Curcumin has demonstrated the ability to inhibit gastric cancer through multiple biological pathways in *in vitro* and *in vivo* studies^5–7^. Consequently, curcumin is regarded by many medical chemists as a potential lead compound in the development of novel drugs against gastric cancer.

Monocarbonyl curcumin analogs (MCAs) have been developed by substituting the 7-carbon β-diketone linker of curcumin with central linkers of various lengths, including 9-, 7-, and 5-carbon linkers. This approach enhances the development of curcumin-based anti-gastric cancer drugs with improved pharmaceutical properties^8^. Moreover, the length of the linker of MCAs impacts the anti-proliferative potency against cancer cells. Thus, the 9-carbon linker scaffold exhibits low potency, while the 5-carbon and 7-carbon linker scaffolds possess significantly stronger effects^9–11^. Meanwhile, the 7-atom linker (C7 bridge or 1,4,6-trien-3-one) is considered a promising bioisostere for curcumin due to its similarity in shape and size to curcumin’s enol-ketone tautomer. This suggests that MCAs with 7-carbon linkers could serve as potential scaffolds for the development of anti-cancer agents^10^. Currently, only three studies have reported on acyclic C7-bridged MCAs as potential anti-cancer agents. Some derivatives exhibited greater anticancer potency than curcumin *in vitro* (Figure 1(A), compounds (**2**)–(**5**))^10–12^. However, MCAs with a cyclic C7 bridge have not been reported, and the *in vivo* anticancer effects of C7-bridged MCAs remain unclear. Furthermore, the toxicity of MCAs with a 5-carbon linker is closely related to the types of central linking ketones^13–15^.

Whether MCAs with a cyclic C7 bridge offer improved safety and druggability remains unknown. Therefore, this study systematically explored the toxic effects of different linking ketones in C7-bridged MCAs on normal liver and kidney cells. By identifying a minimally toxic framework, a series of heterocyclic cyclopentanone 1,7-diphenylhepta-1,4,6-trien-3-one analogs containing o-methoxy phenyl and different phenyl moieties were designed. These analogs were created based on the principles of medicinal chemistry, and their antitumor activity was investigated *in vivo* and *in vitro*. Furthermore, their underlying mechanism was also explored.

## Results and discussion

### Design

We first designed and synthesised MCAs bearing a C7-bridged skeleton. These analogs incorporated linking diketene ketones derived from cyclopentanone, cyclohexanone, and acetone structural units. Specifically, compounds **e**, **g**, and **f** were obtained (Figure 1(C)). Subsequently, we evaluated their cytotoxicity against normal liver cell line MIHA and renal cell line NRK. As illustrated in Figure 1(C), compound **e** (featuring the cyclopentanone linking ketone) displayed lower cytotoxicity than compounds **f** and **g**.

To comprehensively investigate the impact of distinct types of linking ketones within the diketene skeletons on hepato-renal toxicity, we also synthesised MCAs with a C5-bridged skeleton, utilising cyclopentanone, cyclohexane, and acetone structural units. The cyclopentanone-derived compound **a** had the lowest cytotoxicity among all (Figure 1(C)). Therefore, from the perspective of safety, we hypothesised that MCAs derived from the cyclopentanone structural unit might provide the most promising scaffold. For further details on synthetic procedures, colouration, and melting points, liquid chromatography–mass spectrometry (LC–MS), and proton nuclear magnetic resonance (^1^H NMR) spectra of compounds **a–g** are presented in the Supplementary material.

Accumulating evidence confirms that the activity of MCAs is influenced by the substituents on the benzene ring. Thus, compounds containing an *o*-methoxy phenyl group have shown promise as potential anticancer agents (Figure 1(B)). For example, L2H17 containing an *o*-methoxy phenyl moiety, exhibited significant *in vitro* and *in vivo* antiproliferative effects against colon cancer^16^. Compound **B63**, a 1,5-bis(2- methoxyphenyl)penta-1,4-dien-3-one, induced apoptosis and paraptosis-like cell death in lung and gastric cancers, respectively (Figure 1(B))^17^^,^^18^. Additionally, the curcumin analogue **6a** which incorporated an *o*-methoxy phenyl group also exhibited superior cytotoxicity against A431 and DLD1 cell lines^19^. Moreover, MCAs featuring an acyclic C7 bridge containing two different heteroaromatic rings exhibited greater potency against prostate cancer cell lines than the corresponding ones with two identical heteroaromatic rings. This suggests that C7-bridged MCAs with two different terminal heteroaromatic rings could serve as a more favourable scaffold for anti-cancer agent development^11^. Therefore, herein we designed and synthesised a series of heterocyclic cyclopentanone 1,7-diphenylhepta-1,4,6-trien-3-one analogs containing an *o*-methoxy phenyl group and different phenyl moieties (Figure 1).

### Chemical synthesis

**Scheme 1.**
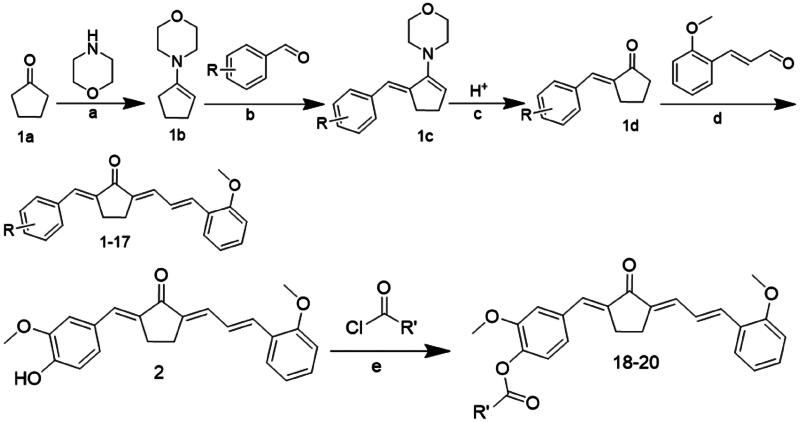
The general route for asymmetric synthesis

The synthesis details and chemical structures of the compounds are outlined in Scheme 1, Tables 1 and 2. Generally, a four-step reaction process was employed to prepare compounds **1–17**. The initial step involved activating cyclopentanone **1a** with morpholine to form enamine **1b**. Subsequently, an ethanolic solution of **1b** was reacted with different substitutional aromatic aldehydes at 78 °C, yielding compound **1c**. The pH was adjusted to acidic to obtain the key intermediate **1d**. These solutions were condensed with *o*-methoxycinnamaldehyde or other reagents to synthesise compounds **1**–**17**. For the synthesis of compounds **18**–**20**, compound **2** was reacted with various acyl chloride. All compounds were efficiently obtained and purified using column chromatography or re-crystallization, resulting in good yields. The products were characterised by analysis and comparison of their LC–MS and ^1^H NMR spectral data. Comprehensive information, including the colour, melting point, LC–MS, and ^1^H NMR spectra of the compounds, can be found in the chemical section.

**Table 1.** Chemical structures of compounds **1–17**.

Comp.	R	Comp.	R
**1**	4-N(CH_3_)_2_	**10**	2-OCH_3_
**2**	3-OCH_3_, 4-OH	**11**	4-N(CH_2_CH_2_)O
**3**	4-OCH_3_	**12**	2,3-OCH_3_
**4**	2-OH	**13**	3.4-OH
**5**	4-OH	**14**	2,5-OCH_3_
**6**	3-OH	**15**	4-N(CH_2_CH_2_Cl)_2_
**7**	2,4,6-OCH_3_	**16**	3,4,5-OCH_3_
**8**	3-OH, 4-OCH_3_	**17**	2-OH, 3-OCH_3_
**9**	2,4-OCH_3_		

**Table 2.** Chemical structures of compounds **18–20**.

Comp.	R′
**18**	–CH = CH_2_
**19**	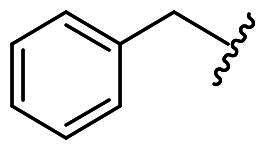
**20**	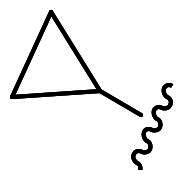

Reagents and conditions: (a) p-TSA, cyclohexane, reflux, 90 °C; (b) EtOH, reflux, 78 °C; (c) 10% HCl solution, room temperature; (d) EtOH, 40% NaOH or HCl gas, room temperature; (e) anhydrous DMSO, Et3N, room temperature.

### Inhibitory effects on cell viability and evaluation of quantitative structure–activity relationship (QSAR)

The anti-viability activities of compounds (**1–17**) were assessed against human gastric cancer cell lines SGC-7901 and BGC-823 using 3-(4,5- dimethylthiazol-2-yl)-2,5-diphenyl tetrazolium bromide (MTT) assay. For comparison, piperlongumine (PL), curcumin (cur), EF24, and B63 were used as positive controls.

**Figure 2.**
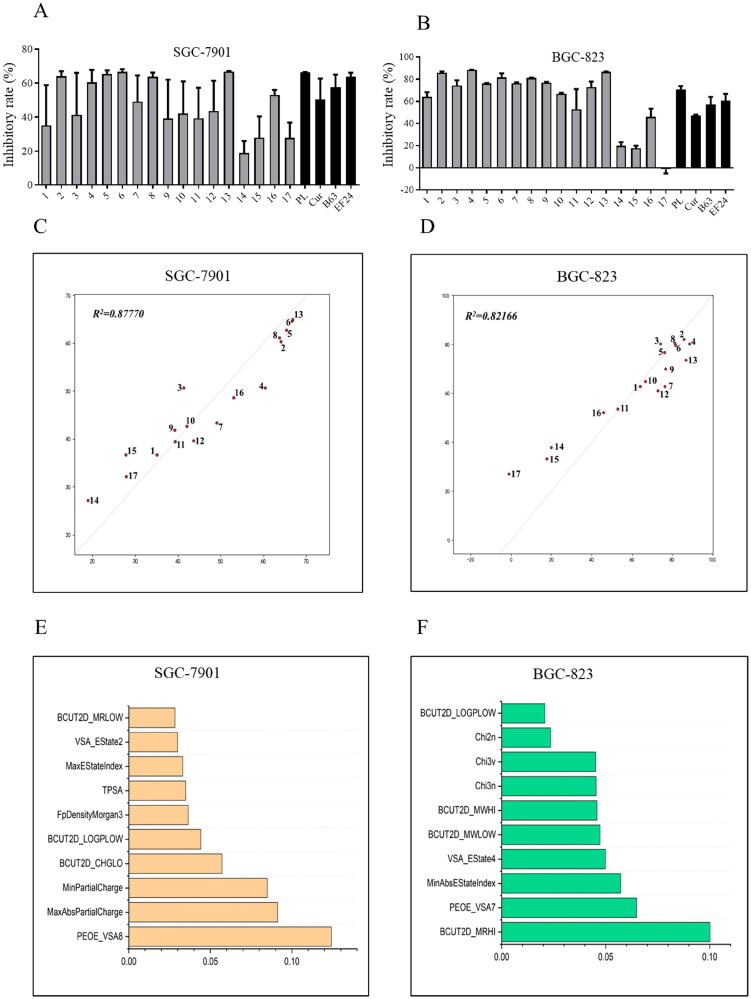
Anti-viability activities and quantitative structure–activity relationship (QSAR) analysis of 1,7-diphenyl hepta-1,4,6-trien-3-one analogs containing an *o*-methoxy phenyl group. Compounds inhibited the growth of cancer cells SGC-7901 (A) and BGC-823 (B). Cancer cells were treated with compounds (10 μM) for 72 h. The optical density values were measured using the MTT assay, and the resulting inhibitory rates were calculated. The QSAR model and the top 10 important features of compounds active against SGC-7901 (C and E) and BGC-823 (D and F) cells.

As shown in Figure 2(A) and (B), the preliminary screening revealed that most compounds exhibited significantly improved anticancer activity against the tested human cancer cells when compared to positive controls. Compounds **14** (2,5-methoxy-), **15** (4-[bis-(2-chloroethyl)amino-]), and **17** (2-hydroxy-3-methoxy-) demonstrated decreased cytotoxicity against BGC-823 and SGC-7901 cells, although compound **15** has N-bis-beta-chloroethyl moiety, which is the main active structure of the antitumor drug chlormethine.

QSAR analysis has been widely used to predict the biological effects of chemicals^20–22^. The Random Forest (RF) method used in this study is an effective machine learning algorithm for building QSAR models, particularly suitable for small sample sets and high-dimensional problems^20^. Hence in this study, we constructed a QSAR model using the RF method to investigate the structure–activity relationship (SAR) of the 1,7-diphenyl hepta-1,4,6-trien-3-one analogs containing an o-methoxy phenyl group. The results revealed that the *R*^2^ values of the prediction model for SGC-7091 cells and BGC-823 cells were 0.87770 and 0.82166, respectively, indicating the robustness and reliability of the prediction model. Furthermore, we explored the two sets of models by assessing the importance of their features. For BGC-823 cells, most of the top 10 important features are BCUT2D and Chi series features describing the topological nature of the compound. Therefore, the anti-tumour activity of the compound on BGC-823 cells is closely related to the topological structure of the compound itself, indicating that the scaffold of compound may contribute significantly to the antitumor activity. For SGC-7901 cells, most of the top ten important features are log*P* and charge property series features. Therefore, we calculated the log*P* and charge profiles of the compounds separately and mapped them to the atoms. The results showed that the more active compounds have some electron-withdrawing properties at the 3 and 4 positions of the benzene ring, as well as some hydrophilicity. In summary, our model demonstrates a discernible linear relationship between the compound structure and activity within this series of compounds and can predict their activity to a certain extent. Thus, it may provide valuable clues for the development of 1,7-diphenyl hepta-1,4,6-trien-3-one derivatives with potential applications in the treatment of various types of cancer.

To further evaluate the cytotoxic activities of the compounds, we assessed the half inhibitory concentration (IC_50_) of the compounds with potent antitumor activity (**2**, **4**, **5**, **6,** and **13**) based on the results of the MTT assay (Table 3). Compared to positive controls, the tested compounds exhibited significant anti-viability effects against gastric cancer cells, with most of them displaying IC_50_ values below 10 μM. Remarkably, compound **2** exhibited exceptional potency, with an IC_50_ value of 1.39 ± 1.04 μM against SGC-7901 cells, being selected for subsequent development.

**Table 3.** The half inhibitory concentration (IC_50_, µM) of tested compounds against SGC-7901 and BGC-823 cancer cell lines.

Compound	IC_50_ (µM)	
	SGC-7901	BGC-823
**2**	1.39 ± 1.04	2.92 ± 1.52
**4**	9.69 ± 2.15	13.40 ± 3.02
**5**	7.35 ± 4.90	6.07 ± 3.38
**6**	6.35 ± 1.59	6.53 ± 1.50
**13**	2.08 ± 1.27	5.62 ± 4.58
**18**	58.12 ± 1.95	17.26 ± 1.08
**19**	28.58 ± 1.95	30.60 ± 3.35
**20**	45.79 ± 1.95	19.60 ± 4.67
**B63**	14.13 ± 0.52	10.27 ± 0.39
**PL**	12.58 ± 2.89	12.25 ± 2.51
**Cur**	17.38 ± 0.33	16.84 ± 0.89
**EF24**	3.95 ± 2.70	4.54 ± 2.59

Cur, curcumin; PL, piperlongumine.

The presence of 3-methoxy-4-hydroxyphenyl functionality contributes to enhancing the anticancer properties of the compound^23–26^. To gain deeper insights into the anticancer potential of this functional group, we acetylated the 3-methoxy-4-hydroxyphenyl moiety of compound **2**, resulting in the creation of compounds **18**–**20**. Compared to compound **2**, compounds **18**–**20** displayed inactivity against SGC-7901 and BGC-823 cells, with IC_50_ values ranging from 17.26 ± 1.08 to 58.12 ± 1.95 μM. This observation underscores the value of incorporating the 3-methoxy-4-hydroxyphenyl moiety on the benzene ring to enhance the inhibition efficacy.

### Compound 2 inhibits the proliferation and migration of SGC-7901 cancer cells

**Figure 3.**
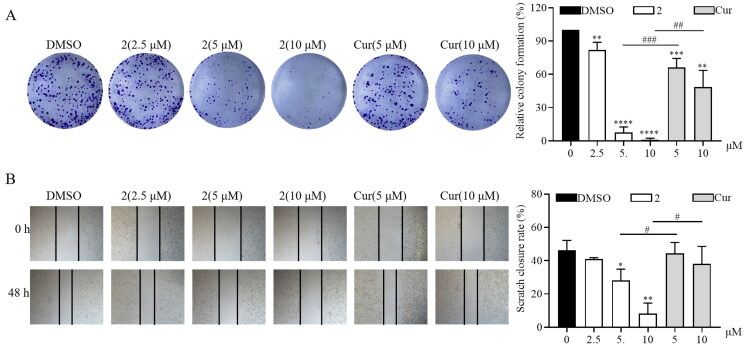
Compound **2** inhibits the proliferation and migration of SGC-7901 cancer cells. (A) The SGC-7901 cells treated with different concentrations of compound **2** (2.5, 5, and 10 µM) and curcumin (cur) (5 and 10 µM). Following a 12-h treatment period, the medium was replaced with a normal medium, and the cells were incubated until visible colonies formed. Finally, the colonies were stained with crystal violet staining solution and photographed. (B) The effect of compound **2** on the migration of SGC-7901 cells. The images were captured using phase contrast microscopy before (0 h) and after 48 h of treatment with different concentrations of compound **2** (2.5, 5, and 10 µM) and cur (5 and 10 µM). (*p < 0.05, **p < 0.01, ***p < 0.001, ****p < 0.0001 vs. DMSO group; #*p* < 0.05, ##*p* < 0.01, ###*p* < 0.001).

To further investigate the anticancer activity of compound **2**, its antiproliferative and anti-migration activities were measured. Curcumin was used as the positive control. The colony formation assay was performed to examine the antiproliferative effect of compound **2**. As illustrated in Figure 3(A), treatment with compound **2** decreased the proliferation of SGC-7901 cancer cells in a dose-dependent manner. Furthermore, its inhibitory activity was higher than that of curcumin at 10 μM. Moreover, compound **2** at various concentrations (2.5, 5, and 10 μM) determined a significantly lower cell migration than curcumin (5, 10 μM), as revealed by cell scratch wound-healing assay (Figure 3(B)). Collectively, these results demonstrate that compound **2** can efficaciously inhibit the proliferation and migration of gastric cancer cells.

### Impact of compound 2 on apoptosis and cell cycle arrest in SGC-7901 cancer cells

**Figure 4.**
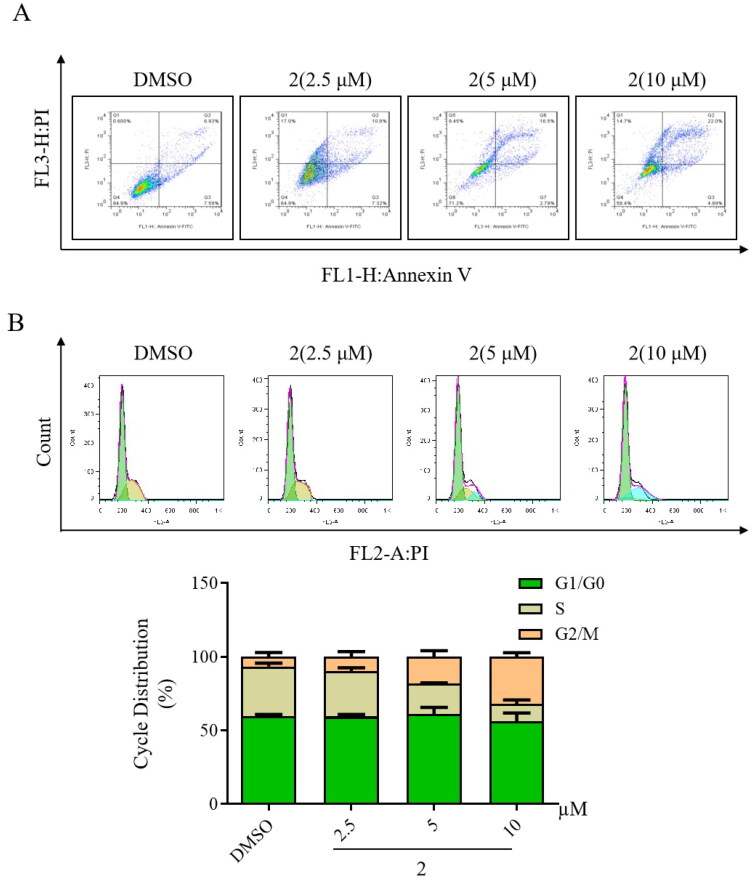
Impact of compound **2** on apoptosis and cell cycle arrest in SGC-7901 cancer cells. (A) Apoptosis in SGC-7901 cells was assessed following an 18-h treatment with compound **2** at different concentrations (2.5, 5, and 10 µM). Cells were stained with annexin V- fluorescein isothiocyanate and propidium iodide and quantified using a flow cytometer. (B) SGC-7901 cells were incubated with compound **2** (2.5, 5, and 10 µM) for 18 h. Subsequently, they were fixed and stained, and DNA content was assessed. The distribution and percentage of cells in the G1/G0, S, and G2 phases of the cell cycle are indicated.

The effect of compound **2** on apoptosis in gastric cancer SGC-7901 cells was examined using flow cytometry. As illustrated in Figure 4(A), treatment with compound **2** determined a dose-dependent increase in apoptosis in SGC-7901 cancer cell lines. Furthermore, the modulation of the cell cycle by compound **2** was investigated in gastric cancer SGC-7901 cells through flow cytometry. Compared to control, treatment with compound **2** resulted in a dose-dependent increase in the number of cells in the G2/M phase (Figure 4(B)). These findings strongly suggest that the anticancer mechanism of compound **2** may be associated with both inducing cell apoptosis and triggering cell cycle arrest.

### Compound 2 inhibited the proliferation of SGC-7901 by blocking the signal transducer and activator of transcription 3 (STAT3)/protein kinase B (AKT) signalling pathway

**Figure 5.**
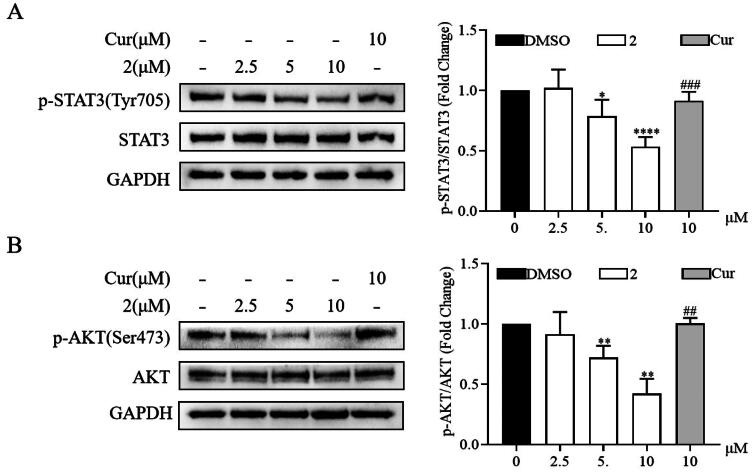
Effects of compound **2** on STAT3/AKT signalling pathway in SGC-7901 cancer cells. The protein expression levels of p-STAT3, STAT3 (A) and p-AKT, AKT (B) in SGC-7901 cell lines after treatment with compound **2** (2.5, 5, and 10 µM) and curcumin (10 µM). *p < 0.05, **p < 0.01, ****p < 0.0001 vs. the control group. ##*p* < 0.01, ###*p* < 0.001 vs. compound **2** (10 µM)-treated group.

Continuous activation of STAT3 and AKT promotes the malignant progression of various tumour cells, including gastric cancer cells^27^^,^^28^. Thus, the mechanism underlying the anticancer effect of compound **2** was further investigated through Western blot. Compound **2** reduced the phosphorylation of STAT3 and AKT in a dose-dependent manner (Figure 5(A) and (B)). Furthermore, compound **2** exhibited a significant reduction in the phosphorylation of STAT3 and AKT compared to curcumin at equivalent concentrations. These results underscore the involvement of the STAT3 and AKT signalling pathways in the progression of SGC-7901 and indicate that the anticancer effect of compound **2** is related to the inhibition of these pathways.

### Compound 2 significantly reduced tumour growth in an animal model of gastric cancer

**Figure 6.**
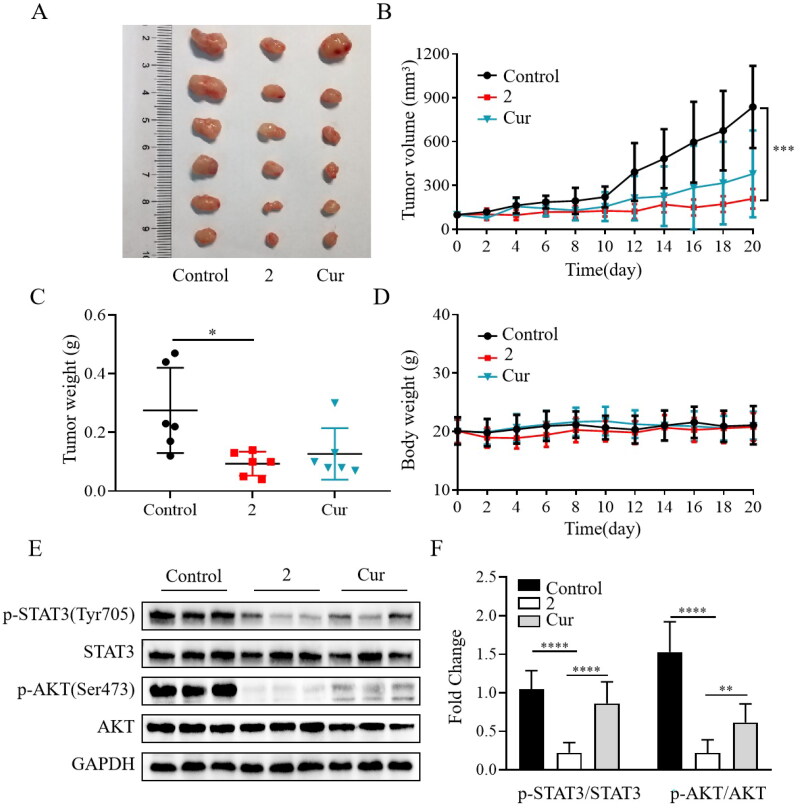
Compound **2** reduced tumour growth in the SGC-7901 mouse xenograft model. (A) Gross morphology of tumours excised from each group of mice 20 days after treatment. (B) Tumour volume assessment illustrating the inhibitory effect of compound **2** (15 mg/kg) on tumour growth compared to curcumin (cur) (20 mg/kg) or vehicle (control). (C) The average weights of xenograft tumours at the end of treatment. (D) Body weight changes observed during the 20-day dosing period. (E–F) The protein expression levels of p-STAT3, STAT3, p-AKT, and AKT in tumour tissues. *p < 0.05, **p < 0.01, ***p < 0.001, ****p < 0.0001.

After identifying compound **2** as a potent anticancer agent in cellular assays, the *in vivo* efficacy assessment was carried out using the SGC-7901 mouse xenograft model. The mice were implanted with SGC-7901 cells to develop tumours and subsequently treated daily with compound **2**, curcumin, or vehicle by intraperitoneal injection before the mice were sacrificed. As shown in Figure 6(A)–(C), compound **2** at 15 mg/kg significantly inhibited the tumour growth in terms of both volume and weight. Furthermore, the *in vivo* anticancer activity of compound **2** was superior to that of curcumin (20 mg/kg). Compound **2** was well-tolerated over the 20 days of treatment, as evidenced by the absence of noticeable signs of toxicity or significant weight loss (Figure 6(D)). These findings indicate that compound **2** had a good safety profile in animal models. Compared to the vehicle or curcumin, compound **2** significantly reduced the phosphorylation of STAT3 and AKT (Figure 6(E) and (F)). Overall, compound **2** demonstrated a remarkable reduction in tumour growth and exhibited a favourable safety profile in our study. Our findings suggest that one of the potential anticancer mechanisms of compound **2** involves the inhibition of the STAT3/AKT signalling pathway.

### Safety evaluation of compound 2 in vivo

**Figure 7.**
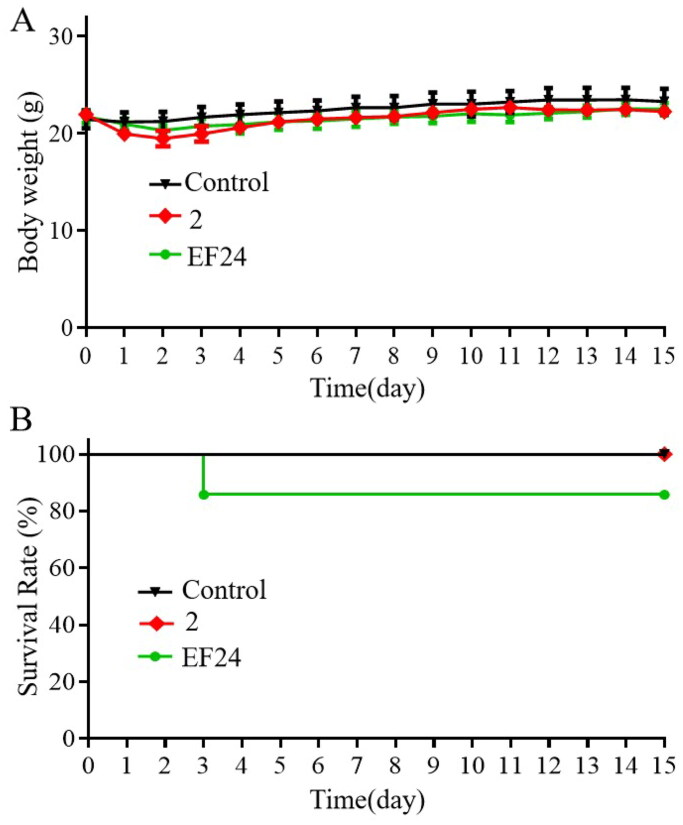
Compound **2** possesses a favourable *in vivo* safety profile. Compound **2** and EF24 (600 mg/kg) were administered to the mice on day 1 (A) Variation of body weight, and (B) mortality rate throughout the 15-day observation period.

While the SGC-7901 xenograft tumour experiment indicated the safety of compound **2** *in vivo*, an acute toxicity assessment was conducted to further characterise its safety profile. EF24^29^, a candidate compound with a known safety profile and excellent anticancer activity, was used as a positive control. The treatments with compound **2** and EF24 (600 mg/kg) did not result in significant weight loss compared to the control group (Figure 7(A)). Moreover, all mice treated with compound **2** survived throughout the 15-day observation period. However, EF24-treated mice experienced a 17% mortality rate, indicating that compound **2** might have lower toxicity than EF24 (Figure 7(B)). Overall, these results indicate that compound **2** possesses a favourable *in vivo* safety profile.

## Discussion and conclusion

Gastric cancer ranks as the fifth most prevalent malignancy globally and the third leading cause of cancer-related mortality, accounting for approximately 723,000 annual deaths^1^. Most patients are diagnosed in an advanced stage of the disease when surgical intervention is no longer a viable therapeutic option. As a result, pharmacological treatment assumes primary importance. The advances in the molecular biology of gastric cancer lead to the emergence of targeted therapy as a focal point in clinical practice. This approach has notably extended the overall survival of individuals with advanced gastric cancer. Recently identified targets of gastric cancer comprise human epidermal growth factor receptor 2, programmed cell death protein 1, programmed cell death ligand 1, and vascular endothelial growth factor 2. Despite this, only a few effective targeted agents are available for the treatment of patients with advanced gastric cancer^30^. Additionally, not all driver mutations in advanced gastric cancer have been successfully identified and paired with effective targeted drugs. As a result, the benefits of targeted therapy are somewhat limited, making classical cytotoxic drugs the primary choice for patients who do not respond to targeted therapies^31^. At the same time, the emergence of severe adverse effects and complications arising from chemotherapy represents an additional clinical challenge^32^. Given these circumstances, there is a pressing need for the development of new drugs to significantly transform the current landscape of gastric cancer.

Natural products have played an important role in the discovery of small-molecule anti-tumour drugs. Increasing attention is being directed towards the biological functions of curcumin, which has been certified by the Food and Drug Administration (FDA) as having a high safety profile in humans. Structural modifications applied to curcumin have emerged as a valuable approach for discovering analogs with enhanced anticancer potential. Notably, modifying the lengths of the central linker of curcumin derivatives has shown promise in enhancing their anticancer properties. Particularly, the 1,4,6-trien-3-one motif, retaining the 7-atom linker (C7 bridge), has been identified as a favourable bioisostere of the keto-enol central spacer in curcumin^10^. Up to now, all reported MCAs are derived from 1,4,6-trien-3-one, comprising acetone as the central linker. These optimised compounds have exhibited significantly improved antiproliferative potency compared to curcumin (Figure 1(A)). However, one study^12^ reported that most of these analogs are toxic against Vero cells, their toxicity surpassing that of curcumin.

To mitigate the cytotoxicity of MCAs containing the 1,4,6-trien-3-one motif, following a comprehensive screening of various linking ketones, we have developed MCAs containing a cyclopentanone-derived central linker (Figure 1(C)). Our study includes preliminary SAR evaluating the association between the anticancer activity or toxicity of heterocyclic cyclopentanone-derived MCAs containing the 1,4,6-trien-3-one skeleton and various types of central linker ketones. However, further systematic studies are needed to gain a deeper understanding of these relationships.

To investigate the effects of the *o*-methoxy phenyl ring and cyclopentanone skeleton on the anticancer activity of MCAs possessing a 7-atom linker, our current study focused on the synthesis and evaluation of a series of cyclic C7-bridged MCAs. These new analogs contain an *o*-methoxy phenyl group and a cyclopentanone skeleton. Their synthesis was guided by a combination of established principles, and their potential chemotherapeutic activity against gastric cancer was evaluated.

During the initial screening, most compounds displayed a significant improvement in their anticancer activity against SGC-7901 and BGC-823 cells compared to positive controls. These results indicate that the 1,7-diphenylhepta-1,4,6-trien-3-one structure in conjunction with the *o*-methoxy phenyl group could serve as a potential scaffold for the development of novel therapeutic agents effective against gastric cancer.

The random forest method has strong advantages in establishing QSAR models for small sample sets^20^. In this study, although the sample set was only 17 compounds, the models in both cells showed excellent regression ability, which the *R*^2^ values of the prediction model for SGC-7091 cells and BGC-823 cells were 0.87770 and 0.82166, respectively. Moreover, using QSAR analysis, we confirmed a correlation between the inhibition potency of these synthetic compounds and their log*P*, charge properties, and molecular topology.

Particularly, one optimal compound was identified and selected for further testing – compound **2,** which exhibited substantially stronger cytotoxic activity than the other analogs. Furthermore, diminishing the 3-methoxy- 4-hydroxyphenyl moiety of compound **2** by acylation resulted in a noticeable reduction in cell viability, indicating the importance of the 3-methoxy-4-hydroxyphenyl skeleton for the inhibition potency. These results^33^^,^^34^ are consistent with those of previously published literature on active compounds derived from this structure. In summary, the *o*-methoxy phenyl, 3-methoxy-4-hydroxyphenyl, and 1,4,6-trien-3-one groups of MCAs may be critical for their anticancer effects.

Generally, cancer cells are more adaptable to the microenvironment and tend to form colonies and migrate^35^. Compound **2** reduced the number of cell colonies and suppressed cell migration in a dose-dependent manner. Apoptosis, a strictly controlled type of programmed cell death, can be initiated through internal pathways^36^^,^^37^. In this study, compound **2** effectively induced gastric cancer cell apoptosis, in a dose-dependent manner by triggering cell cycle arrest in the G2/M phase.

Additionally, we revealed the potential involvement of the AKT and STAT3 pathways in the anti-proliferative effect exhibited by compound **2** against SGC-7901 cells *in vitro* and *in vivo*. To date, no reports exist on the *in vivo* antiproliferative potency and toxicity of MCAs containing the 1,4,6-trien-3-one skeleton. In this study, the selected compound **2** reduced tumour growth in the SGC-7901 mouse xenograft model. Furthermore, it also exhibited a good *in vivo* safety profile. In conclusion, this work revealed that the cyclic C7-bridged MCAs derived from cyclopentanone and comprising an *o*-methoxy phenyl ring may be potent therapeutic alternatives with enhanced anticancer activities. Furthermore, compound **2** emerged as a promising candidate for the development of chemotherapeutic drugs. However, the underlying molecular mechanism needs to be further clarified.

## Experimental

### Chemical procedures

Unless otherwise specified, all reagents were obtained from the commercial suppliers Sigma-Aldrich and Aladdin and were used without further purification. The progression of the reaction processes was monitored using thin-layer chromatography (TLC) on silica gel GF254 plates. Chromatograms were developed on silica gel (200–300 mesh size) and visualised under UV light at 254 and 365 nm. Melting points were determined using open capillary tubes on a Fisher–Johns melting apparatus. Mass spectra were determined on an Agilent 1100 LC–MS (Agilent, Palo Alto, CA, USA). ^1^H spectral data were collected using a 600 or 400 MHz spectrometer (Bruker Corporation, Switzerland) employing tetramethylsilane as an internal standard. Chemical shifts (*d*) are expressed in parts per million (ppm) relative to the residual solvent signal. Coupling constant values (*J*) are given in Hertz (Hz) and correspond to apparent multiplicities. Splitting patterns were designated as follows: s = singlet, d = doublet, t = triplet, dd = doublet of doublets, m = multiplet.

**(E)-2-((E)-4-(dimethylamino)benzylidene)-5-((E)-3-(2-methoxyphenyl)allylidene)cyclopentan-1-one (1):** Red powder, 58% yield, mp 220.6–222.5 °C. ^1^H NMR (600 MHz, CDCl_3_), *δ*: 7.560 (d, *J* = 6.6 Hz, 1H, H-ɤ), 7.502 (s, 1H, Ar–H^6^), 7.526 (d, *J* = 8.4 Hz, 2H, Ar–H^2′^, Ar–H^6′^), 7.335 (d, *J* = 15.6 Hz, 2H, Ar–H^4^, H-*δ*), 7.287 (t, *J* = 4.2 Hz, H-α), 7.056–7.011 (m, 1H, Ar–H^3^), 6.958 (t, *J* = 7.2 Hz, 1H, Ar–H^5^), 6.898 (d, 1H, H-β), 6.761 (s, 2H, Ar–H^3′^, Ar–H^5′^), 3.889 (s, 3H, 2-OCH_3_), 3.045 (s, 8H, 4′-N(CH_3_)_2_, CH_2_), 2.914 (t, *J* = 4.8 Hz, 2H, CH_2_). LC–MS *m*/*z*: 360.02 (M + H)^+^, calcd for C_24_H_25_NO_2_: 359.19.

**(E)-2-((E)-4-hydroxy-3-methoxybenzylidene)-5-((E)-3-(2-methoxyphenyl)allylidene)cyclopentan-1-one (2):** Brown-yellow power, 89.4% yield, mp 226.0–228.1 °C. ^1^H NMR (400 MHz, DMSO), *δ*: 9.681(s, 1H, 4-OH), 7.701 (d, *J* = 7.2 Hz, 1H, H-*δ*), 7.344–7.301 (m, 3H, Ar–H^4^, Ar–H^6^, Ar–H^6′^), 7.188–7.093(m, 3H, Ar–H^3^, Ar–H^5^, H-ɤ)), 7.046 (d, *J* = 8.4 Hz, 1H, Ar–H^1′^), 7.222 (s, 1H, H-α), 6.973(t, *J* = 7.6, 1H, AH^5′^), 6.873 (d, *J* = 8.0 Hz, 1H, β-H), 3.849 (s, 3H, 2′-OCH_3_), 3.825 (s, 3H, 3-OCH_3_), 3.015–2.910 (m, 4H, CH_2_CH_2_). LC–MS *m*/*z*: 363.15 (M + H)^+^, calcd for C_23_H_22_O_3_: 362.15.

**(E)-2-((E)-4-methoxybenzylidene)-5-((E)-3-(2-methoxyphenyl)allylidene)cyclopentan-1-one (3):** Yellow powder, 56.8% yield, mp 176.4–177.1 °C. ^1^H NMR (600 MHz, CDCl_3_), *δ*: 7.716 (d, *J* = 7.2 Hz, 1H, H-ɤ), 7.639 (d, *J* = 8.4 Hz, 2H, Ar–H^2′^, Ar–H^6′^), 7.355–7.316 (m, 3H, Ar–H^4^, Ar–H^6^, H-δ), 7.193–7.148 (m, 2H, H-α, Ar–H^3^), 7.071–7.046 (m, 3H, Ar–H^5^, Ar–H^3^, Ar–H^5′^), 6.990 (t, *J* = 15.0 Hz, 1H, H-β), 3.866 (s, 3H, 2-OCH_3_), 3.823 (s, 3H, 4′-OCH_3_), 3.008–2.917 (m, 4H, CH_2_CH_2_). LC–MS *m*/*z*: 347.20 (M + H)^+^, calcd for C_23_H_22_O_3_: 346.16.

**(E)-2-((E)-2-hydroxybenzylidene)-5-((E)-3-(2-methoxyphenyl)allylidene)cyclopentan-1-one (4):** Brown powder, 75% yield, mp 219.5–221.3 °C. ^1^H NMR (600 MHz, DMSO), *δ*: 10.134 (s, 1H, 2-OH), 7.746 (d, *J* = 3.0 Hz, 1H, H-ɤ), 7.725–7.711 (m, 1H, Ar–H^6^), 7.543 (d, *J* = 7.8 Hz, 1H, H-δ), 7.355–7.322 (m, 2H, Ar–H^4^, Ar–H^6′^), 7.259–7.231 (m, 1H, H-α), 7.198–7.196 (m, 2H, Ar–H^3^, Ar–H^4′^), 7.064 (d, *J* = 8.4 Hz, 1H, Ar–H^5^), 6.989 (t, *J* = 15.0 Hz, 1H, Ar–H^5′^), 6.939 (d, *J* = 7.8 Hz, 1H, Ar–H^3′^), 6.897 (t, *J* = 15.0 Hz, 1H, H-β), 3.867 (s, 3H, 2-OCH_3_), 2.992–2.902 (m, 4H, CH_2_CH_2_). LC–MS *m*/*z*: 333.17 (M + H)^+^, calcd for C_22_H_20_O_3_: 332.14.

**(E)-2-((E)-4-hydroxybenzylidene)-5-((E)-3-(2-methoxyphenyl)allylidene)cyclopentan-1-one (5):** Red powder, 56% yield, mp 213.8–214.3 °C. ^1^H NMR (600 MHz, DMSO), *δ*: 10.083 (s, 1H, 4-OH), 7.721–7.706 (m, 1H, H-ɤ), 7.535 (d, *J* = 8.4 Hz, 2H, Ar–H^4^, Ar–H^6^), 7.352–7.323 (m, 2H, Ar–H^2′^, Ar–H^5′^), 7.302 (d, *J* = 3.6 Hz, 1H, H-δ), 7.189–7.112 (m, 2H, H-α, Ar–H^3^), 7.061 (d, *J* = 8.4 Hz, 1H, Ar–H^5^), 6.988 (t, *J* = 15.0 Hz, 1H, H-β), 6.880 (d, *J* = 8.4 Hz, 2H, Ar–H^3′^, Ar–H^5′^), 3.854 (s, 3H, 2-OCH_3_), 2.986–2.972 (m, 2H, CH_2_), 2.935–2.910 (m, 2H, CH_2_). LC–MS *m*/*z*: 333.17 (M + H)^+^, calcd for C_22_H_20_O_3_: 332.14.

**(E)-2-((E)-3-hydroxybenzylidene)-5-((E)-3-(2-methoxyphenyl)allylidene)cyclopentan-1-one (6):** Brown powder, 58% yield, mp 229.2–230.4 °C. ^1^H NMR (600 MHz, CDCl_3_), *δ*: 9.611 (s, 1H, 3′-OH), 7.701 (dd, *J* = 1.8 Hz, 6 Hz, 1H, H-ɤ), 7.340–7.311 (m, 2H, Ar–H^6^, Ar–H^4^), 7.268 (d, 1H, H-δ), 7.248–7.234 (m, 1H, Ar–H^5′^), 7.165–7.147 (m, 2H, H-α, Ar–H^6′^), 7.074 (d, *J* = 7.8 Hz, 1H, Ar–H^3^), 7.053–7.041 (m, 2H, Ar–H^5^, Ar–H^4′^), 6.971 (t, *J* = 7.2 Hz, 1H, Ar–H^2′^), 6.814 (dd, J = 1.8 Hz, 6 Hz, 1H, H-β), 3.848 (s, 3H, 2-OCH_3_), 3.002–2.906 (m, 4H, CH_2_). LC–MS *m*/*z*: 333.17 (M + H)^+^, calcd for C_22_H_20_O_3_: 332.14.

**(E)-2-((E)-3-(2-methoxyphenyl)allylidene)-5-((E)-2,4,6-trimethoxybenzylidene)cyclopentan-1-one (7):** Yellow powder, 58% yield, mp 105.7–106.3 °C. ^1^H-NMR (600 MHz, CDCl_3_), *δ*: 7.590 (t, *J* = 2.4 Hz, 1H, H-ɤ), 7.552 (d, *J* = 7.2 Hz, 1H, Ar–H^6^), 7.328 (d, *J* = 15Hz, 1H, H-δ), 7.300–7.274 (m, 2H, Ar–H_4_, H-α), 7.041–6.995 (m, 1H, Ar–H^3^), 6.952 (t, *J* = 7.8 Hz, 1H, Ar–H^5^), 6.896 (d, *J* = 8.4 Hz, 1H, H-β), 6.150 (s, 2H, Ar–H^3′^, Ar–H^5′^), 3.888 (s, 3H, 2-OCH_3_), 3.855 (s, 3H, 4′-OCH_3_), 3.833 (s, 6H, 2-OCH_3_, 6-OCH_3_), 2.797–2.772 (m, 2H, CH_2_), 2.666–2.636 (m, 2H, CH_2_). LC–MS *m*/*z*: 407.26 (M + H)^+^, calcd for C_25_H_26_O_5_: 406.18.

**(E)-2-((E)-3-hydroxy-4-methoxybenzylidene)-5-((E)-3-(2-methoxyphenyl)allylidene)cyclopentan-1-one (8):** Red powder, 58% yield, mp 180.1–181.8 °C. ^1^H NMR (600 MHz, CDCl_3_), *δ*: 7.562 (dd, *J* = 1.2 Hz, 6 Hz, 1H, H-ɤ), 7.446 (t, *J* = 5.4 Hz, 1H, Ar–H^6^), 7.361 (d, *J* = 15.6 Hz, 2H, Ar–H^4^, H-δ), 7.302–7.274 (m, 1H, H-α), 7.221 (d, *J* = 2.4 Hz, 1H, Ar–H^6′^), 7.124 (dd, *J* = 1.8 Hz, 6.6 Hz, 1H, Ar–H^2′^), 7.055–7.010 (m, 1H, Ar–H^3^), 6.962 (t, *J* = 15Hz, 1H, H-β), 6.904 (d, *J* = 8.4 Hz, 2H, Ar–H^3′^, Ar–H^5^), 3.943 (s, 3H, 2-OCH_3_), 3.893 (s, 3H, 4′-OCH_3_), 3.055–3.038 (m, 2H, CH_2_), 2.940 (t, *J* = 7.2 Hz, 2H, CH_2_). LC–MS *m*/*z*: 363.21 (M + H)^+^, calcd for C_23_H_22_O_4_: 362.15.

**(E)-2-((E)-2,4-dimethoxybenzylidene)-5-((E)-3-(2-methoxyphenyl)allylidene)cyclopentan-1-one (9):** Yellow powder, 57% yield, mp 158.2–160.2 °C. ^1^H NMR (600 MHz, CDCl_3_), *δ*: 7.944 (s, 1H, Ar–H^6′^), 7.583 (d, *J* = 7.8 Hz, 1H, H-ɤ), 7.529 (d, *J* = 8.4 Hz, 1H, H-δ), 7.359 (t, *J* = 33.0 Hz, 2H, Ar–H^4^, Ar–H^6^), 7.311 (d, *J* = 7.8 Hz, 1H, H-α), 7.072–7.026 (m, 1H, Ar–H^3^), 6.982 (t, *J* = 15.0 Hz, 1H, Ar–H^5^), 6.922 (d, *J* = 8.4 Hz, 1H, Ar–H^5′^), 6.564 (dd, *J* = 1.8 Hz, *J* = 6.6 Hz, 1H, H-β), 6.500 (d, *J* = 2.4 Hz, 1H, Ar–H^3′^) 3.913 (s, 3H, 2-OCH_3_), 3.884 (d, *J* = 4.8 Hz, 6H, 2′-OCH_3_, 4′-OCH_3_), 3.012 (d, *J* = 6.0 Hz, 2H, CH_2_), 2.931 (d, *J* = 5.4 Hz, 2H, CH_2_). LC–MS *m*/*z*: 377.17 (M + H)^+^, calcd for C_24_H_24_O_4_: 376.17.

**(E)-2-((E)-2-methoxybenzylidene)-5-((E)-3-(2-methoxyphenyl)allylidene)cyclopentan-1-one (10):** Yellow powder, 68% yield, mp 155.9–156.9 °C. ^1^H NMR (600 MHz, CDCl_3_), *δ*: 7.965 (s, 1H, H-ɤ), 7.588 (d, *J* = 7.8 Hz, 1H, Ar–H^6^), 7.588 (d, *J* = 7.2 Hz, 1H, Ar–H^6′^), 7.393 (t, *J* = 14.4 Hz, 2H, Ar–H^4^, Ar–H^4′^), 7.312 (t, *J* = 15.6 Hz, 1H, H-δ), 7.062 (t, *J* = 15.6 Hz, 1H, H-α), 7.026 (d, *J* = 7.2 Hz, 1H, Ar–H^3^), 6.920–7.008 (m, 4H, Ar–H^5′^, Ar–H^5^, Ar–H^3′^, H-β), 3.912 (d, *J* = 6.6 Hz, 6H, 2-OCH_3_, 2′-OCH_3_), 3.038 (d, *J* = 4.8 Hz, 2H, CH_2_), 2.934 (d, *J* = 4.8 Hz, 2H, CH_2_). LC–MS *m*/*z*: 347.20 (M + H)^+^, calcd for C_23_H_22_O_3_: 346.16.

**(E)-2-((E)-3-(2-methoxyphenyl)allylidene)-5-((E)-4-morpholinobenzylidene)cyclopentan-1-one (11):** Orange powder, 68.5% yield, mp 213.9–215.5 °C. ^1^H NMR (600 MHz, CDCl_3_), *δ*: 7.588 (d, *J* = 7.8 Hz, 1H, H-ɤ), 7.559 (d, *J* = 8.4 Hz, 2H, Ar–H^4^, Ar–H^6^), 7.508 (s, 1H, H-δ), 7.364 (t, *J* = 30.6 Hz, 2H, Ar–H^2′^, Ar–H^6′^), 7.308 (t, *J* = 15.6 Hz, 1H, H-α), 7.077–7.032 (m, 1H, Ar–H^5^), 6.986 (t, *J* = 15.0 Hz, 1H, Ar–H^3^), 6.953–6.918 (m, 3H, Ar–H^5′^, Ar–H^3′^, H-β), 3.915 (s, 3H, 2-OCH_3_), 3.891 (t, *J* = 9.6 Hz, 8H, CH_2_–O–CH_2_, CH_2_–N–CH_2_), 3.063 (d, *J* = 6.0 Hz, 2H, CH_2_), 2.959 (d, *J* = 4.8 Hz, 2H, CH_2_). LC–MS *m*/*z*: 402.16 (M + H)^+^, calcd for C_26_H_27_NO_3_: 401.20.

**(E)-2-((E)-2,3-dimethoxybenzylidene)-5-((E)-3-(2-methoxyphenyl)allylidene)cyclopentan-1-one (12):** Yellow powder, 61.3% yield, mp 142.8–144.8 °C. ^1^H NMR (600 MHz, CDCl_3_), *δ*: 7.878 (s, 1H, H-ɤ), 7.589 (d, *J* = 7.8 Hz, 1H, H-δ), 7.393 (t, *J* = 25.8 Hz, 2H, Ar–H^4^, Ar–H^6^), 7.327–7.301 (m, 1H, Ar–H^6′^), 7.192 (d, *J* = 7.8 Hz, 1H, H-α), 7.123 (t, *J* = 15.6 Hz, 1H, Ar–H^3^), 7.077–7.032 (m, 1H, Ar–H^5′^), 6.999–6.968 (m, 2H, Ar–H^5^, Ar–H^4′^), 6.928 (d, *J* = 8.4 Hz, 1H, H-β), 3.917 (d, *J* = 2.4 Hz, 6H, 2′-OCH_3_, 3′-OCH_3_), 3.885 (s, 3H, 2-OCH_3_), 3.027 (t, *J* = 7.2 Hz, 2H, CH_2_), 2.934 (d, *J* = 6.0 Hz, 2H, CH_2_). LC–MS *m*/*z*: 377.23 (M + H)^+^, calcd for C_24_H_24_O_4_: 376.17.

**(E)-2-((E)-3,4-dihydroxybenzylidene)-5-((E)-3**-**(2-methoxyphenyl)allylidene)cyclopentan-1-one (13):** Yellow powder, 56% yield, mp 247.7–248.1 °C. ^1^H NMR (600 MHz, DMSO), *δ*: 9.584 (s, 1H, 3′-OH), 9.211 (s, 1H, 4′-OH), 7.690 (dd, *J* = 1.8 Hz, 6.6 Hz, 1H, H-ɤ), 7.330–7.280 (m, 2H, Ar–H^6^, Ar–H^4^), 7.192 (t, *J* = 2.4 Hz, 1H, H-δ), 7.167–7.123 (m, 1H, H-α), 7.106–7.079 (m, 2H, Ar–H^3^, Ar–H^2′^), 7.040 (d, *J* = 7.8 Hz, 1H, Ar–H^5′^), 6.995–6.954 (m, 2H, Ar–H^5^, Ar–H^6′^), 6.820 (d, J = 8.4 Hz, 1H, H-β), 3.844 (s, 3H, 2-CH_3_), 2.959–2.905 (m, 4H, CH_2_). LC–MS *m*/*z*: 349.18 (M + H)^+^, calcd for C_22_H_20_O_4_: 348.14.

**(E)-2-((E)-2,5-dimethoxybenzylidene)-5-((E)-3-(2-methoxyphenyl)allylidene)cyclopentan-1-one (14):** Yellow powder, 57.9% yield, mp 168.0–169.2 °C. ^1^H NMR (600 MHz, CDCl_3_), *δ*: 7.919 (s, 1H, H-ɤ), 7.587 (d, *J* = 7.2 Hz, 1H, H-δ), 7.381 (t, *J* = 25.2 Hz, 2H, Ar–H^4^, Ar–H^6^), 7.312 (t, *J* = 15.0 Hz, 1H, Ar–H^3^), 7.121 (d, *J* = 2.4 Hz, 1H, H-α), 7.073–7.027 (m, 1H, Ar–H^5^), 6.985 (t, *J* = 15.0 Hz, 1H, Ar–H^3′^), 6.932–6.888 (m, 2H, Ar–H^6′^, Ar–H^4′^), 6.881 (d, *J* = 9.0 Hz, 1H, H-β), 3.916 (s, 3H, 2-OCH_3_), 3.849 (d, *J* = 16.2 Hz, 6H, 2′-OCH_3_, 5′-OCH_3_), 3.046 (t, *J* = 7.2 Hz, 2H, CH_2_), 2.934 (d, *J* = 5.4 Hz, 2H, CH_2_). LC–MS *m*/*z*: 377.23 (M + H)^+^, calcd for C_24_H_24_O_4_: 376.17.

**(E)-2-((E)-4-(bis(2-chloroethyl)amino)benzylidene)-5-((E)-3-(2-methoxyphenyl)allylidene)cyclopentan-1-one (15):** Red powder, 66.9% yield, mp 150.7–151.5 °C. ^1^H NMR (600 MHz, CDCl_3_), *δ*: 7.592–7.552 (m, 3H, H-ɤ, Ar-H^2′^, Ar-H^6′^), 7.495 (s, 1H, H-δ), 7.381–7.288 (m, 3H, Ar–H^4^, Ar–H^6^, H-α), 7.081–7.036 (m, 1H, Ar–H^5^), 6.986 (t, *J* = 15.0 Hz, 1H, Ar–H^3^), 6.926 (d, *J* = 8.4 Hz, 1H, H-β), 6.754 (d, *J* = 8.4 Hz, 2H, Ar–H^3′^, Ar–H^5′^), 3.917 (s, 3H, 2-OCH_3_), 3.824 (t, *J* = 13.8 Hz, 4H, CH_2_–N–CH_2_), 3.692 (t, *J* = 13.8 Hz, 4H, ClCH_2_×2), 3.049 (d, *J* = 5.4 Hz, 2H, CH_2_), 2.962 (d, *J* = 6.0 Hz, 2H, CH_2_). LC–MS *m*/*z*: 456.13 (M + H)^+^, calcd for C_26_H_27_Cl_2_NO_2_: 455.14.

**(E)-2-((E)-3-(2-methoxyphenyl)allylidene)-5-((E)-3,4,5-trimethoxybenzylidene)cyclopentan-1-one (16):** Yellow powder, 58% yield, mp 172.6–173.3 °C. ^1^H NMR (600 MHz, CDCl_3_), *δ*: 7.597 (t, *J* = 1.2 Hz, 1H, H-γ), 7.465 (t, *J* = 4.8 Hz, 1H, Ar–H^6′^), 7.408 (t, *J* = 26.4 Hz, 2H, H-α × 2), 7.318 (td, *J* = 1.2 Hz, *J* = 7.2 Hz, 1H, Ar–H^4′^), 7.076–7.031 (m, 1H, Ar–H^5′^), 6.988 (t, *J* = 15.0 Hz, 1H, Ar–H^3′^), 6.928 (d, *J* = 8.4 Hz, 1H, H-β), 6.854 (s, 2H, Ar–H^2^, Ar–H^6^), 3.924 (t, *J* = 9.6 Hz, 12H, 3-OCH_3_, 4-OCH_3_, 5-OCH_3_, 2′-OCH_3_), 3.119–3.091 (m, 2H, CH_2_), 2.974 (t, *J* = 6.6 Hz, 2H, CH_2_). LC–MS *m*/*z*: 407.18 (M + H)^+^, calcd for C_25_H_26_O_5_: 406.18.

**(E)-2-((E)-2-hydroxy-3-methoxybenzylidene)-5-((E)-3-(2-methoxyphenyl)allylidene)cyclopentan-1-one (17):** Brown powder, 58% yield, mp 236.8–237.6 °C. ^1^H NMR (600 MHz, CDCl_3_), *δ*: 7.562 (dd, *J* = 1.2 Hz, 6 Hz, 1H, H-ɤ), 7.446 (t, *J* = 5.4 Hz, 1H, Ar–H^6^), 7.361 (d, *J* = 15.6 Hz, 2H, Ar–H^4^, H-δ), 7.302–7.274 (m, 1H, H-α), 7.221 (d, *J* = 2.4 Hz, 1H, Ar–H^2′^), 7.124 (dd, *J* = 1.8 Hz, 6.6 Hz, 1H, Ar–H^3^), 7.055–7.010 (m, 1H, Ar–H^3′^), 6.962 (t, *J* = 15Hz, 1H, Ar–H^5^), 6.904 (d, *J* = 8.4 Hz, 2H, Ar–H^4′^, H-β), 3.943 (s, 3H, 2-OCH_3_), 3.893 (s, 3H, 5′-OCH_3_), 3.055–3.038 (m, 2H, CH_2_), 2.940 (t, *J* = 7.2 Hz, 2H, CH_2_). LC–MS *m*/*z*: 363.21 (M + H)^+^, calcd for C_23_H_22_O_4_: 362.15.

**2-methoxy-4-((E)-((E)-3-((E)-3-(2-methoxyphenyl)allylidene)-2-oxocyclopentyliden)methyl)phenyl acrylate (18)**: Brick red power, 58.4% yield, mp 74.2–76.5 °C. ^1^H NMR (400 MHz, CDCl3), *δ*: 9.279 (d, 1H, *J* = 7.6 Hz, 1H, H-δ), 7.482 (s, 1H, H-β′), 7.406–7.354 (m, 2H, Ar–H^6′^, H-β), 7.295 (t, *J* = 7.2 Hz, 1H, Ar–H^4^), 7.214 (d, *J* = 8.0 Hz, 1H, Ar–H^1^), 7.175–7.111 (m, 2H, Ar–H^2’^, Ar–H^5’^), 7.066–7.027 (m, 1H, Ar–H^3^), 6.998–6.945 (m, 1H, Ar–H^5^), 6.904 (d, *J* = 8.4 Hz, 1H, CH–CO), 6.654–6.592 (m, 1H, H-γ), 6.396–6.312 (m, 1H, CH), 6.039 (d, J = 10.4 Hz, 1H, CH), 3.892 (s, 3H, 2-OCH_3_), 3.873 (s, 3H, 3′-OCH_3_), 3.065 (d, *J* = 4.4 Hz, 2H, CH_2_), 2.951 (d, *J* = 6.0 Hz, 2H, CH_2_). LC–MS *m*/*z*: 417.2 (M + H)^+^, calcd for C_23_H_22_O_4_: 416.16.

**2-methoxy-4-((E)-((E)-3-((E)-3-(2-methoxyphenyl)allylidene)-2-oxocyclopentyliden)methyl)phenyl 2-phenylacetate (19):** Khaki power, 33.6% yield, mp 90.1–92.9 °C. ^1^H NMR (400 MHz, CDCl3), *δ*: 7.571–7.548 (dd, *J* = 1.6 Hz, *J* = 6.4 Hz, 1H, δ-H), 7.455 (t, *J* = 2.4 Hz, 1H, Ar–H^4^), 7.419–7.386 (m, 3H, Ar–H^6^, H-β′, H-β), 7.368–7.336 (m, 3H, Ar–H^3″^, Ar–H^5″^), 7.304 (t, *J* = 6.8 Hz, 2H, Ar–H^4″^, Ar–H^6″^), 7.167 (d, *J* = 8.4 Hz, 1H, Ar–H^2″^), 7.129 (s, 1H, Ar–H^2′^), 7.066–7.019 (m, 2H, Ar–H^5^, Ar–H^5’^), 6.990–6.941 (m, 1H, H-γ), 6.901 (d, *J* = 8.0 Hz, 1H, Ar–H^3^), 3.904 (s, 2H, CH_2_–CO), 3.889 (s, 3H, 2-OCH_3_), 3.802 (s, 3H, 3′-OCH_3_), 3.038 (d, *J* = 4.0 Hz, 2H, CH_2_), 2.939 (d, *J* = 4.0 Hz, 2H, CH_2_). LC–MS *m*/*z*: 481.2 (M + H)^+^, calcd for C_23_H_22_O_4_: 480.19.

**2-methoxy-4-((E)-((E)-3-((E)-3-(2-methoxyphenyl)allylidene)-2-oxocyclopentyliden)methyl)phenyl cyclopropanecarboxylate (20):** Yellow power 70.5% yield, mp 145.0–146.8 °C. ^1^H NMR (400 MHz, CDCl3), *δ*: 7.576–7.554 (dd, *J* = 1.2 Hz, *J* = 6.4 Hz, 1H, H-δ), 7.466 (t, *J* = 2.8 Hz, 1H, H-β), 7.400–7.345 (m, 2H, Ar–H^6^, Ar–H^4^), 7.293(t, *J* = 7.2 Hz, 1H, H-β′), 7.189 (d, *J* = 8.4 Hz, 1H, Ar–H-6′), 7.147 (s, 1H, Ar–H^2^), 7.099 (d, *J* = 8.4 Hz, 1H, Ar–H^5’^), 7.062–7.023 (m, 1H, Ar–H^5^), 6.994–6.943 (m, 1H, H-γ), 6.902 (d, *J* = 8.0 Hz, 1H, Ar–H^3^), 3.890 (s, 3H, 2-OCH_3_), 3.876 (s, 3H, 3′-OCH_3_), 3.052 (d, *J* = 4.0 Hz, 2H, CH_2_), 2.947 (d, *J* = 10.0 Hz, 2H, CH_2_), 1.920–1.867 (m, 1H, CH–CO) 1.222–1.185 (m, 2H, CH_2_), 1.062–1.015 (m, 2H, CH_2_). LC–MS *m*/*z*: 431.2(M + H)^+^, calcd for C_27_H_26_O_5_ 430.18.

### Synthetic procedures

#### General synthetic procedure for the synthesis of compound 1b

A solution comprising cyclopentanone (**1a**, 20 mmol), morpholine (30 mmol), and 4-methylbenzenesulfonic acid (200 mg) in cyclohexane (20 ml) was heated to reflux at 90 °C for 5 h. After cooling at room temperature, the mixture was concentrated to obtain the enamine intermediate **1b** as a brown oil, which was directly utilised for the next reaction without undergoing purification.

#### General procedure for the synthesis of compound 1d

A mixture of **1b** (10 mmol) and various substituted aromatic aldehydes (10 mmol) was dissolved in ethanol (20 ml). The resulting solution was stirred at 78 °C for 30 min. After cooling to room temperature, a 10% hydrochloric acid solution was employed to adjust the pH to an acidic range. The reaction mixture was stirred at room temperature for 3 h. Ethanol was removed using a rotary evaporator, followed by extraction with ethyl acetate. The product was subsequently purified through column chromatography to obtain intermediate compound **1d**.

#### General procedure for the synthesis of compounds 1–17

A mixture of **1d** (10 mmol) and *o*-methoxycinnamaldehyde (12.5 mmol) was dissolved in ethanol (20 ml). Then NaOH or HCl (gas) was added to the solution to catalyse the reaction. The reaction mixture was stirred at room temperature until aldehyde was consumed (usually 6–12 h). If the product precipitated upon quenching with cold water, it was filtered and further crystallised using hot ethanol. Alternatively, in cases where precipitation did not occur, the product was purified using silica gel chromatography.

#### General procedure for the synthesis of compounds 18–20

A mixture of compound **2** (10 mmol), acid chloride (15 mmol), and three drops of triethylamine in anhydrous dimethyl sulfoxide (5 ml) was prepared under 0 °C. The mixture was stirred at room temperature for 2 h, poured into ice water, extracted with dichloromethane twice, and dried over MgSO_4_. The crude product was purified by silica gel chromatography using a hexane and ethyl acetate gradient to obtain the desired products **18–20**.

### Biological evaluation

#### Cell cultures

Human gastric cancer cell lines SGC-7901 and BGC-823, as well as normal human liver cell line MIHA and normal rat kidney cell NRK were purchased directly from China Centre for Type Culture Collection (Wuhan, China). Both the SGC-7901 and BGC-823 cells were cultured in RPMI-1640 medium (Gibco), supplemented with 100 U/ml penicillin, 100 mg/ml streptomycin (Gibco), and 10% foetal bovine serum (FBS, Gibco). All cell lines were grown in an incubator, under an atmosphere containing 5% CO_2_, at 37 °C. Our experimental procedures were conducted until these cells reached the logarithmic growth phase.

#### MTT assay

For cell viability assessments, SGC-7901 and BGC-823 cells were seeded at a density of 3000 cells/well, while MIHA and NRK cells were seeded at 5000 and 800 cells/well, respectively, in a 96-well cell culture plate. After an initial incubation of 24 h, the cells were exposed to the test compounds for various time intervals, including 12, 24, 48, or 72 h. Subsequently, a solution of MTT (5 mg/mL, 20 μL) was added to each well. Following a 4-h incubation period, the intracellular formazan crystals were dissolved in 150 μL DMSO. The absorbance (A) of the resulting solution was measured at 490 nm using an enzyme-labelled metre (MD, USA). The inhibitory rate was calculated as [(1 − -*A*_compound-treated group_)/*A*_control group_)] × 100%. IC_50_ was calculated using GraphPad Prism 6 software.

#### RF-based QSAR model

The QSAR model was built using an RF algorithm. The molecular structure of the compounds, encoded as Simplified Molecular Input Line Entry System (SMILES) notations, was processed using the open-source toolkit RDKit to calculate a set of 208-dimensional 2D descriptor information based on the physicochemical properties and molecular structures such as molecular weights, topologically polar surface areas, lipid-water partition coefficients, and so on. The processed molecular descriptors and experimental data values are fed into the RF algorithm for training to obtain a QSAR model. The model implemented based on the Scikit-learn machine learning library in Python language, and all parameters are used as default parameters.

#### Colony formation assay

SGC-7901 cells were seeded at a density of 2 × 10^3^/well in a 6-well cell culture plate. Following an overnight incubation for initial growth, cells were exposed to the test compounds for 10 h. Subsequently, the culture medium was replaced with a fresh RPMI-1640 medium, and the cells were allowed to grow for approximately 8 days. They were then fixed with 4% paraformaldehyde, followed by staining with crystal violet. Images were captured using a camera.

#### Scratch wound-healing assay

SGC-7901 cells were seeded at a density of 2 × 10^6^/well in a 6-well cell culture plate and cultured for 24 h. Subsequently, scratches were created using a sterile pipette tip. The cells were then treated with the test compounds for 48 h. Images were captured at specified time points using a microscopic camera device (Nikon, Tokyo, Japan). The wound region was determined by tracing the cell-free areas in the images using the ImageJ program.

#### Apoptosis assay

SGC-7901 cells were seeded at a density of 2 × 10^6^/well in a 6-well cell culture plate. After an overnight incubation for initial growth, cells were exposed to the test compounds for 18 h. Cells were then collected using pancreatin without ethylenediaminetetraacetic acid. Cells were stained with fluorescein isothiocyanate-annexin V and propidium iodide for 15 min, protected from light. Analysis was immediately conducted using a FACSCalibur flow cytometer (BD Biosciences, CA).

#### Cell cycle analysis

SGC-7901 cells were seeded at a density of 2 × 10^6^/well in a 6-well cell culture plate. Cells were treated with the test compounds for 48 h, followed by PBS washing. The cells were fixed in 75% ice-cold ethanol for 4 h. After fixation, cells were stained with 500 µL of propidium iodide solution containing RNase (550825, BD Biosciences 35 Clontech, San Jose, CA, USA) for 10 min at 4 °C in the absence of light. The mixture was filtered through a 200­mesh gauze. Cell cycle analysis was performed using a FACSCalibur flow cytometer (BD Biosciences, CA).

#### Western blot

SGC-7901 cells were seeded at a density of 3 × 10^6^/well in a 6-well cell culture plate. Cells are lysed with RIPA buffer (BOSTER, China) containing 1% phosphatase inhibitor (P1260, Solarbio, Beijing). The protein concentration of all samples was determined by using the Bradford protein assay kit (Bio-Rad, Hercules, CA). Lysates were separated by SDS-PAGE electrophoresis and then transferred to PVDF membranes (Millipore, Billerica, MA, USA). The membrane was blocked with 5% non-fat milk at room temperature for 90 min, eluted with TBST, and incubated with primary antibodies overnight at 4 °C. After washing three times with TBST, the membrane was incubated with the peroxidase (HRP)-conjugated secondary antibody for 90 min at room temperature. Detection was performed using ECL kit (Bio-Rad, Hercules, CA) and bands were analysed with Image Lab software (NIH, Bethesda, MD, USA). The following primary antibodies were used: GAPDH (1:1000, 20536-1-AP, Proteintech, China), Caspase-3 (1:1000, 9662S, Cell Signaling Technology, USA), STAT3 (1:1000, 12640S, Cell Signaling Technology, USA), Phospho-STAT3(Tyr705) (1:1000, 9145S, Cell Signaling Technology, USA), Phospho-AKT(Ser473) (1:1000, 4060S, Cell Signaling Technology, USA), AKT (1:1000, 4691S, Cell Signaling Technology, USA). The following secondary antibodies were used: HRP-conjugated Affinipure Goat Anti-Rabbit IgG(H + L) antibody (1:5000, SA00001-2, Proteintech, China).

#### Animals

All animals used in this study were purchased from Shanghai Slaccas Lab Animal Co. Ltd (China). Male C57BL/6 mice (6–8 weeks old) and BALB/C-nu mice (6–8 weeks old) were housed on a 12 h light/dark cycle and provided with free access to water and diet.

#### The in vivo assessment of the anti-tumour effect

The *in vivo* anti-tumour efficacy of compound **2** was evaluated using a xenograft mice study. Following the acclimatisation of BALB/C-nu mice, approximately 5 × 10^6^ SGC-7901 cells were injected into the right abdomen. The cells were allowed to grow for approximately 5 days until tumours reached visible and measurable sizes. Prior to administration, compounds were dissolved in a solution (90% PBS, 6% castor oil, and 4% DMSO). Compound **2** at a dosage of 15 mg/kg (*n* = 6), curcumin at 20 mg/kg (*n* = 6), and vehicle (control group, *n* = 6) were injected daily for 20 days by intraperitoneal injection. The tumour volume and animal body weight were measured daily. Tumour size = length × width^2^/2. The mice were euthanized on the last day, and the tumours were collected and weighed. The experimental procedures were conducted with the approval of the Ethics Committee of Wenzhou Medical University (wydw2022-0480).

#### In vivo toxicity assessment

The assessment of the toxicity of compound **2** was carried out using male C57BL/6 mice aged 6–8 weeks and weighing 18–22 g. The mice were randomly divided into three groups: control group (*n* = 4), compound **2-**treated group (*n* = 6), and EF24-treated group (*n* = 6). The active compounds (600 mg/kg) were administered to the mice through intraperitoneal (i.p.) injection on day 1. Subsequently, all mice were observed for 15 days, during which their body weights and mortality were recorded. Finally, these mice were sacrificed.

#### Statistical analysis

Results are presented as the mean ± standard error (SEM). Statistical analysis was performed using GraphPad Prism 6 software. *p* values <0.05 were established as the threshold for statistical significance. *p* values were calculated using the Student’s *t*-test.

## Supplementary Material

Supplemental Material
