# Supplementary material for: Design, synthesis, and evaluation of cyclic C7-bridged monocarbonyl curcumin analogs containing an o-methoxy phenyl group as potential agents against gastric cancer
Source: J Enzyme Inhib Med Chem. 2024 Feb 22;39(1):2314233. doi: 10.1080/14756366.2024.2314233 (PMC10885745; doi:10.1080/14756366.2024.2314233)
Supplement: Supplemental Material [file IENZ_A_2314233_SM0173.pdf]

Design, synthesis, and evaluation of cyclic C7 bridged monocarbonyl curcumin analogues inspired *o*-methoxy phenyl group as potential anti-gastric cancer agents

Supplementary data

1. Synthetic procedures of compound a-g

1. 1 General procedure for synthesis of compound a-c

Ketone (2 mmol) and (substituted) benzaldehyde (4 mmol) were condensed at room temperature with ethanol (20 ml) as solvent, 40% NaOH solution or 10% HCl solution (0.2 mmol) as catalyst. After 2-3 hours, a large number of solid precipitations were extracted. The solids were extracted and washed with anhydrous ethanol (15 ml). The filter residues were dried and purified by colu mn chromatography (EA/PE=1:5) to obtain yellow solid.

1.2 General procedure for synthesis of compound d

Acetyl acetone (2moml) and boric anhydride (10moml) were dissolved in eth yl acetate (10ml), then benzaldehyde (4mmol) and tributyl borate (9mmol) were added, and ethyl acetate solution of n-butylamine (1.4mmol) was slowly added. Reaction at 40°C, 4h later, the reaction ended. Adding 10% HCI, adjust pH t o 5, extracting with ethyl acetate and water, removing solvent, column chromat ography (EA/PE=1:20), yellow solid was obtained

1.3 General procedure for synthesis of compound e-g

Ketone (2mmol) and cinnamaldehyde (10mmol) were dissolved in DMSO, pr oline (1mmol) was added, and the reaction was carried out overnight at room temperature. After the reaction, add 2 drops of concentrated hydrochloric acid, stirring for 30min, and then adding pure water (50ml) to precipitate yellow sol id, and extracting and filter the initial product. The product obtained in the pre vious step (2mmol, 1eq) and benzaldehyde (4mmol,2eq) were dissolved in anhy drous ethanol, and 8 drops of 40%NaOH solution were added. After reaction a t room temperature for 1-2h, a large amount of yellow solid was precipitated. After extraction and filtration, the solvent was removed and the final product y ellow solid was obtained by column chromatography (EA/PE=1:8).

The chemical data of compounds are presented as follows:

2,5-di((E)-benzylidene)cyclopentan-1-one (a): Light yellow powder, 87.3% yield, m.p. 189.2-190.3 °C. ^{1}H NMR (600 MHz, $CDCl_3$), δ: 7.62-7.60 (m, 6H, Ar-H^2, Ar-$H^{2'}$, Ar-H^6, Ar-$H^{6'}$, α-H×2), 7.45 (t, *J*=7.5Hz, 4H, Ar-H^3, Ar-$H^{3'}$, Ar-H^5, Ar-$H^{5'}$), 7.41–7.37 (m, 2H, Ar-H^4, Ar-$H^{4'}$), 3.13 (s, 4H, CH_2×2). LC-MS m/z: 261.2 $(M+H)^+$, calcd for $C_{19}H_{16}O$: 260.12.

2,6-di((E)-benzylidene)cyclohexan-1-one (b): Light yellow powder, 85.0% yield, m.p. 113.8-115.2 °C. ^{1}H NMR (600 MHz, $CDCl_3$), δ: 7.81 (s, 2H, α-H×2), 7.47 (d, *J*=7.4Hz, 4H, Ar-H^2, Ar-$H^{2'}$, Ar-H^6, Ar-$H^{6'}$), 7.41 (t, *J*=7.6Hz, 4H, Ar-H^3, Ar-$H^{3'}$, Ar-H^5, Ar-$H^{5'}$), 7.34 (t, *J*=7.3Hz, 2H, Ar-H^4, Ar-$H^{4'}$), 2.96-2.92 (m, 4H, CH_2×2), 1.82-1.78 (m, 2H, CH_2). LC-MS m/z: 275.1 $(M+H)^+$, calcd for $C_{20}H_{18}O$: 274.14.

(1E,4E)-1,5-diphenylpenta-1,4-dien-3-one (c): Light yellow powder, 91.6% yield, m.p. 107.5-109.3 °C. ^{1}H NMR (600 MHz, $CDCl_3$), δ: 7.75 (d, *J*=15.9Hz, 2H, β-H×2), 7.63 (dd, *J*=6.4, 2.6Hz, 4H, Ar-H^2, Ar-$H^{2'}$, Ar-H^6, Ar-$H^{6'}$), 7.45-7.39 (m, 6H, Ar-H^3, Ar-$H^{3'}$, Ar-H^4, Ar-$H^{4'}$, Ar-H^5, Ar-$H^{5'}$), 7.10 (d, *J*=15.9Hz, 2H, α-H×2). LC-MS m/z: 235.1 $(M+H)^+$, calcd for $C_{17}H_{14}O$: 234.10.

(1E,6E)-1,7-diphenylhepta-1,6-diene-3,5-dione (d): Orange-red powder, 45.0% yield, m.p. 124.1-126.3 °C. ^{1}H NMR (600 MHz, $CDCl_3$), δ 7.76 (dd, *J*=7.3, 2.3 Hz, 4H, Ar-H^2, Ar-$H^{2'}$, Ar-H^6, Ar-$H^{6'}$), 7.69 (d, *J*=15.8 Hz, 2H, β-H×2), 7.47 (m, 6H, Ar-H^3, Ar-$H^{3'}$, Ar-H^5, Ar-$H^{5'}$, Ar-H^4, Ar-$H^{4'}$), 6.99 (d, *J*=16.0 Hz, 2H, α-H×2,), 6.24 (s, 1H, OH-HC=C=O). LC-MS m/z: 277.1 $(M+H)^+$, calcd for $C_{20}H_{18}O$: 276.12.

(E)-2-((E)-benzylidene)-5-((E)-3-phenylallylidene)cyclopentan-1-one (e): Yellow powder, 43.0% yield, m.p. 179.2-182.6 °C. ^{1}H NMR (400 MHz, DMSO) δ 7.69 (t, *J*=8.5Hz, 4H, Ar'-H^2, Ar'-H^6, Ar-H^2, Ar-H^6), 7.52 (d, *J*=7.1Hz, 2H, β'-H, δ'-H), 7.50-7.41 (m, 4H, Ar-H^3, Ar-H^4, Ar-H^5, Ar'-H^3), 7.39 (d, *J*=3.2Hz, 1H, γ'-H), 7.34 (t, *J*=7.5Hz, 1H, Ar'-H^5), 7.21 (s, 2H, β-H, Ar'-H^4), 3.23-3.04 (m, 2H, CH_2), 3.01 – 2.88 (m, 2H, CH_2). LC-MS m/z: 287.2 $(M+H)^+$, calcd for $C_{21}H_{18}O$: 286.14.

(E)-2-((E)-benzylidene)-6-((E)-3-phenylallylidene)cyclohexan-1-one (f): Yellow powder, 45.0% yield, m.p. 113.5-114.7 °C. 1H NMR (400 MHz, $CDCl_3$) δ 7.82 (s, 1H, β-H), 7.59-7.51 (m, 3H, γ'-H, Ar-H^2, Ar-H^3), 7.49 (d, *J*=7.3 Hz, 2H, β'-H, δ'-H), 7.38 (ddt, *J*=14.4, 9.7, 7.2 Hz, 6H, Ar'-H^4, Ar'-H^5, Ar'-H^6, Ar-H^4, Ar-H^5, Ar-H^6), 7.22-6.91 (m, 2H, Ar'-H2, Ar'-H3), 2.95 (t, *J*=5.2 Hz, 2H, CH2), 2.87 (t, *J*=5.6 Hz, 2H, CH2), 1.95-1.79 (m, 2H,CH2). LC-MS m/z: 301.2 $(M+H)^+$, calcd for $C_{22}H_{20}O$: 300.15.

(1E,4E,6E)-1,7-diphenylhepta-1,4,6-trien-3-one (g): Deep yellow powder, 47.8% yield, m.p. 139.2-141.3 °C. ^{1}H NMR (400 MHz, $CDCl_3$) δ 7.74 (d, *J*=16.0 Hz, 1H, α'-H), 7.64 (d, *J*=3.7 Hz, 2H, δ'-H, Ar-H^2), 7.54 (d, *J*=7.0 Hz, 2H, Ar-H^6, Ar'-H^6), 7.45-7.32 (m, 7H, α-H, β-H, β'-H, γ'-H, Ar-H^4, Ar-H^5, Ar'-H^4), 7.05 (t, *J*=13.5 Hz, 3H, Ar'-H^3, Ar'-H^5, Ar-H^3), 6.68 (d, *J*=15.2 Hz, 1H, Ar'-H^2). LC-MS m/z: 261.2 $(M+H)^+$, calcd for $C_{19}H_{16}O$: 260.12.

MS spectrum data and ^{1}H-NMR spectrum (a-g and 1-20)

a.

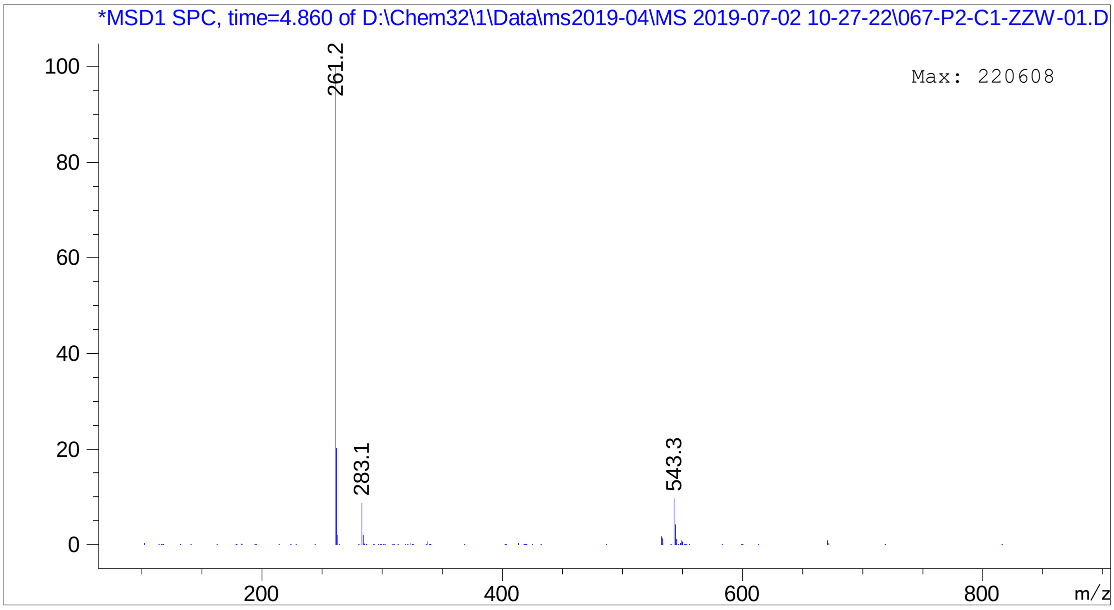

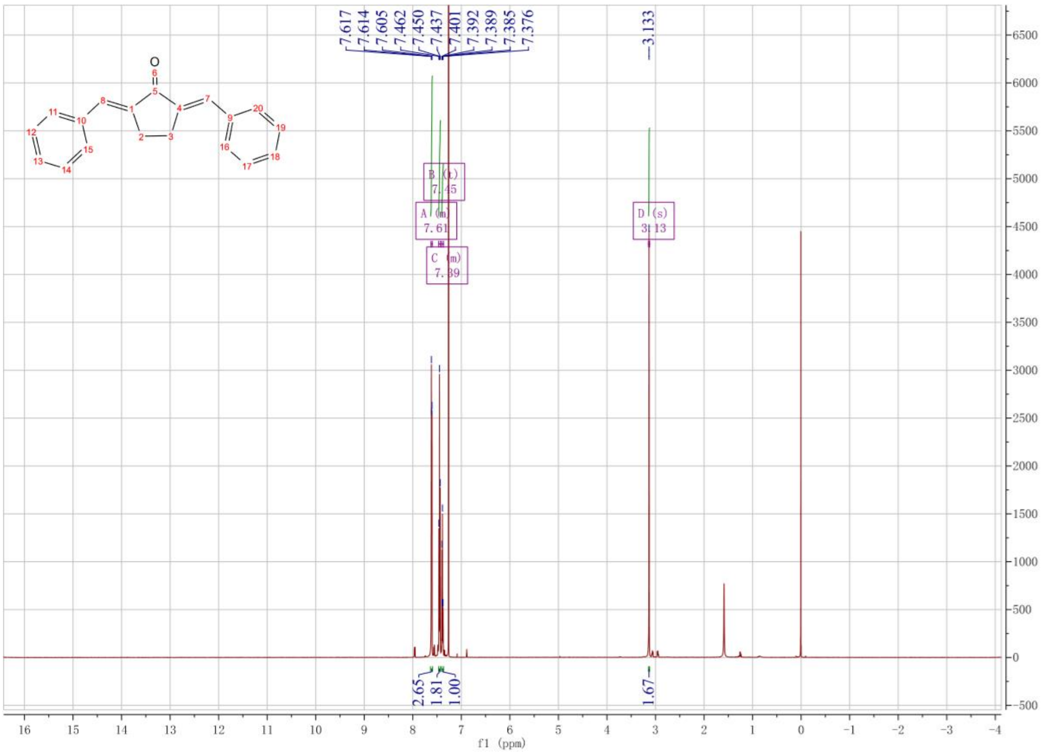

b.

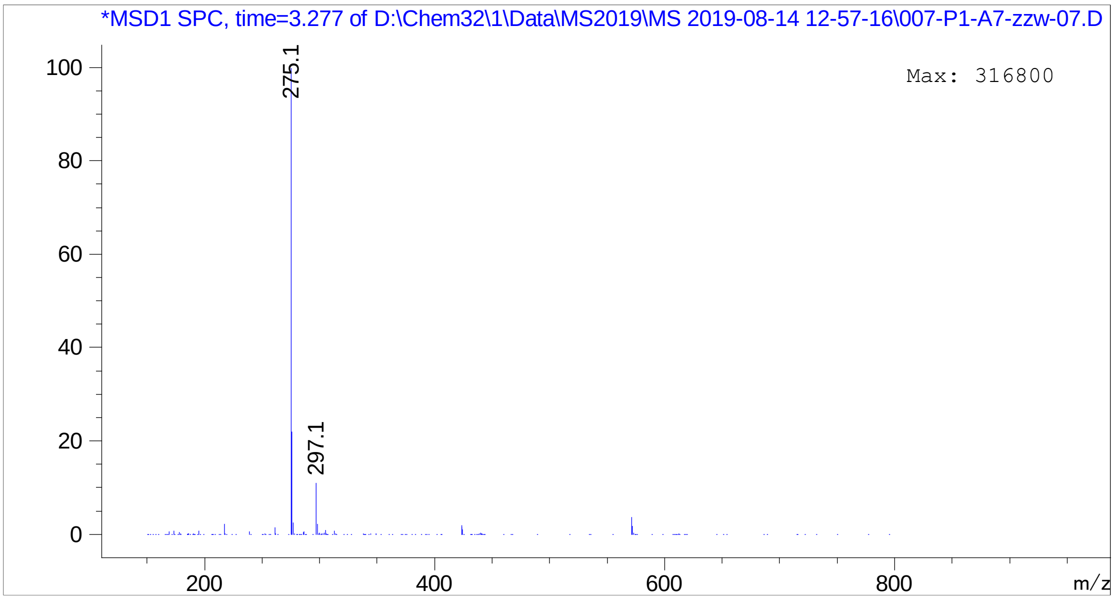

*MSD1 SPC, time=3.277 of D:\Chem32\1\Data\MS2019\MS 2019-08-14 12-57-16\007-P1-A7-zzw-07.D
Max: 316800
275.1
297.1
m/z

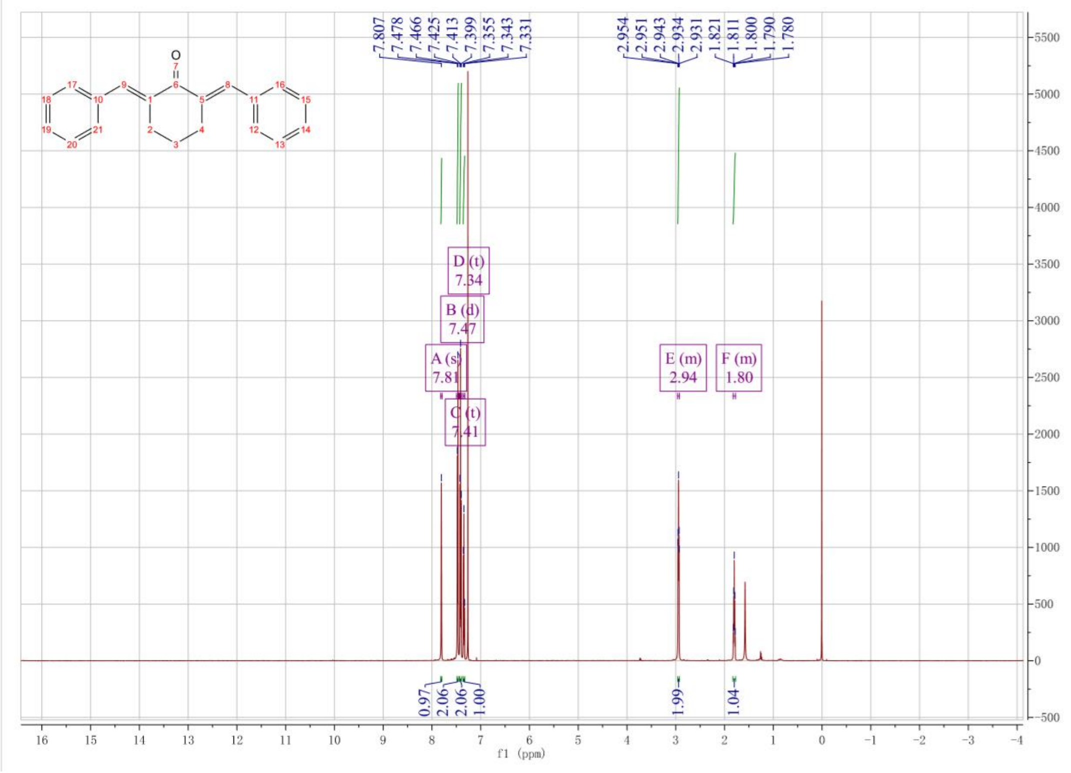

7.807 7.478 7.466 7.425 7.413 7.399 7.355 7.343 7.331
2.954 2.951 2.943 2.934 2.931 1.821 1.811 1.800 1.790 1.780
A (s) 7.81
B (d) 7.47
C (t) 7.41
D (t) 7.34
E (m) 2.94
F (m) 1.80
0.97 2.06 2.06 1.00 1.99 1.04
f1 (ppm)

c.

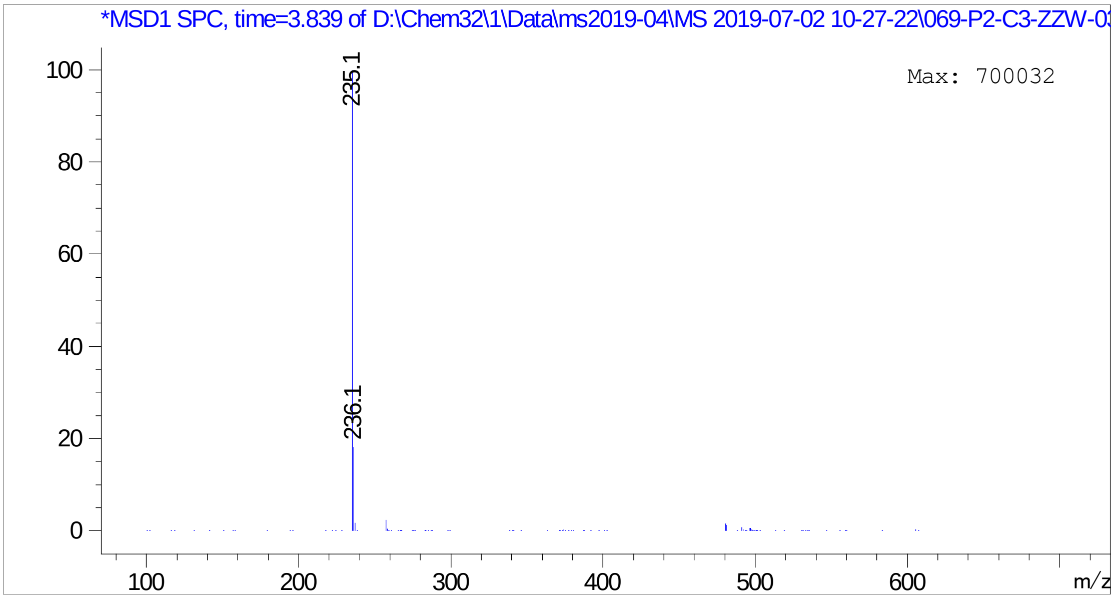
*MSD1 SPC, time=3.839 of D:\Chem32\1\Data\ms2019-04\MS 2019-07-02 10-27-22\069-P2-C3-ZZW-0
Max: 700032
235.1
236.1
m/z

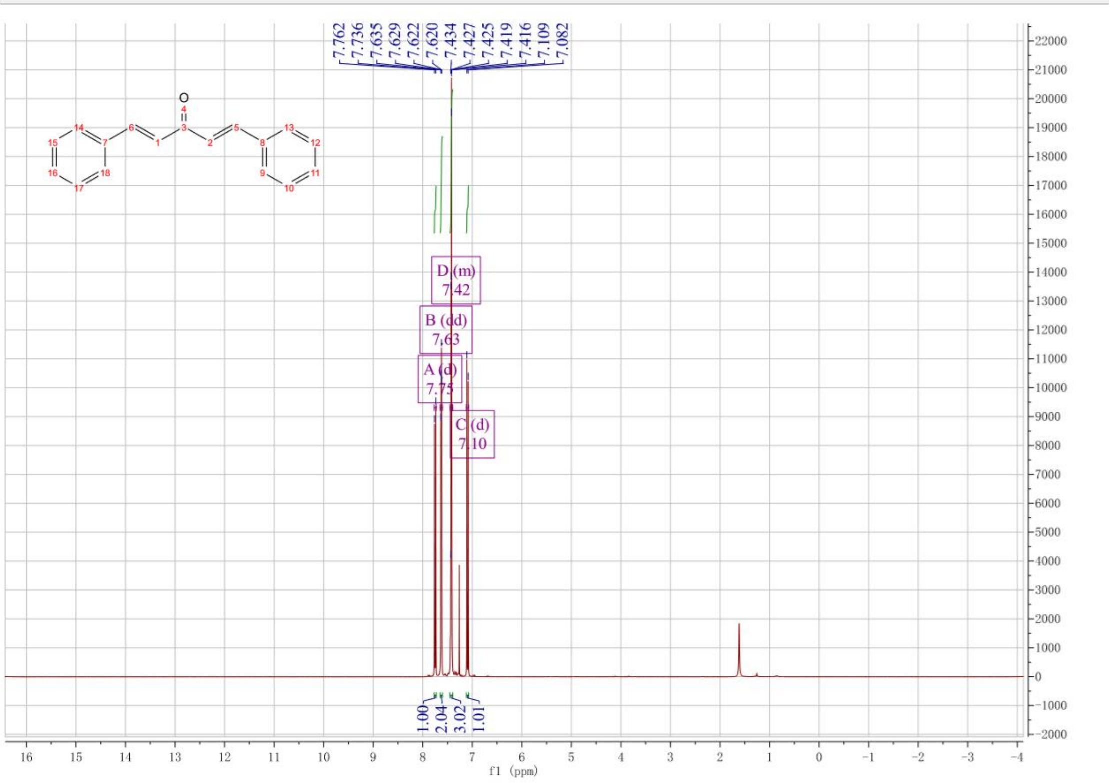
7.762
7.736
7.635
7.629
7.622
7.620
7.434
7.427
7.425
7.419
7.416
7.109
7.082
D (m)
7.42
B (dd)
7.63
A (d)
7.75
C (d)
7.10
1.00
2.04
3.02
1.01
f1 (ppm)

d.

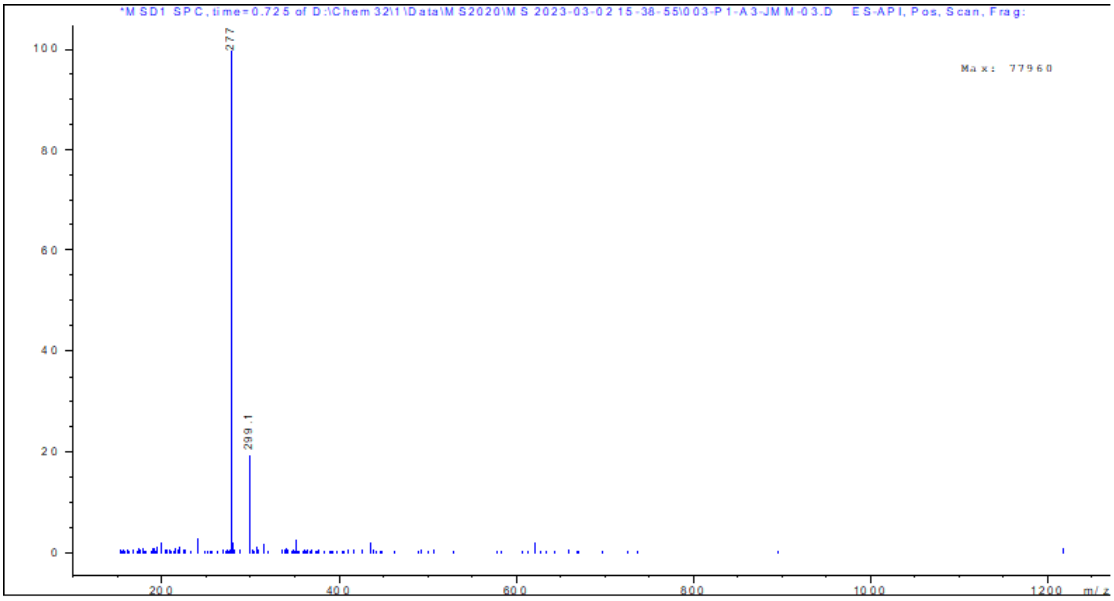

*MSD1 SPC, time=0.725 of D:\Chem32\1\Data\MS2020\MS 2023-03-02 15-38-55\003-P1-A3-JMM-03.D ES-API, Pos, Scan, Frag:
Max: 77960
277
299.1
m/z

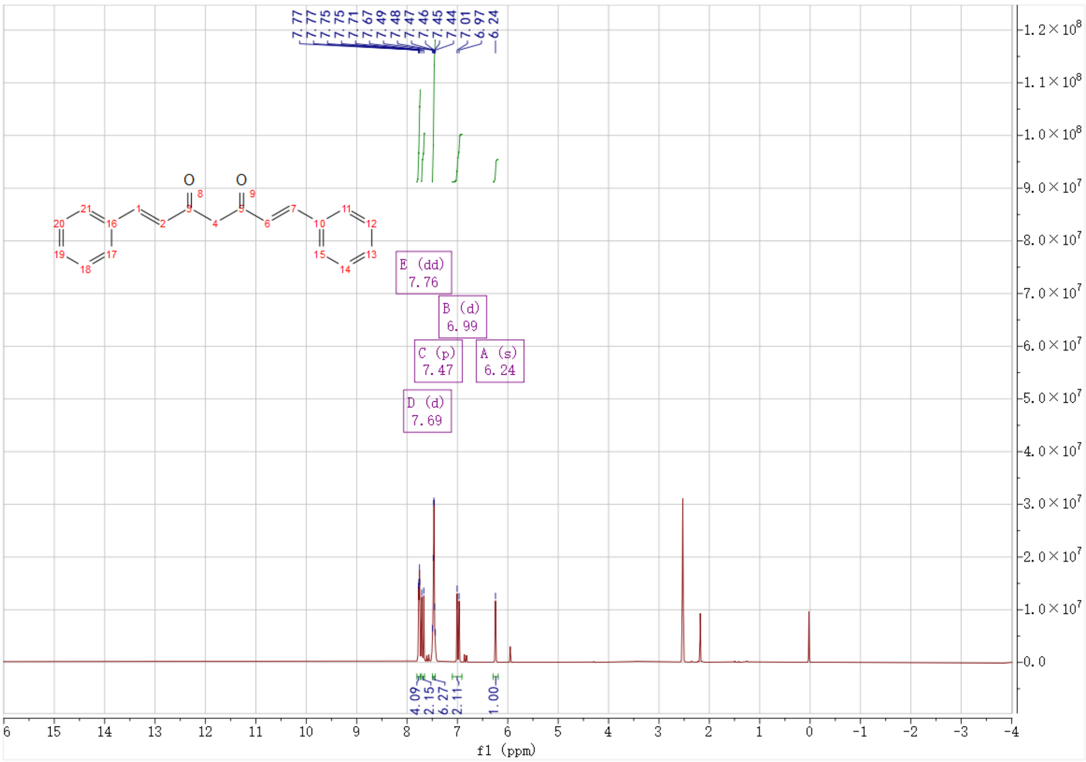

E (dd) 7.76
B (d) 6.99
C (p) 7.47
A (s) 6.24
D (d) 7.69
f1 (ppm)

e.

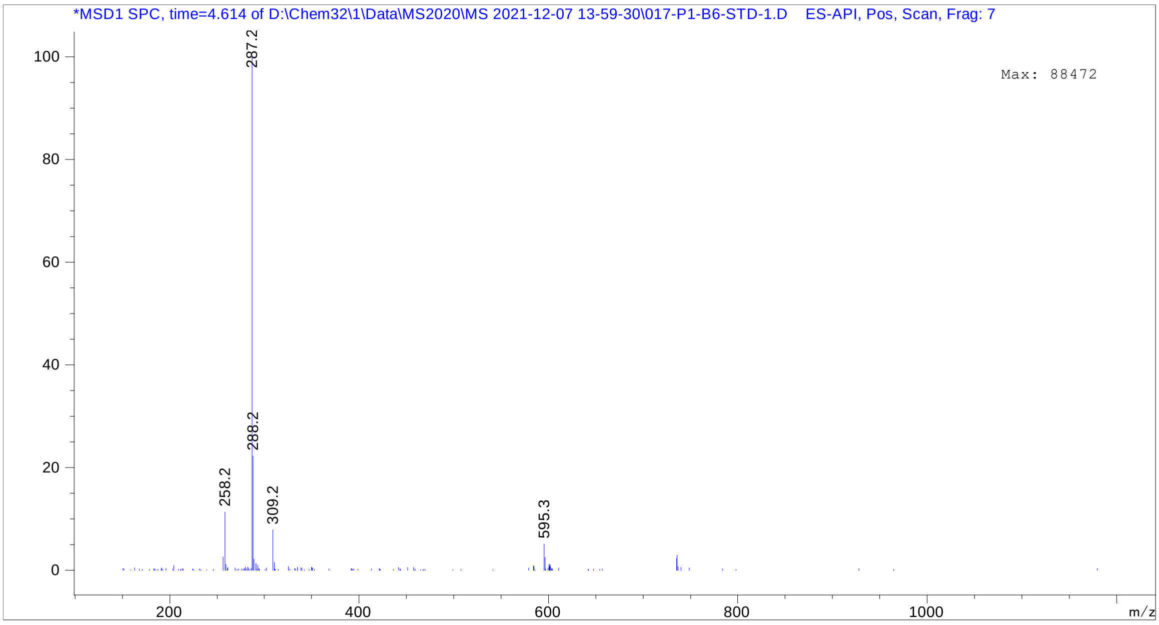
*MSD1 SPC, time=4.614 of D:\Chem32\1\Data\MS2020\MS 2021-12-07 13-59-30\017-P1-B6-STD-1.D ES-API, Pos, Scan, Frag: 7
Max: 88472
287.2
288.2
258.2
309.2
595.3
100
80
60
40
20
0
200
400
600
800
1000
m/z

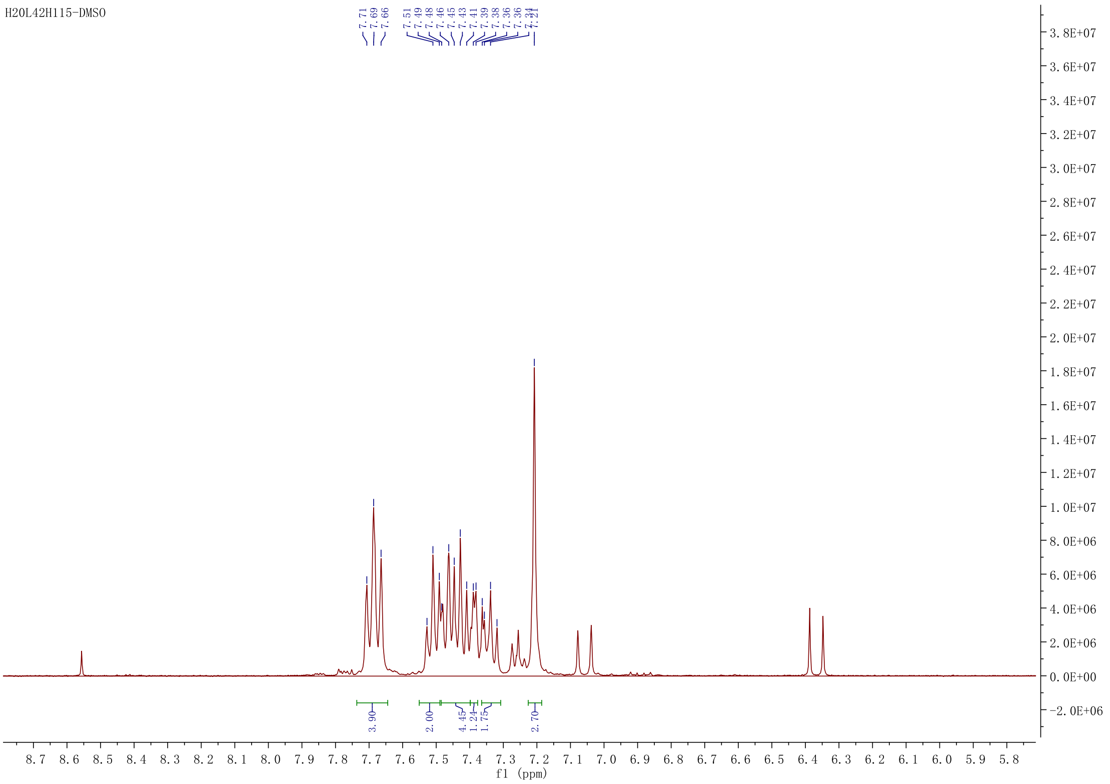
H20L42H115-DMSO
7.71
7.69
7.66
7.51
7.49
7.48
7.46
7.45
7.43
7.41
7.39
7.38
7.36
7.36
7.34
7.21
3.90
2.00
4.45
1.24
1.75
2.70
f1 (ppm)

f.

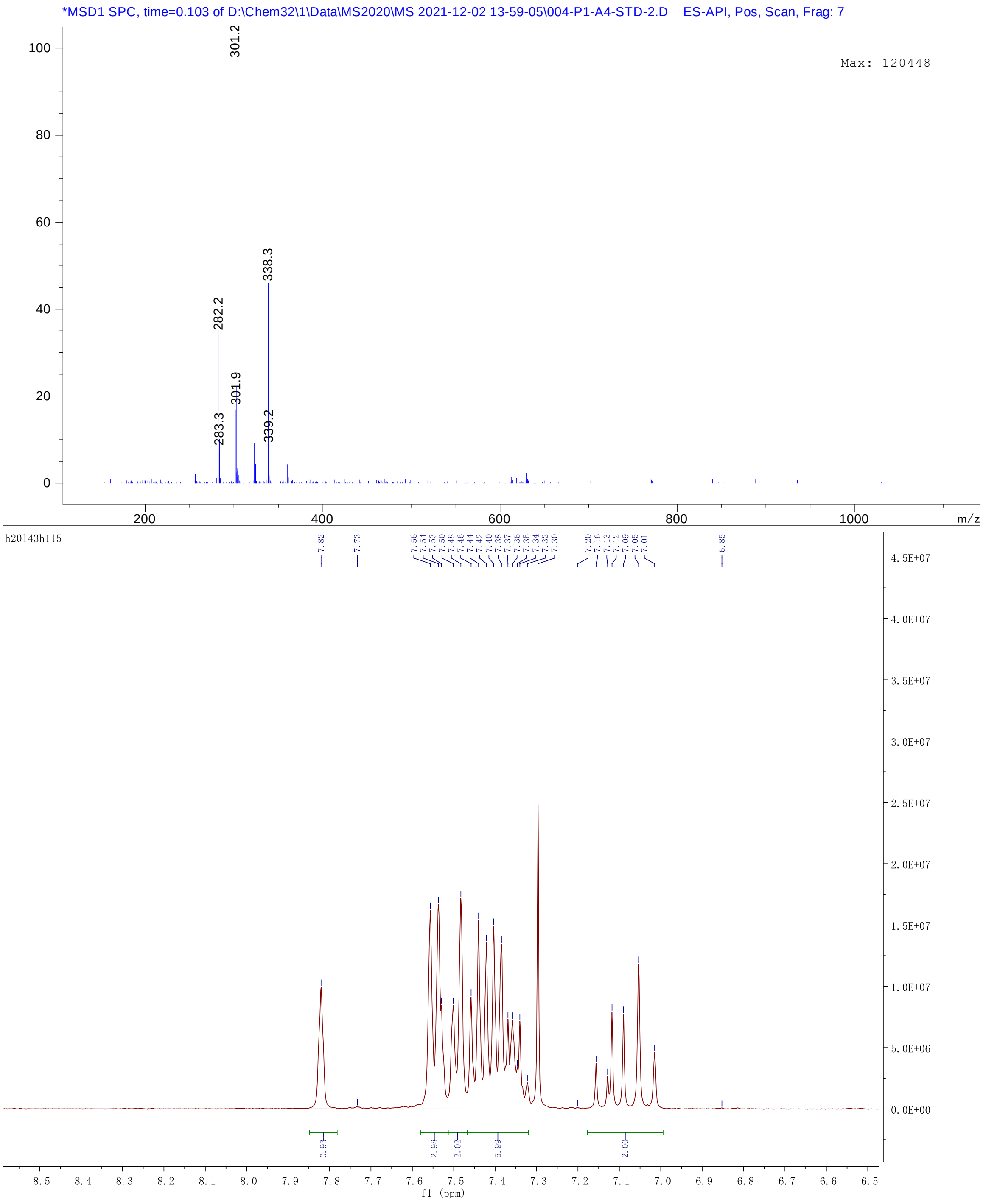
*MSD1 SPC, time=0.103 of D:\Chem32\1\Data\MS2020\MS 2021-12-02 13-59-05\004-P1-A4-STD-2.D ES-API, Pos, Scan, Frag: 7
Max: 120448
301.2
338.3
282.2
301.9
283.3
339.2
m/z
h20143h115
7.82 7.73 7.56 7.54 7.53 7.50 7.48 7.46 7.44 7.42 7.40 7.38 7.37 7.36 7.35 7.34 7.32 7.30 7.20 7.16 7.13 7.12 7.09 7.05 7.01 6.85
0.93 2.98 2.02 5.99 2.00
f1 (ppm)

g.

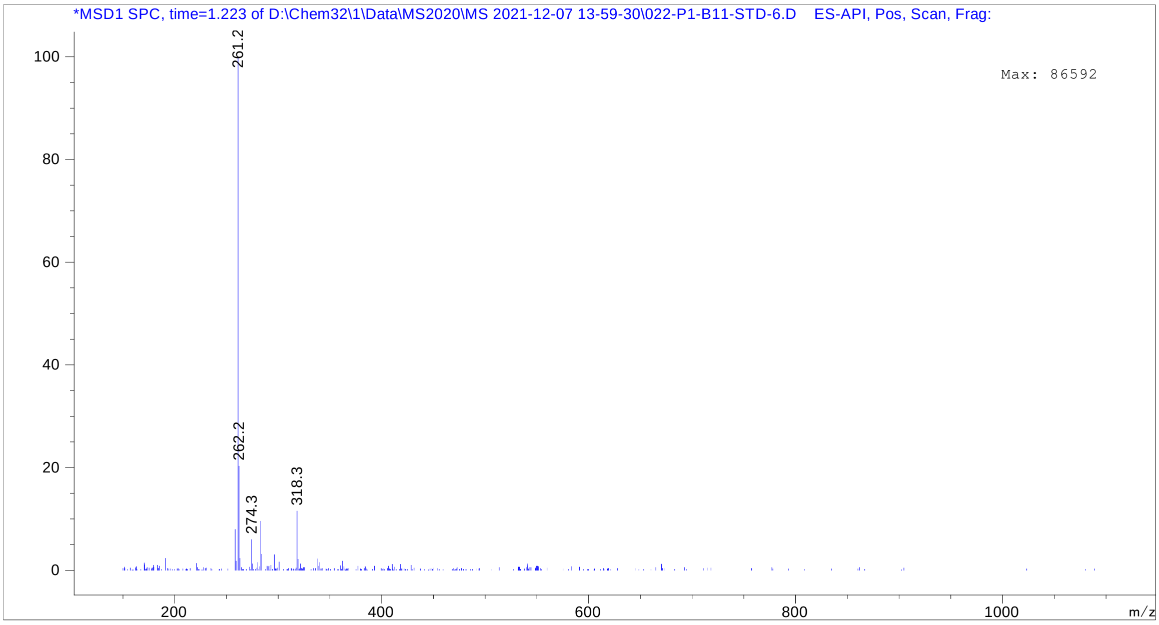
*MSD1 SPC, time=1.223 of D:\Chem32\1\Data\MS2020\MS 2021-12-07 13-59-30\022-P1-B11-STD-6.D ES-API, Pos, Scan, Frag:
Max: 86592
261.2
262.2
274.3
318.3
m/z

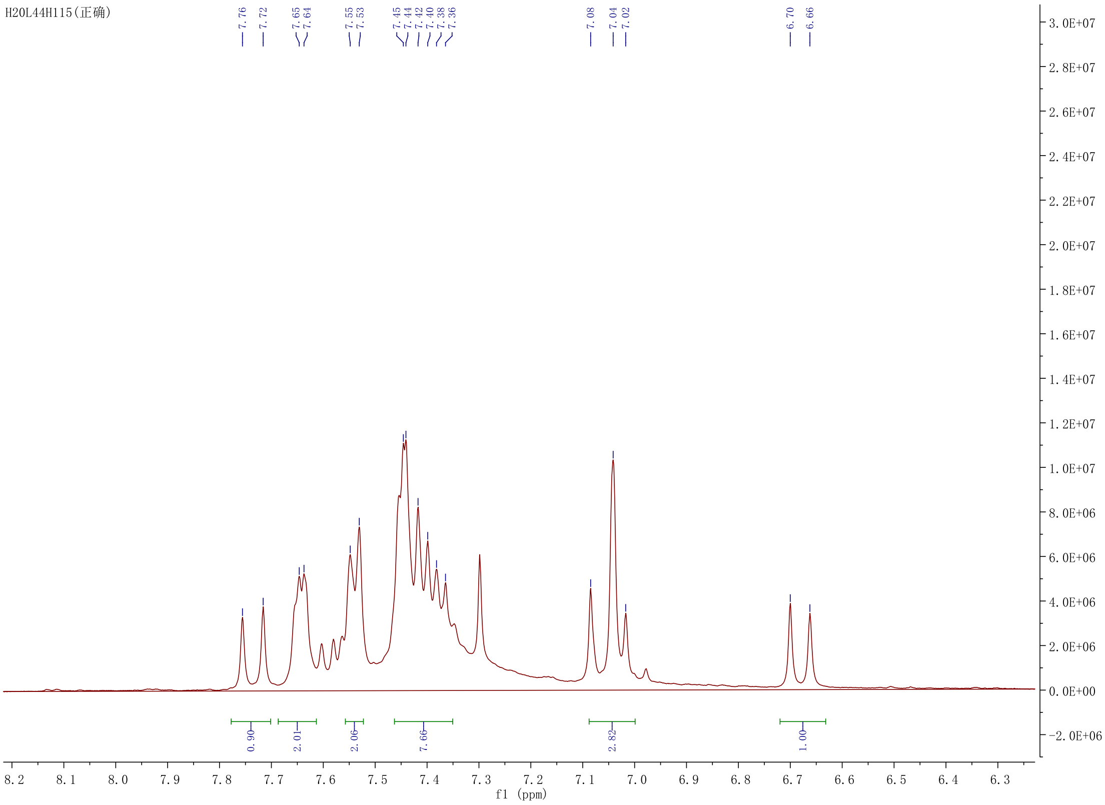
H20L44H115(正确)
f1 (ppm)

1.

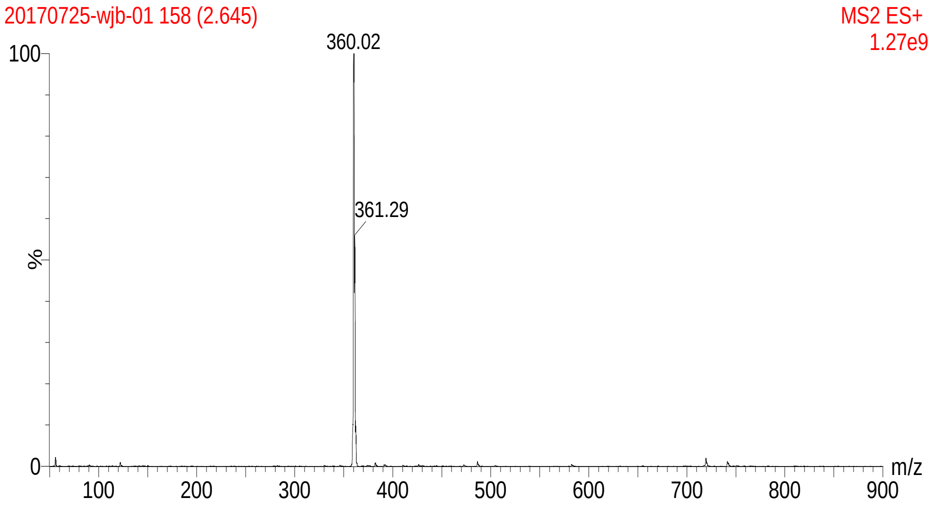

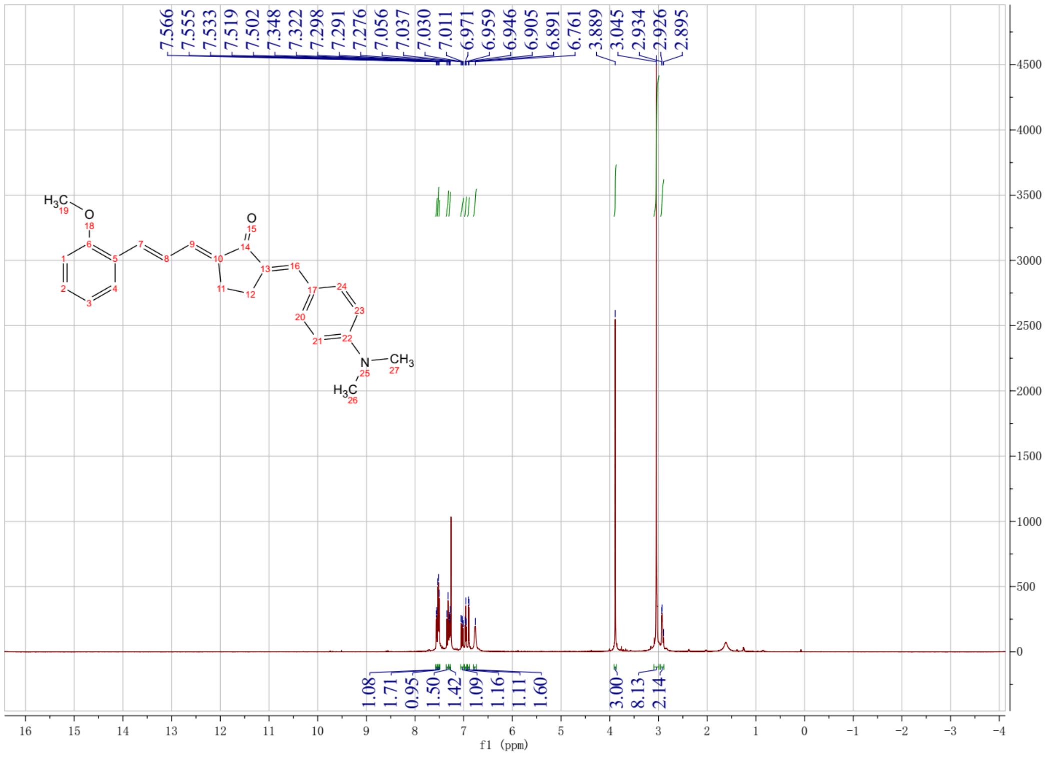

2.

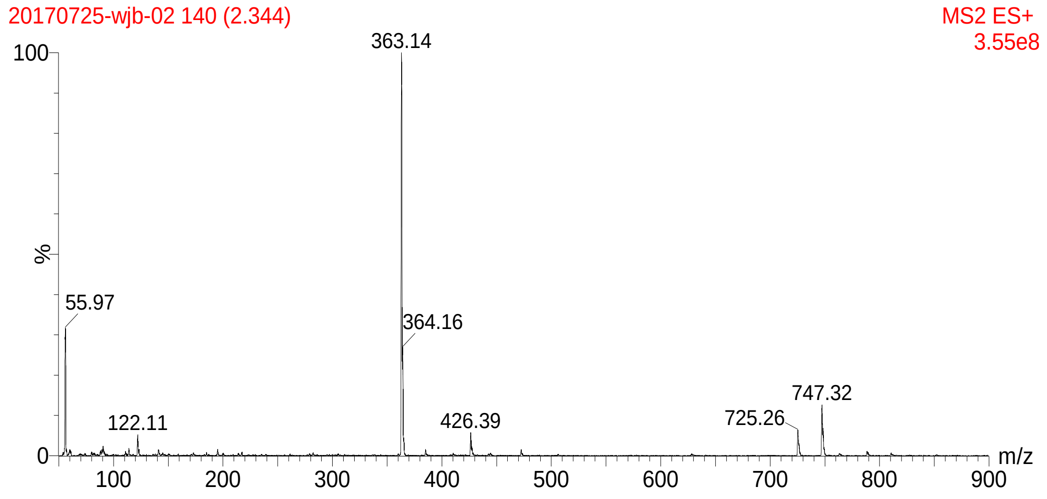

20170725-wjb-02 140 (2.344)
MS2 ES+
3.55e8
100
%
0
363.14
364.16
55.97
122.11
426.39
725.26
747.32
100
200
300
400
500
600
700
800
900
m/z

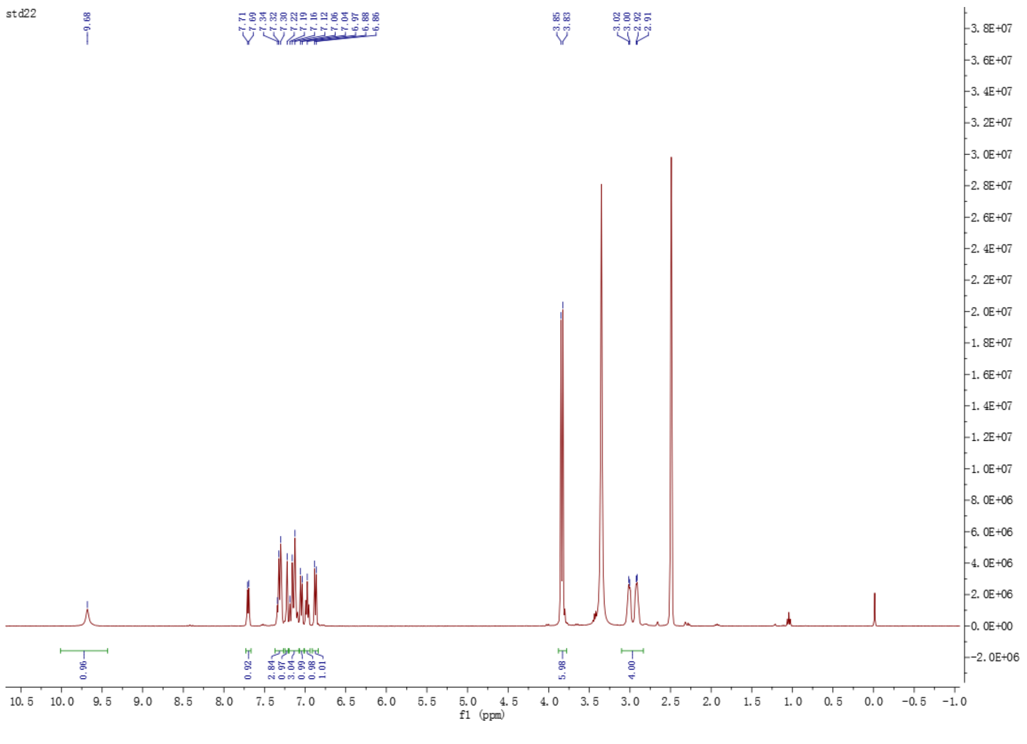

std22
9.68
7.71 7.69 7.34 7.32 7.30 7.22 7.19 7.16 7.12 7.06 7.04 6.97 6.88 6.86
3.85 3.83
3.02 3.00 2.92 2.91
0.96
0.92
2.84 0.97 3.04 0.99 0.98 1.01
5.98
4.00
f1 (ppm)

3.

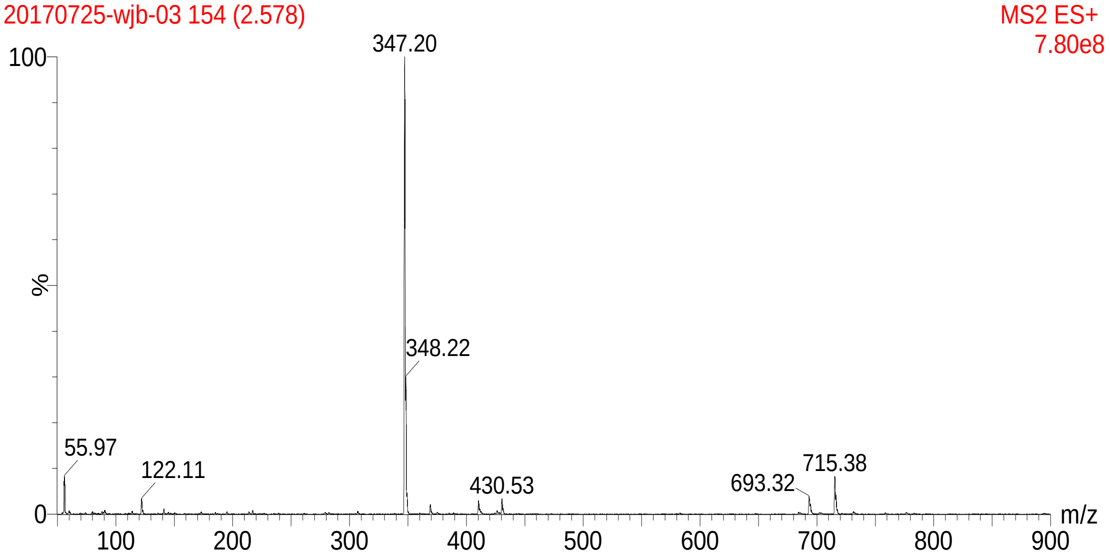

20170725-wjb-03 154 (2.578)
MS2 ES+
7.80e8
347.20
348.22
55.97
122.11
430.53
693.32
715.38
%
m/z

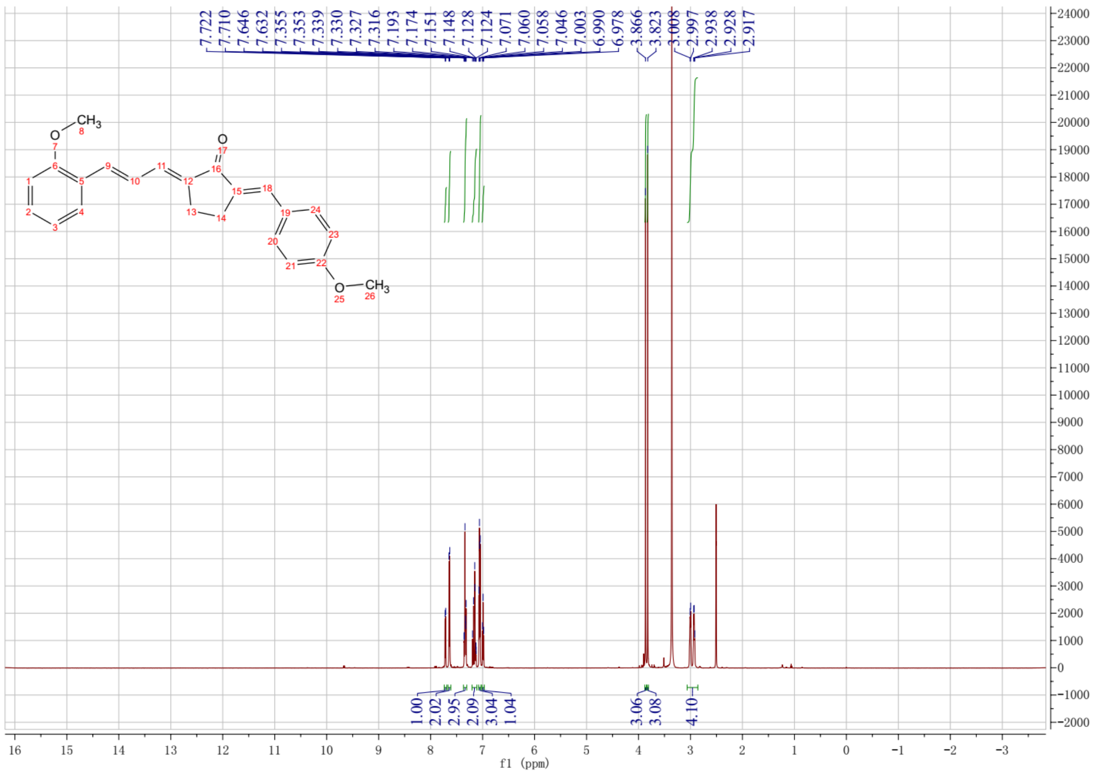

7.722 7.710 7.646 7.632 7.355 7.353 7.339 7.330 7.327 7.316 7.193 7.174 7.151 7.148 7.128 7.124 7.071 7.060 7.058 7.046 7.003 6.990 6.978 3.866 3.823 3.008 2.997 2.938 2.928 2.917
1.00 2.02 2.95 2.09 3.04 1.04 3.06 3.08 4.10
f1 (ppm)

4.

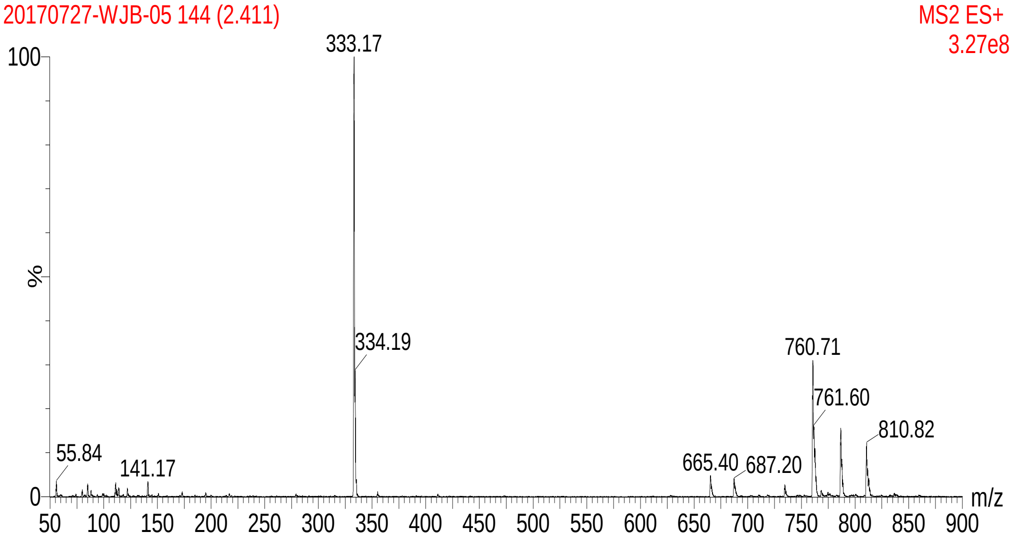
20170727-WJB-05 144 (2.411)
MS2 ES+
3.27e8
333.17
334.19
760.71
761.60
810.82
665.40
687.20
55.84
141.17
%
m/z

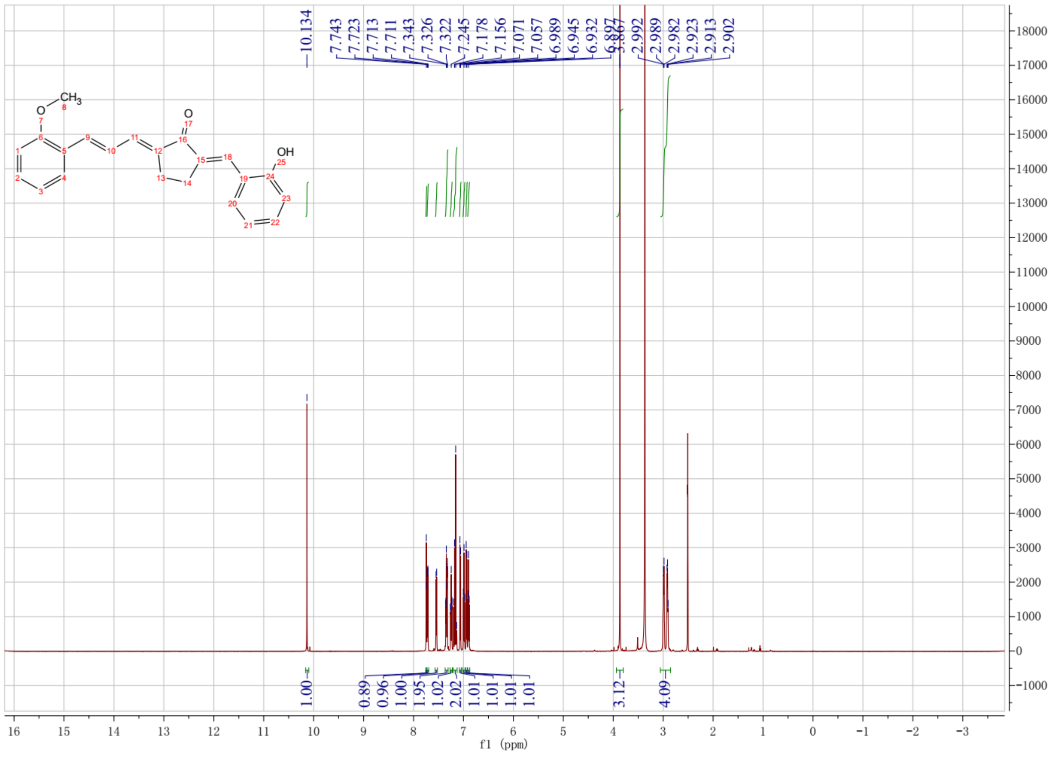
10.134
7.743
7.723
7.713
7.711
7.343
7.326
7.322
7.245
7.178
7.156
7.071
7.057
6.989
6.945
6.932
6.897
3.867
2.992
2.989
2.982
2.923
2.913
2.902
1.00
0.89
0.96
1.00
1.95
1.02
2.02
1.01
1.01
1.01
1.01
3.12
4.09
f1 (ppm)
OH
O
CH3

5.

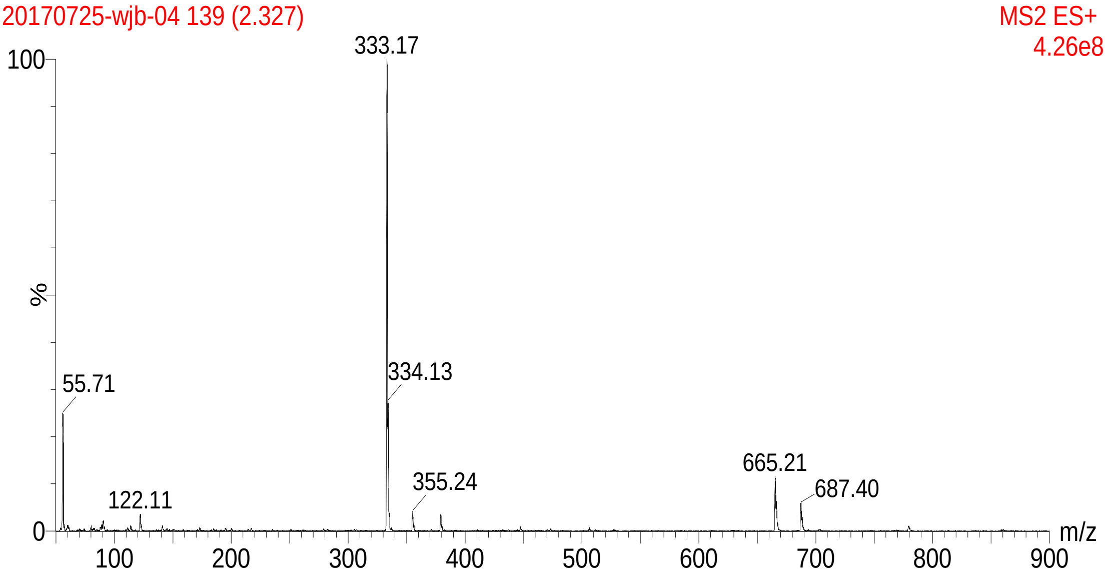

20170725-wjb-04 139 (2.327)
MS2 ES+
4.26e8
100
%
0
333.17
55.71
334.13
122.11
355.24
665.21
687.40
m/z
100
200
300
400
500
600
700
800
900

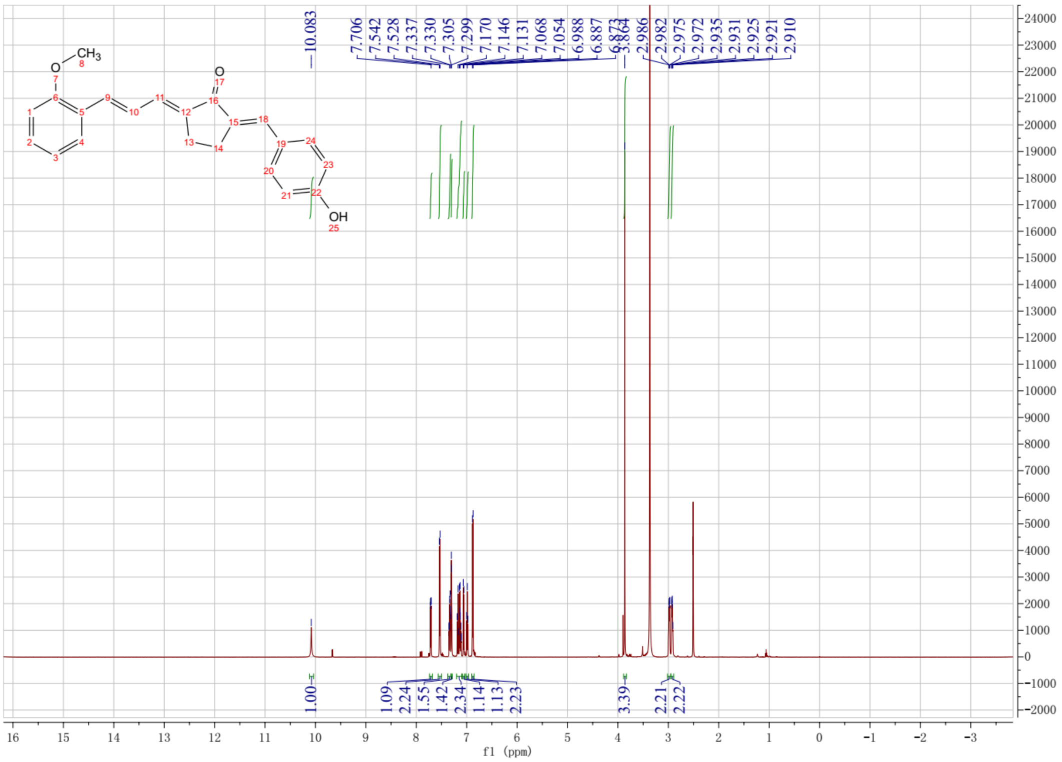

10.083
7.706
7.542
7.528
7.337
7.330
7.305
7.299
7.170
7.146
7.131
7.068
7.054
6.988
6.887
6.873
3.864
2.986
2.982
2.975
2.972
2.935
2.931
2.925
2.921
2.910
1.00
1.09
2.24
1.55
1.42
2.34
1.14
1.13
2.23
3.39
2.21
2.22
f1 (ppm)

6

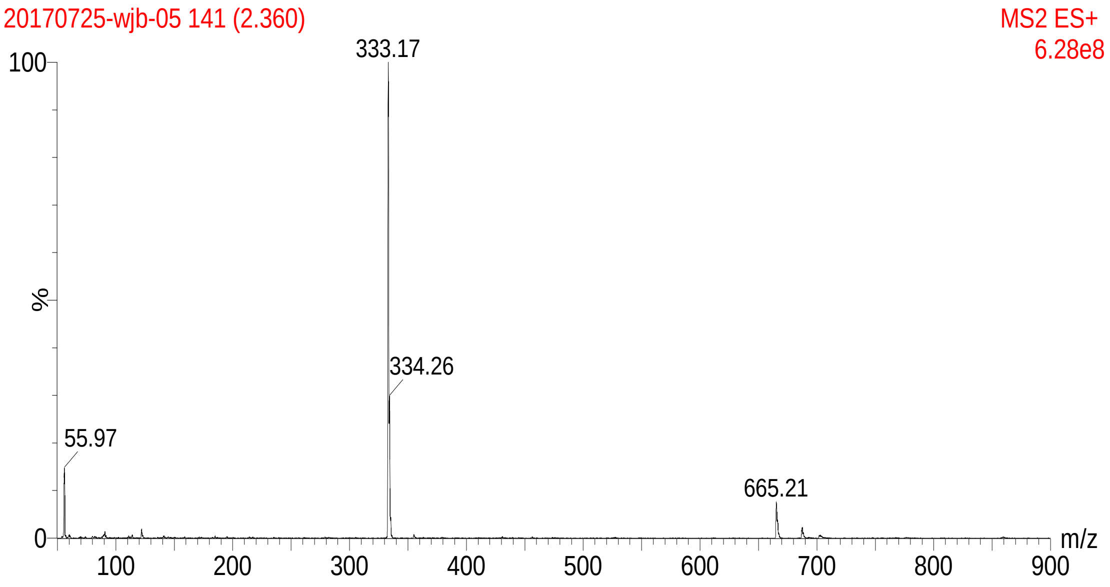
20170725-wjb-05 141 (2.360)
MS2 ES+
6.28e8
333.17
334.26
55.97
665.21
%
m/z

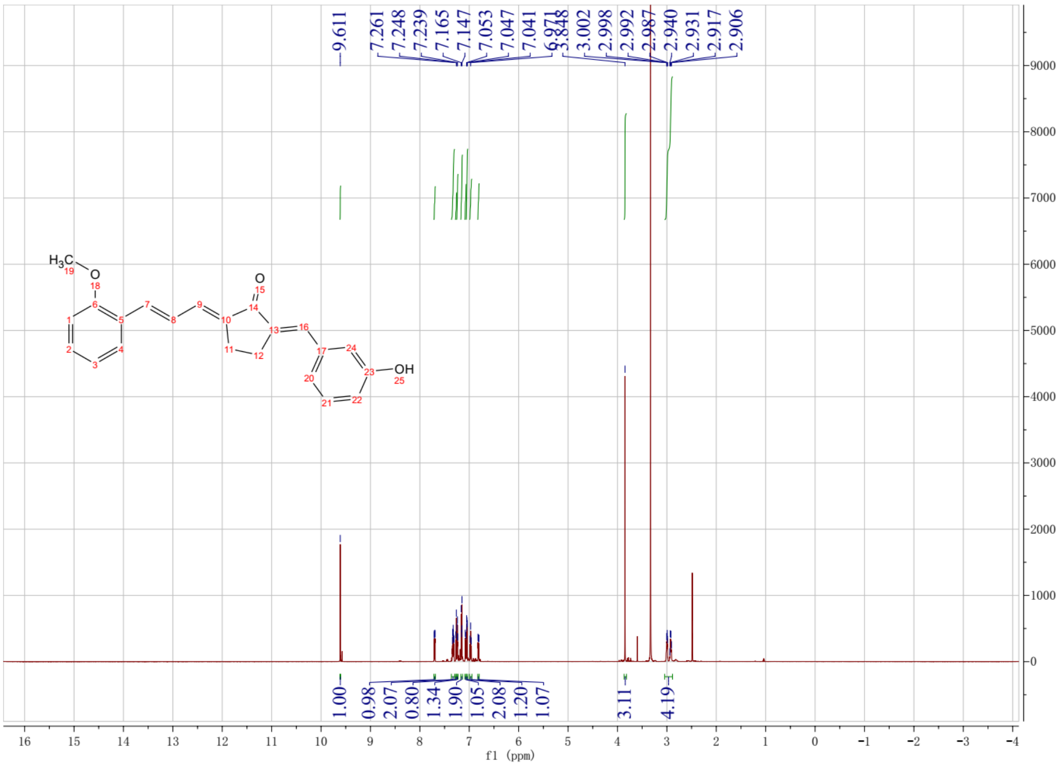
f1 (ppm)

7

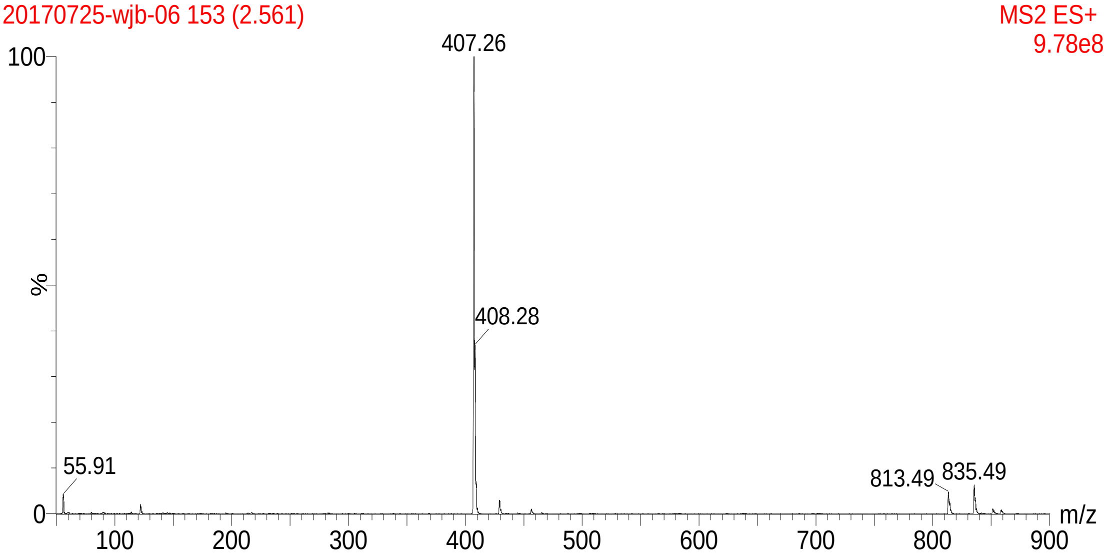
20170725-wjb-06 153 (2.561)
MS2 ES+
9.78e8
407.26
408.28
55.91
813.49
835.49
%
m/z
100
0
100
200
300
400
500
600
700
800
900

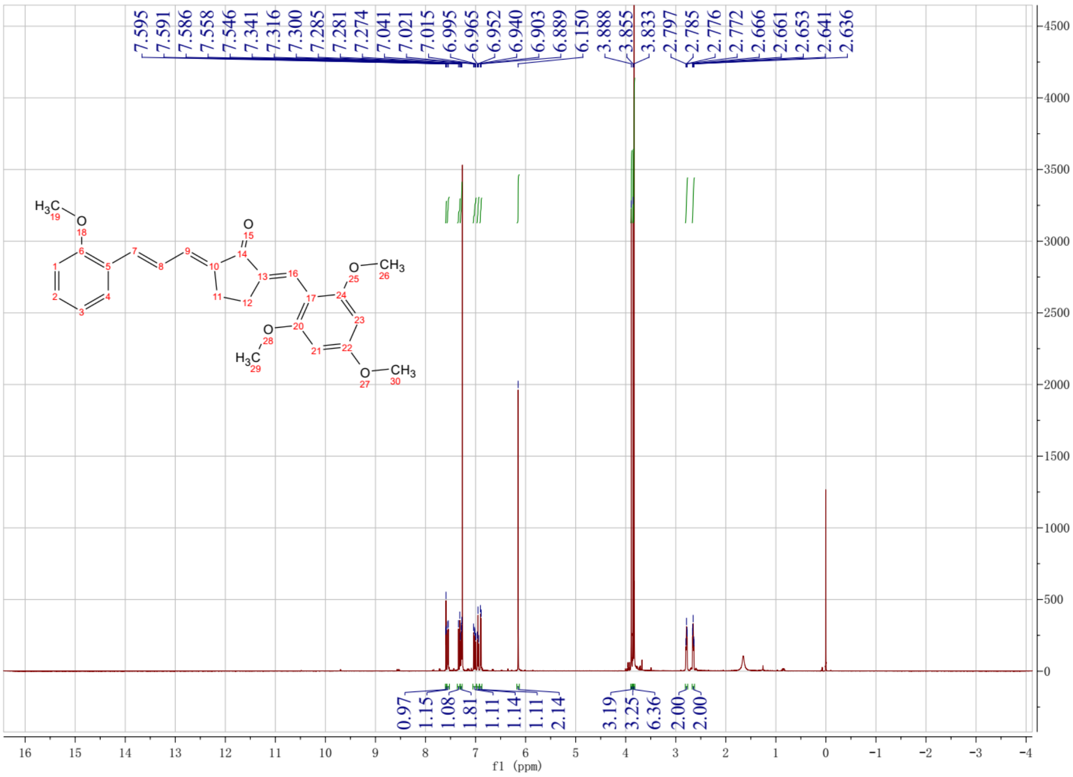
7.595
7.591
7.586
7.558
7.546
7.341
7.316
7.300
7.285
7.281
7.274
7.041
7.021
7.015
6.995
6.965
6.952
6.940
6.903
6.889
6.150
3.888
3.855
3.833
2.797
2.785
2.776
2.772
2.666
2.661
2.653
2.641
2.636
0.97
1.15
1.08
1.81
1.11
1.14
1.11
2.14
3.19
3.25
6.36
2.00
2.00
f1 (ppm)

8

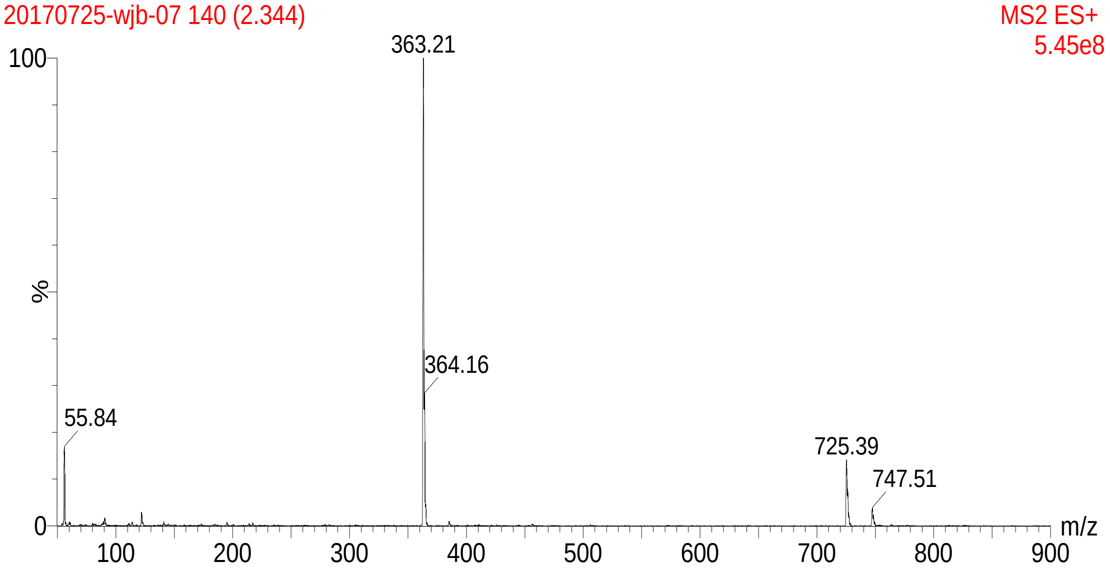
20170725-wjb-07 140 (2.344)
MS2 ES+
5.45e8
363.21
364.16
55.84
725.39
747.51
%
m/z

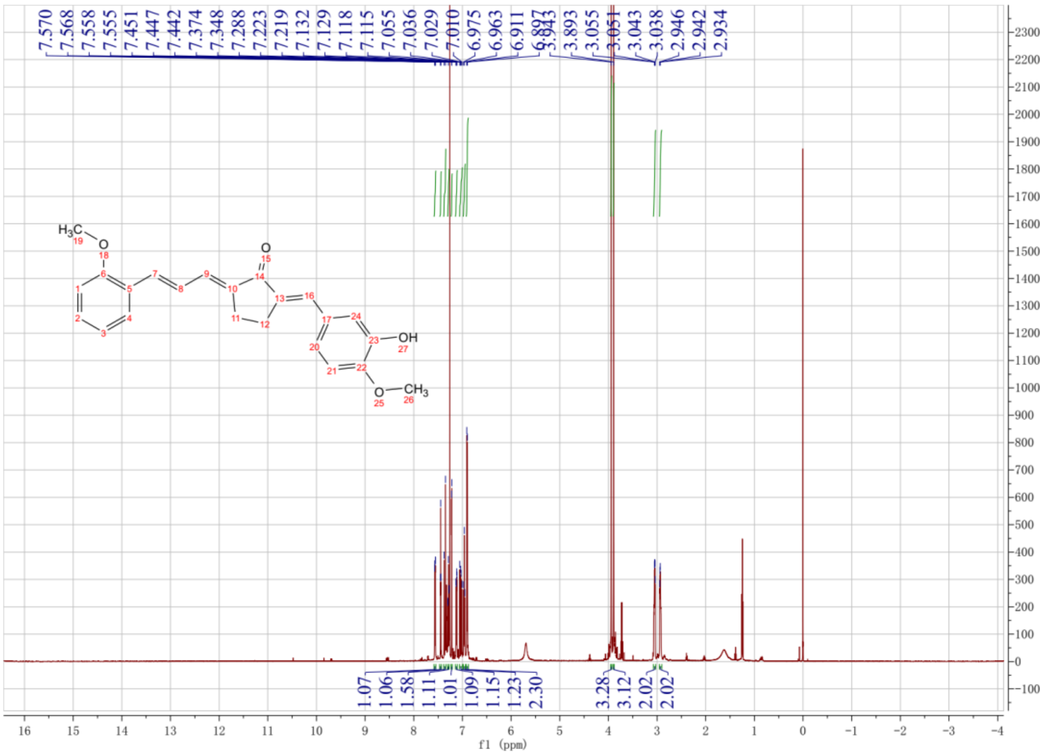
7.570 7.568 7.558 7.555 7.451 7.447 7.442 7.442 7.374 7.348 7.288 7.223 7.219 7.132 7.129 7.118 7.115 7.055 7.036 7.029 7.010 6.975 6.963 6.911 6.897 3.913 3.893 3.055 3.051 3.043 3.038 2.946 2.942 2.934
1.07 1.06 1.58 1.11 1.01 1.09 1.15 1.23 2.30 3.28 3.12 2.02 2.02
f1 (ppm)
H3C
O
OH
O
CH3

9

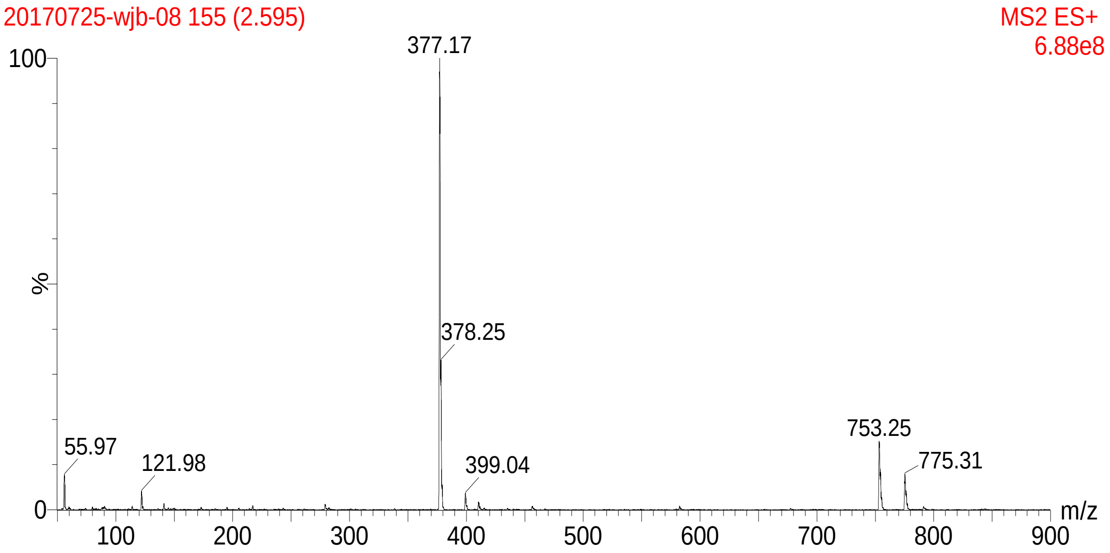
20170725-wjb-08 155 (2.595)
MS2 ES+
6.88e8
377.17
378.25
55.97
121.98
399.04
753.25
775.31
100
%
0
m/z
100
200
300
400
500
600
700
800
900

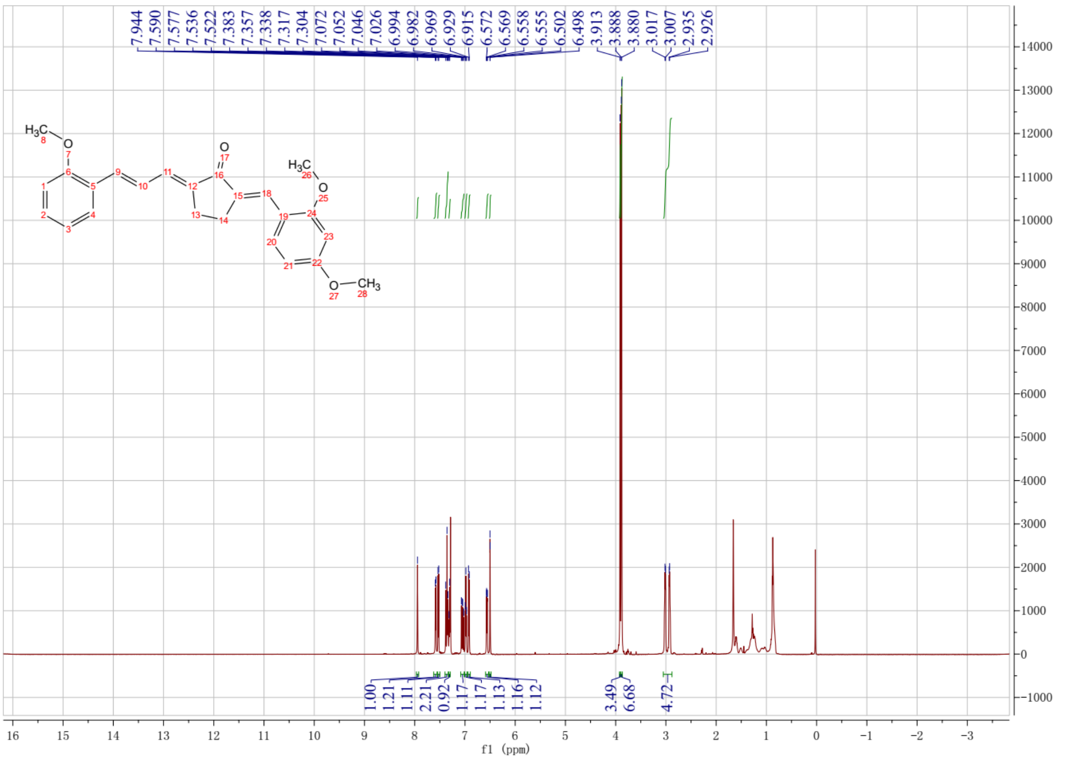
7.944
7.590
7.577
7.536
7.522
7.383
7.357
7.338
7.317
7.304
7.072
7.052
7.046
7.026
6.994
6.982
6.969
6.929
6.915
6.572
6.569
6.558
6.555
6.502
6.498
3.913
3.888
3.880
3.017
3.007
2.935
2.926
1.00
1.21
1.11
2.21
0.92
1.17
1.17
1.13
1.16
1.12
3.49
6.68
4.72
f1 (ppm)

10

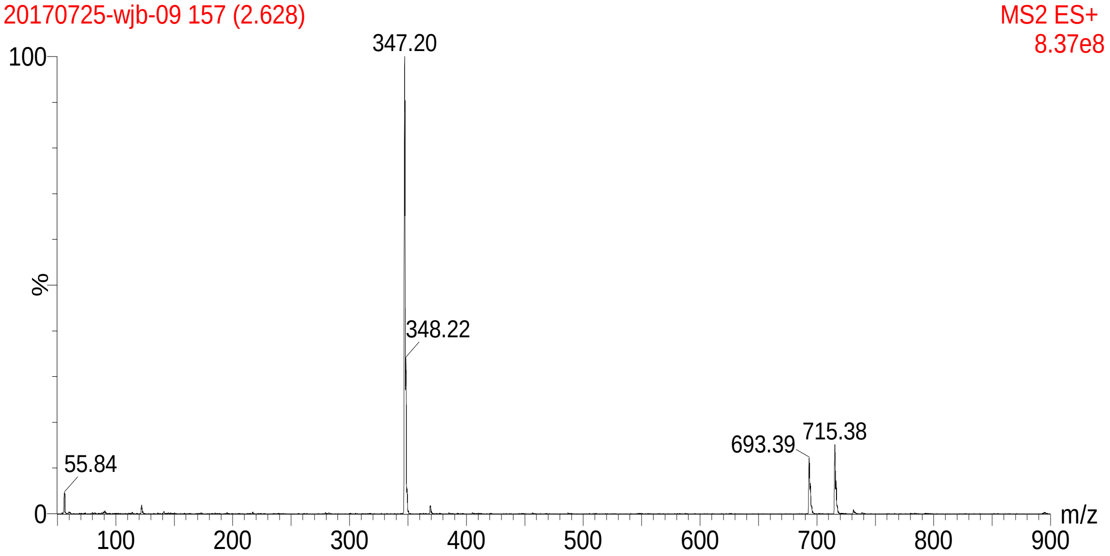
20170725-wjb-09 157 (2.628)
MS2 ES+
8.37e8
347.20
348.22
693.39
715.38
55.84
100
%
0
100
200
300
400
500
600
700
800
900
m/z

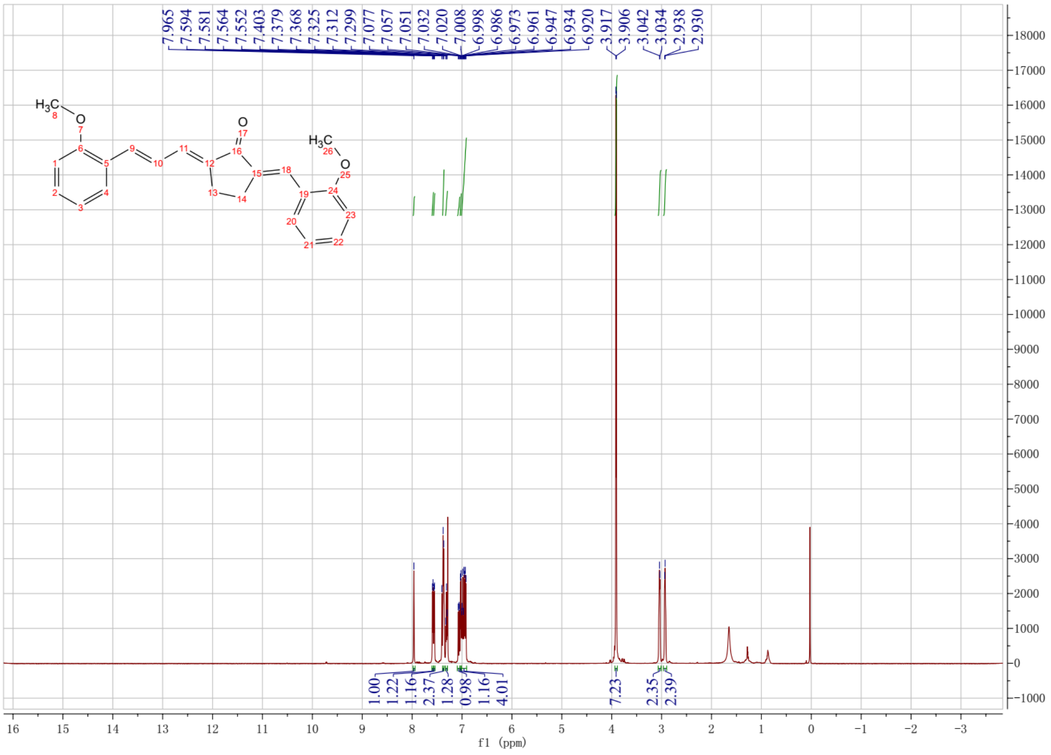
7.965
7.594
7.581
7.564
7.552
7.403
7.379
7.368
7.325
7.312
7.299
7.077
7.057
7.051
7.032
7.020
7.008
6.998
6.986
6.973
6.961
6.947
6.934
6.920
3.917
3.906
3.042
3.034
2.938
2.930
1.00
1.22
1.16
2.37
1.28
0.98
1.16
4.01
7.23
2.35
2.39
f1 (ppm)

11

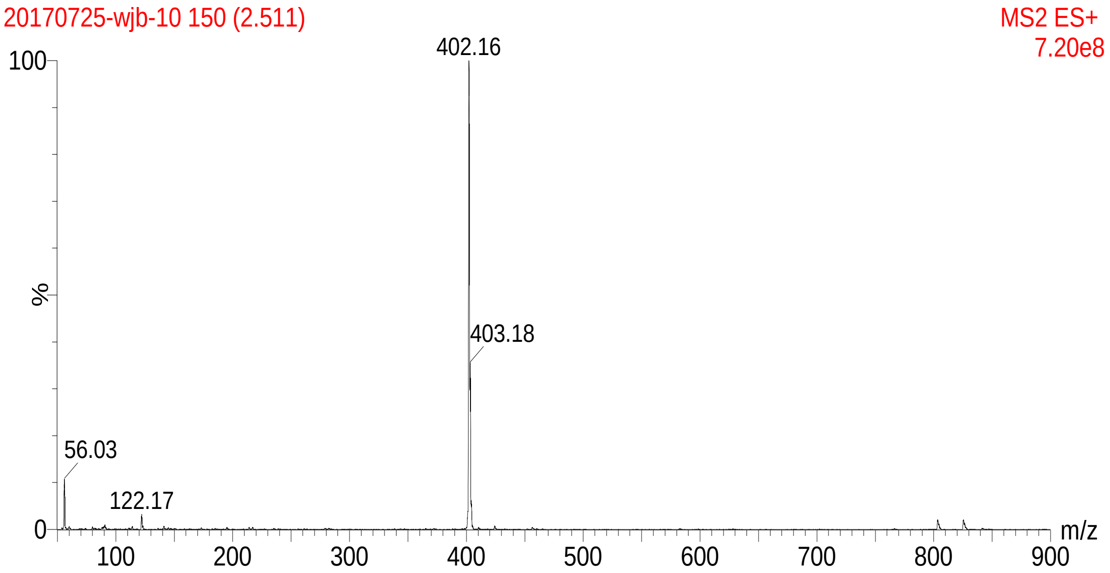

20170725-wjb-10 150 (2.511)
MS2 ES+
7.20e8
402.16
403.18
56.03
122.17
%
m/z

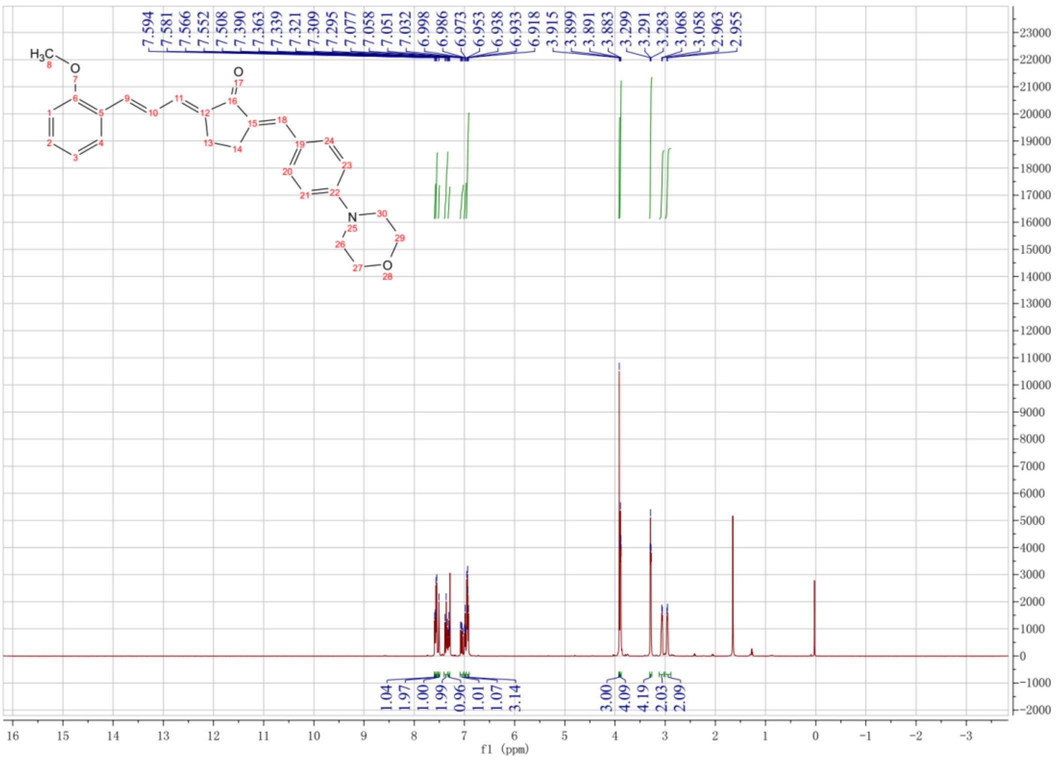

f1 (ppm)

12

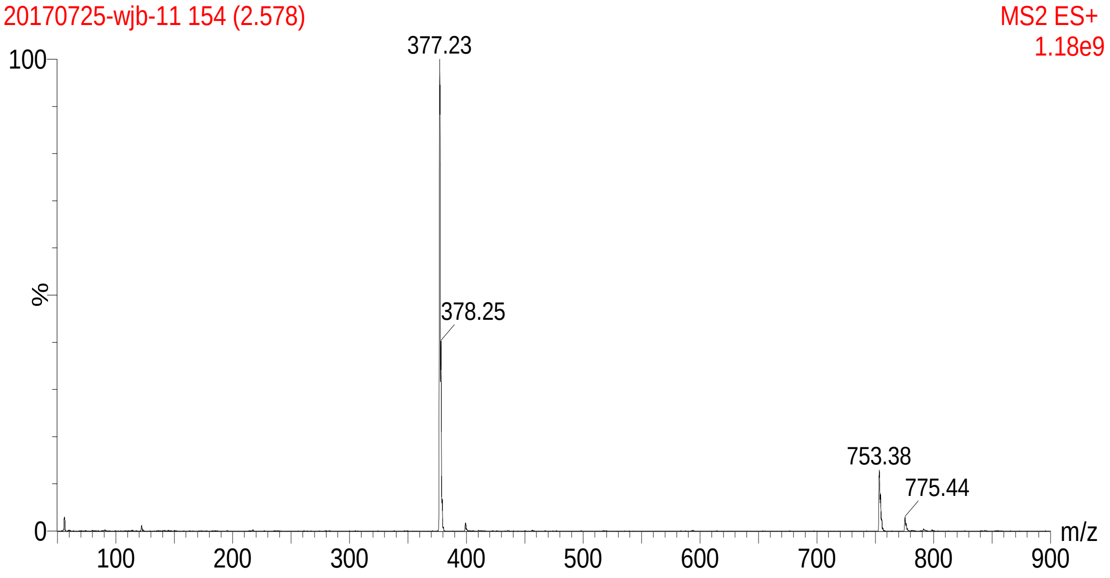
20170725-wjb-11 154 (2.578)
MS2 ES+
1.18e9
377.23
378.25
753.38
775.44
100
%
0
m/z
100
200
300
400
500
600
700
800
900

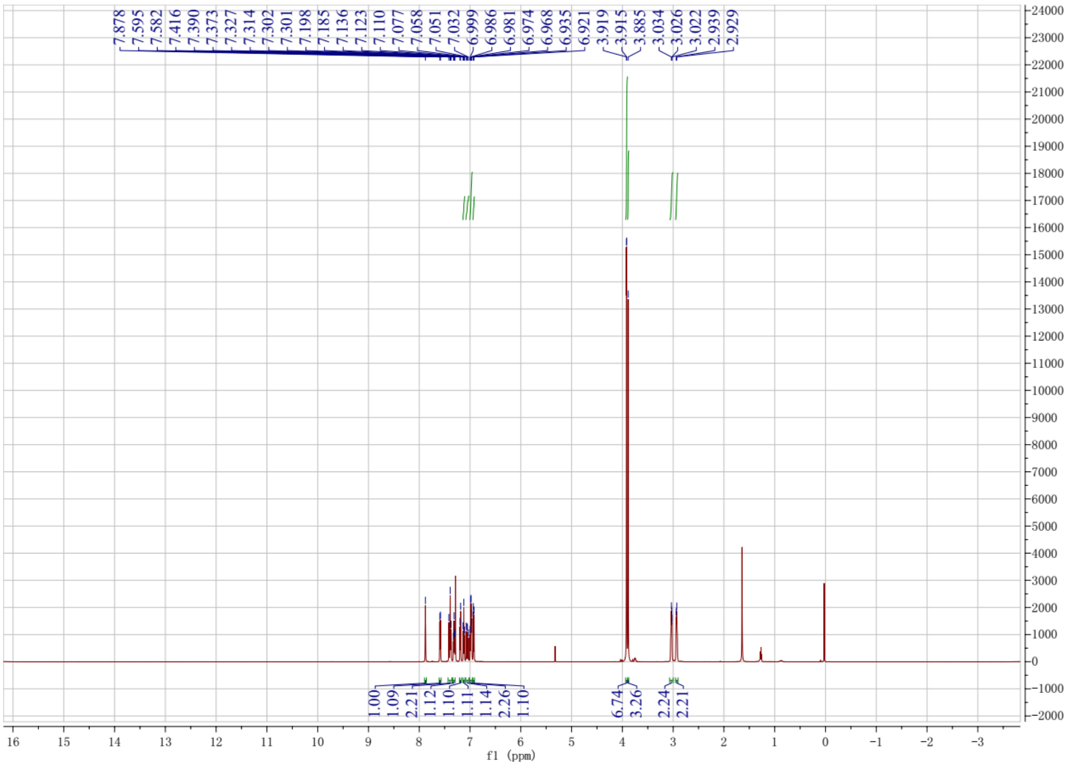
7.878
7.595
7.582
7.416
7.390
7.373
7.327
7.314
7.302
7.301
7.198
7.185
7.136
7.123
7.110
7.077
7.058
7.051
7.032
6.999
6.986
6.981
6.974
6.968
6.935
6.921
3.919
3.915
3.885
3.034
3.026
3.022
2.939
2.929
1.00
1.09
2.21
1.12
1.10
1.11
1.14
2.26
1.10
6.74
3.26
2.24
2.21
f1 (ppm)

13

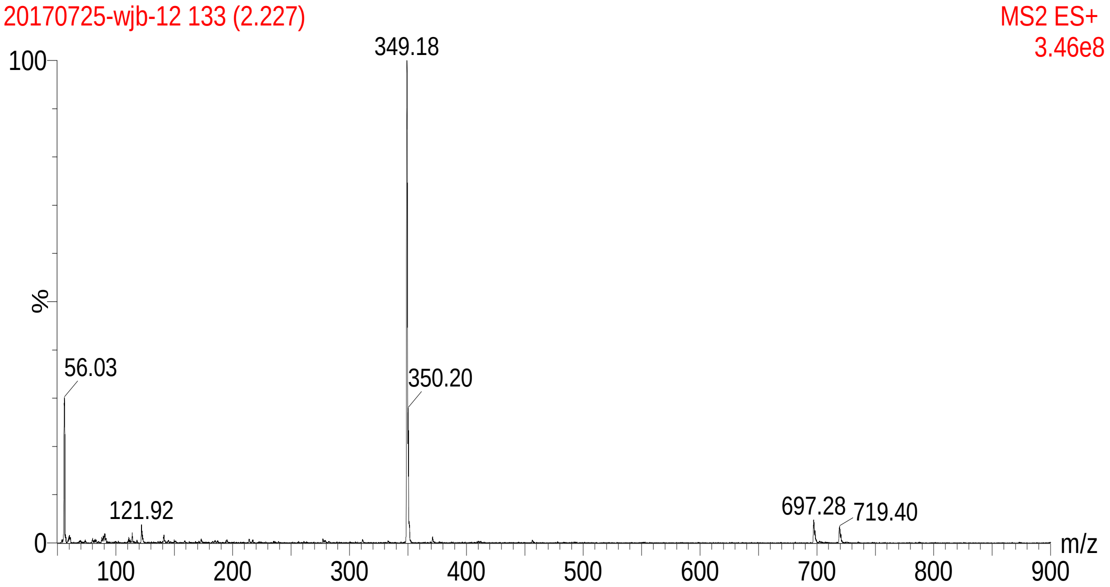

20170725-wjb-12 133 (2.227)
MS2 ES+
3.46e8
349.18
56.03
350.20
121.92
697.28
719.40
%
m/z

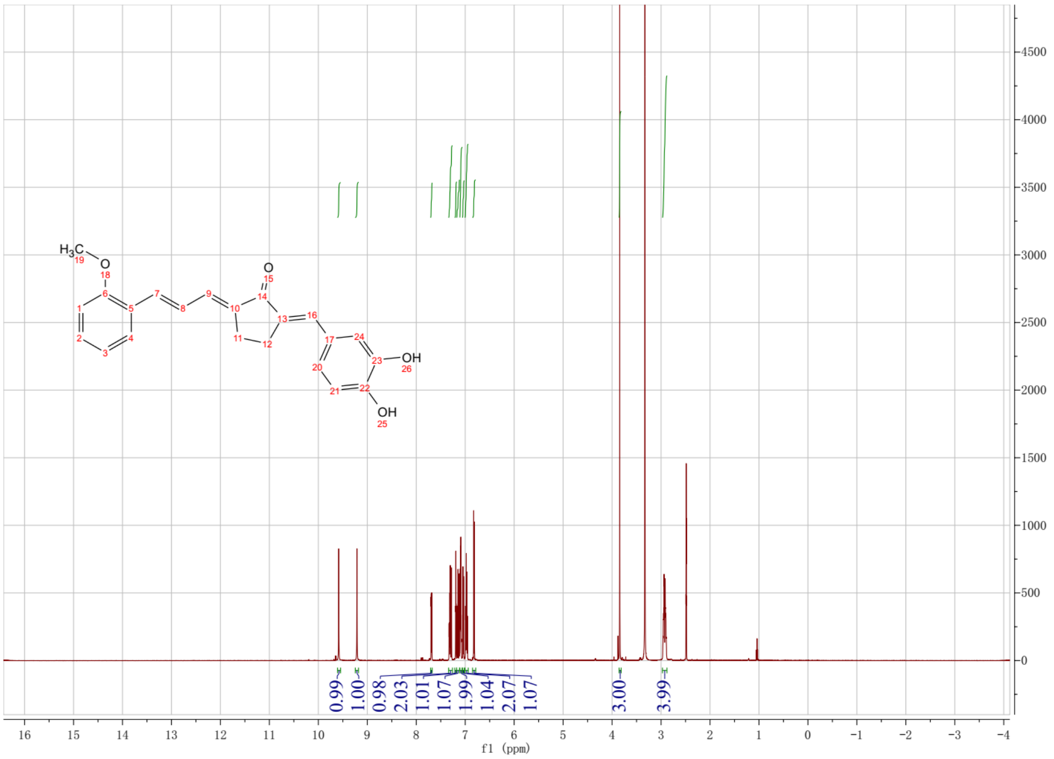

f1 (ppm)

14

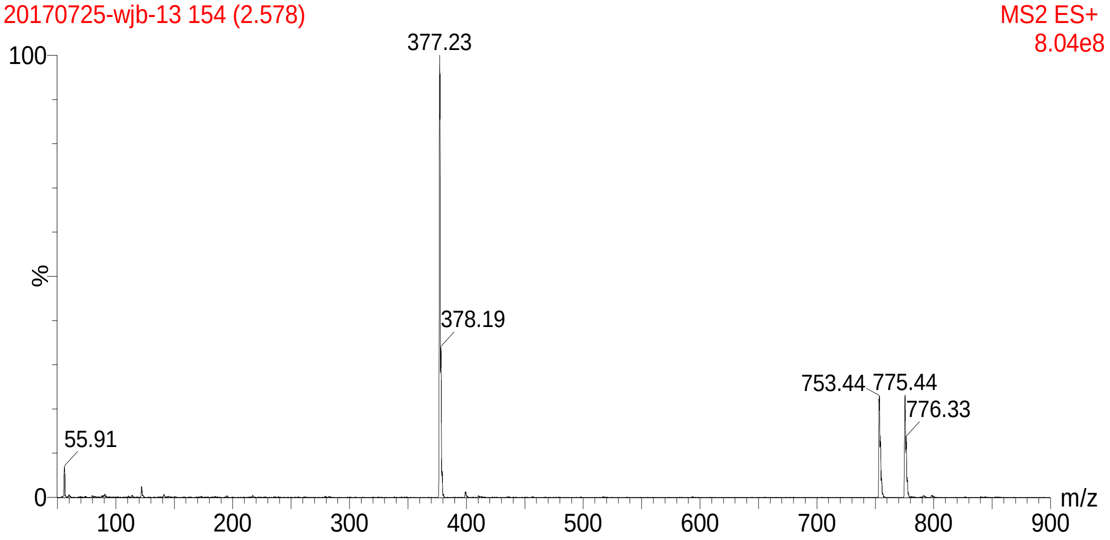

20170725-wjb-13 154 (2.578)
MS2 ES+
8.04e8
377.23
378.19
753.44
775.44
776.33
55.91
%
m/z

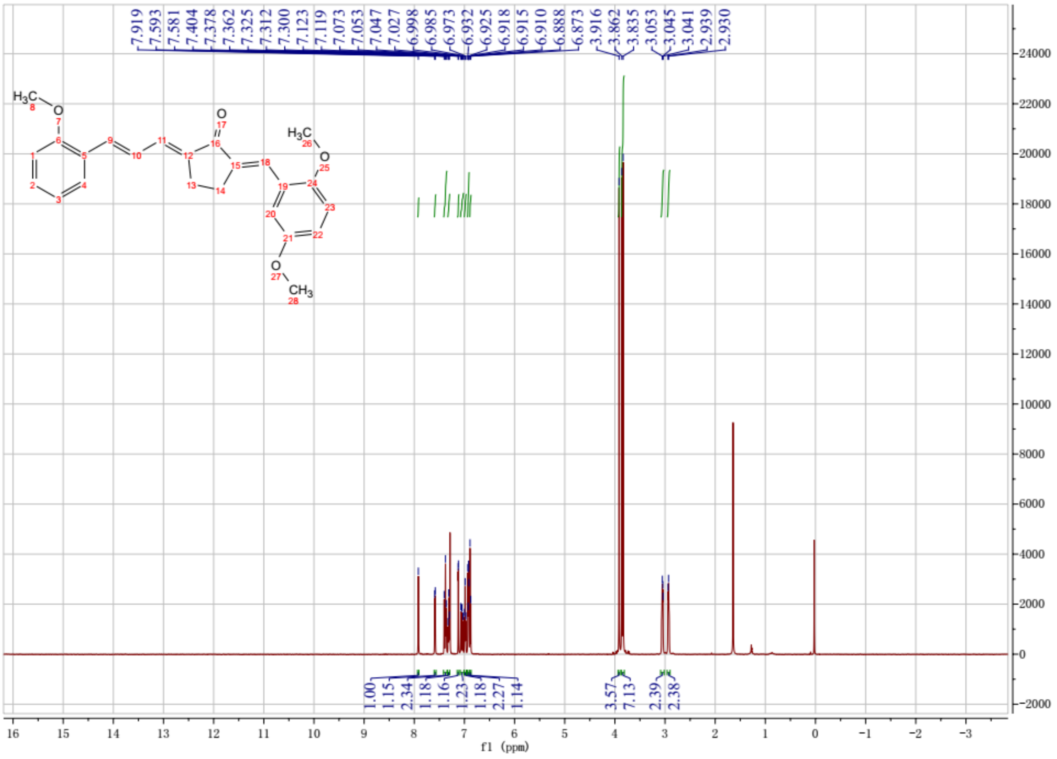

f1 (ppm)

15

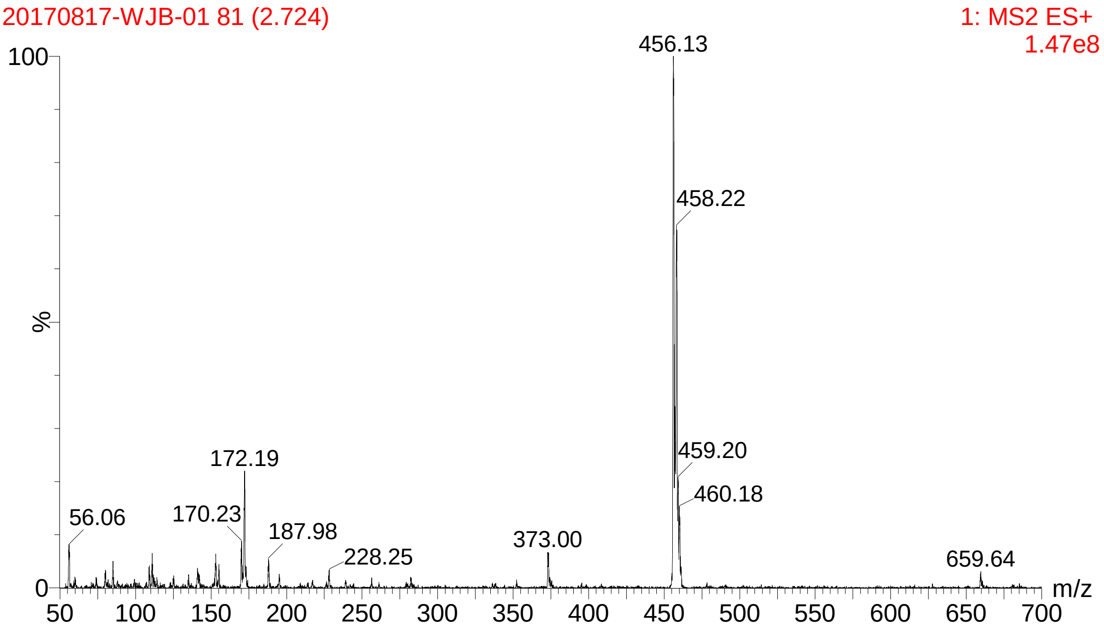
20170817-WJB-01 81 (2.724)
1: MS2 ES+
1.47e8
456.13
458.22
459.20
460.18
172.19
170.23
187.98
56.06
228.25
373.00
659.64
%
m/z

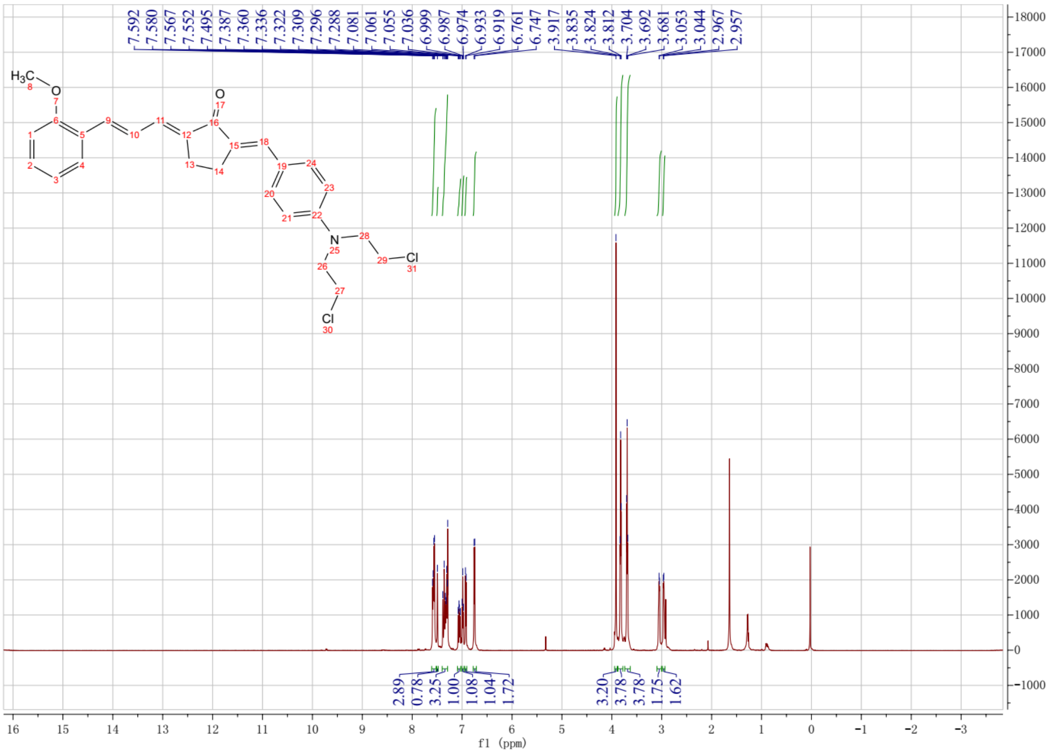
f1 (ppm)

16

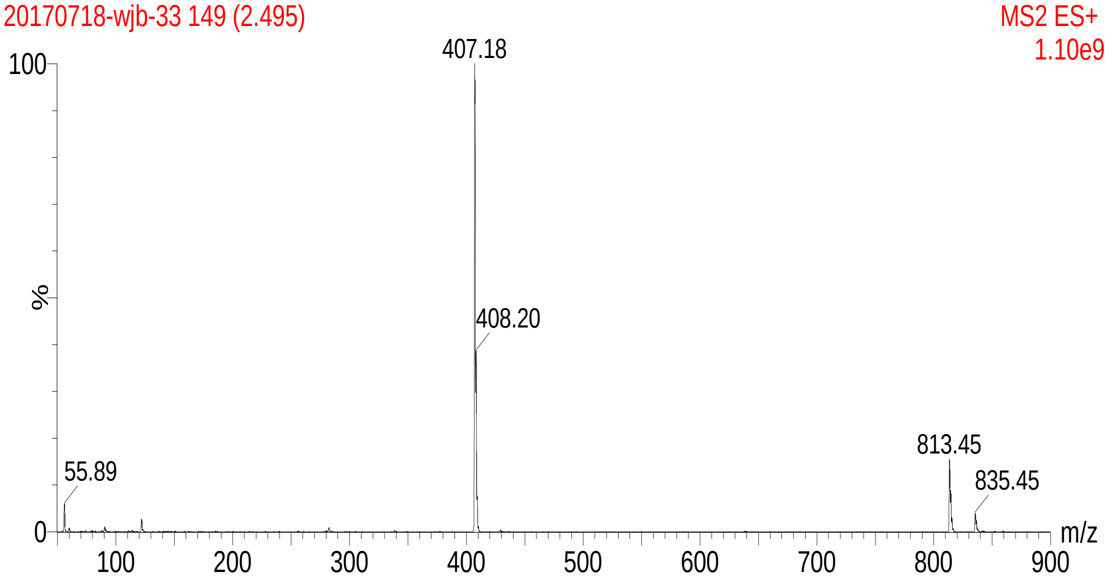

20170718-wjb-33 149 (2.495)
MS2 ES+
1.10e9
407.18
408.20
55.89
813.45
835.45
%
m/z

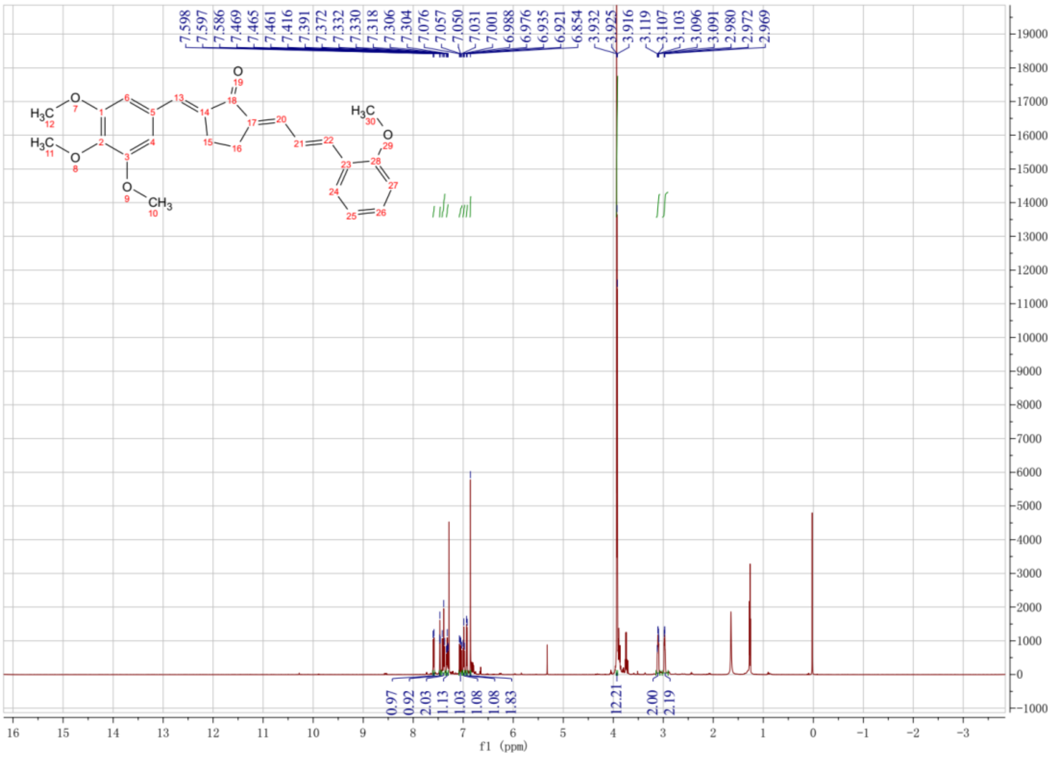

7.598 7.597 7.586 7.469 7.465 7.461 7.416 7.391 7.372 7.332 7.330 7.318 7.306 7.304 7.076 7.057 7.050 7.031 7.001 6.988 6.976 6.935 6.921 6.854 3.932 3.925 3.916 3.119 3.107 3.103 3.096 3.091 2.980 2.972 2.969
0.97 0.92 2.03 1.13 1.03 1.08 1.08 1.83 12.21 2.00 2.19
f1 (ppm)

17

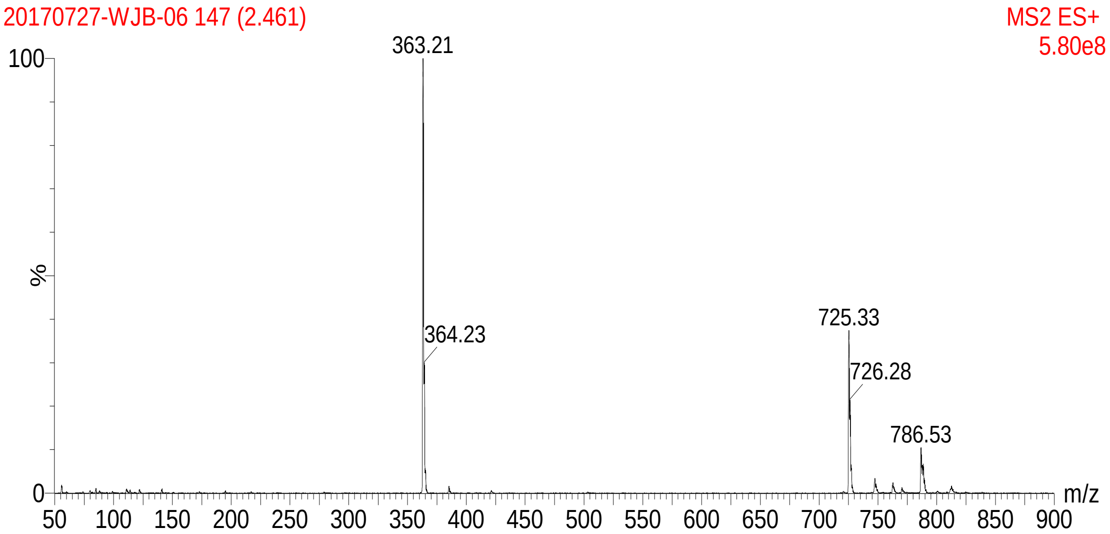

20170727-WJB-06 147 (2.461)
MS2 ES+
5.80e8
363.21
364.23
725.33
726.28
786.53
%
m/z

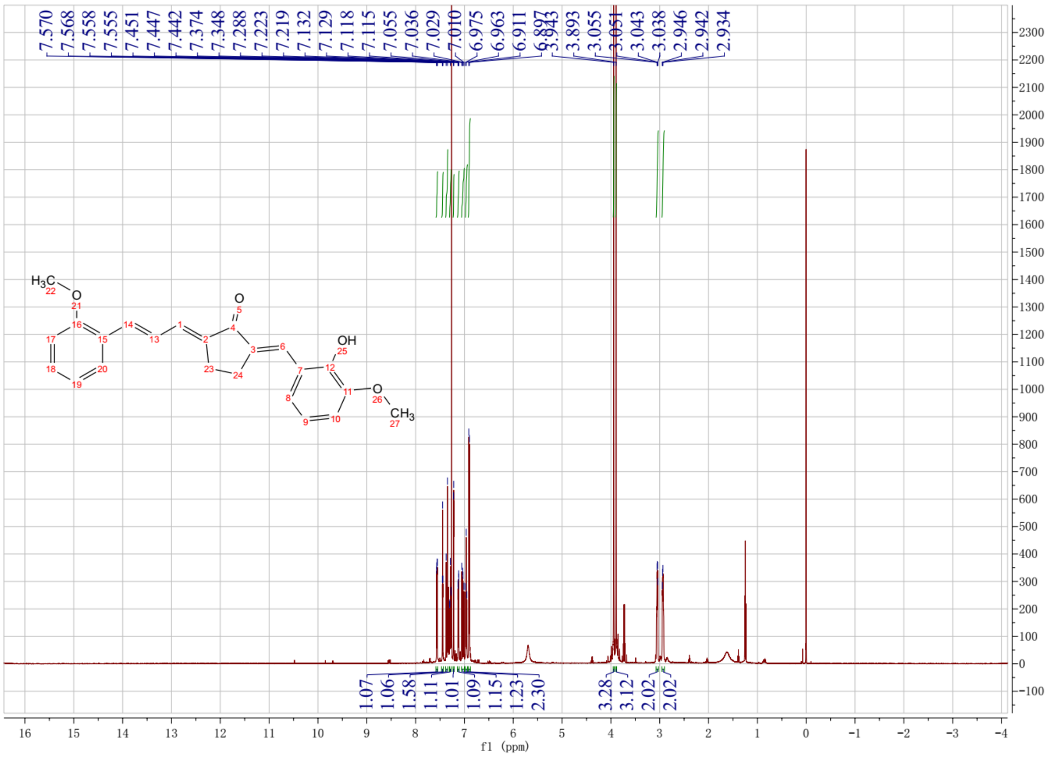

f1 (ppm)

18

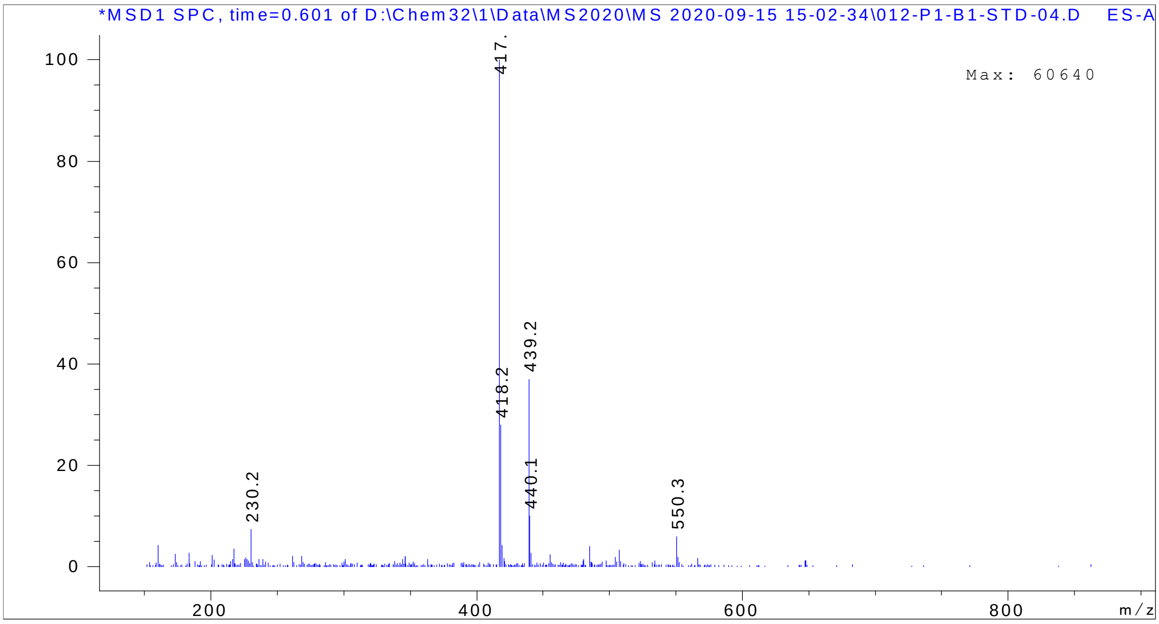
*MSD1 SPC, time=0.601 of D:\Chem32\1\Data\MS2020\MS 2020-09-15 15-02-34\012-P1-B1-STD-04.D ES-A
Max: 60640
417.
439.2
418.2
440.1
230.2
550.3
m/z

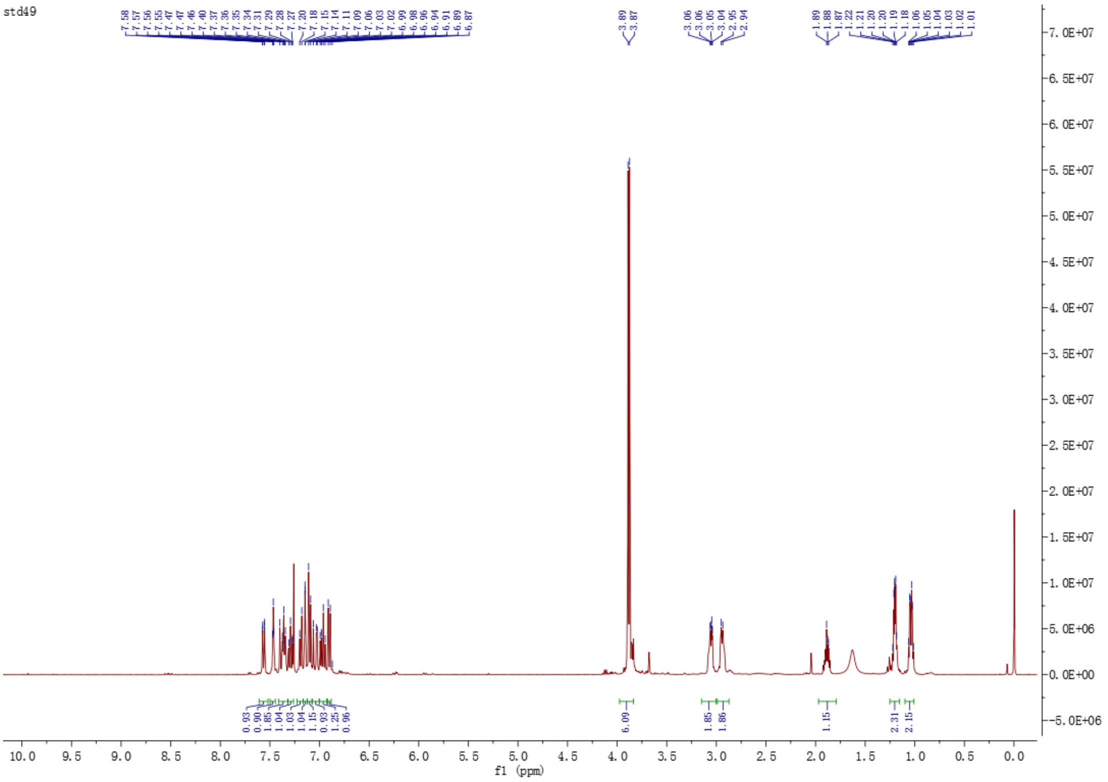
std49
f1 (ppm)

19

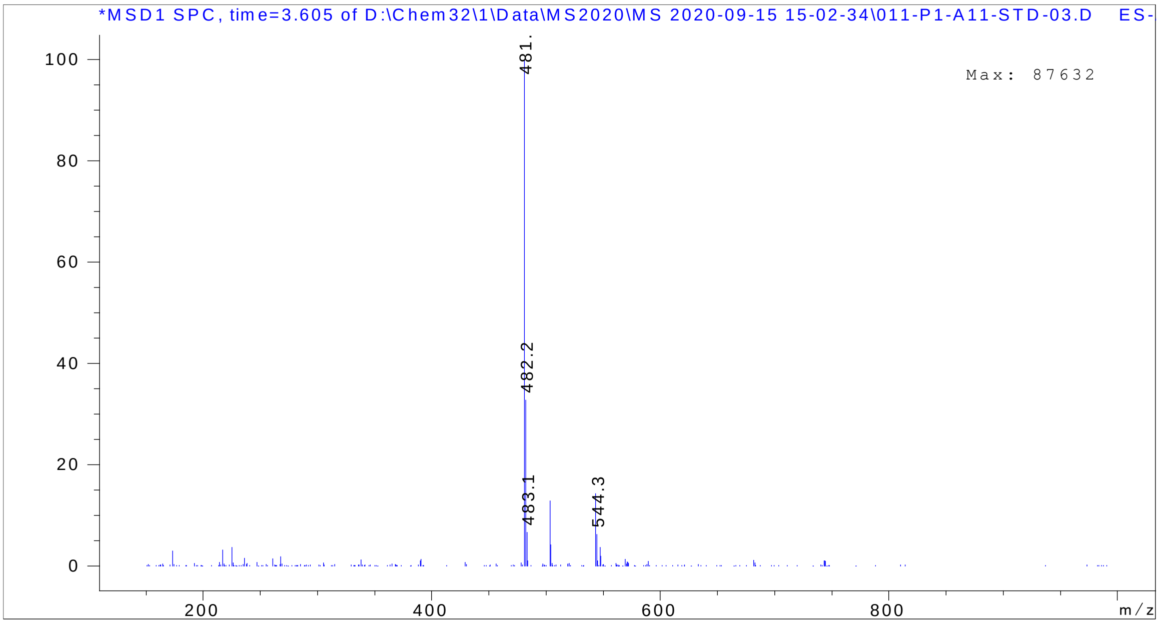
*MSD1 SPC, time=3.605 of D:\Chem32\1\Data\MS2020\MS 2020-09-15 15-02-34\011-P1-A11-STD-03.D ES-
Max: 87632
481.
482.2
483.1
544.3
m/z

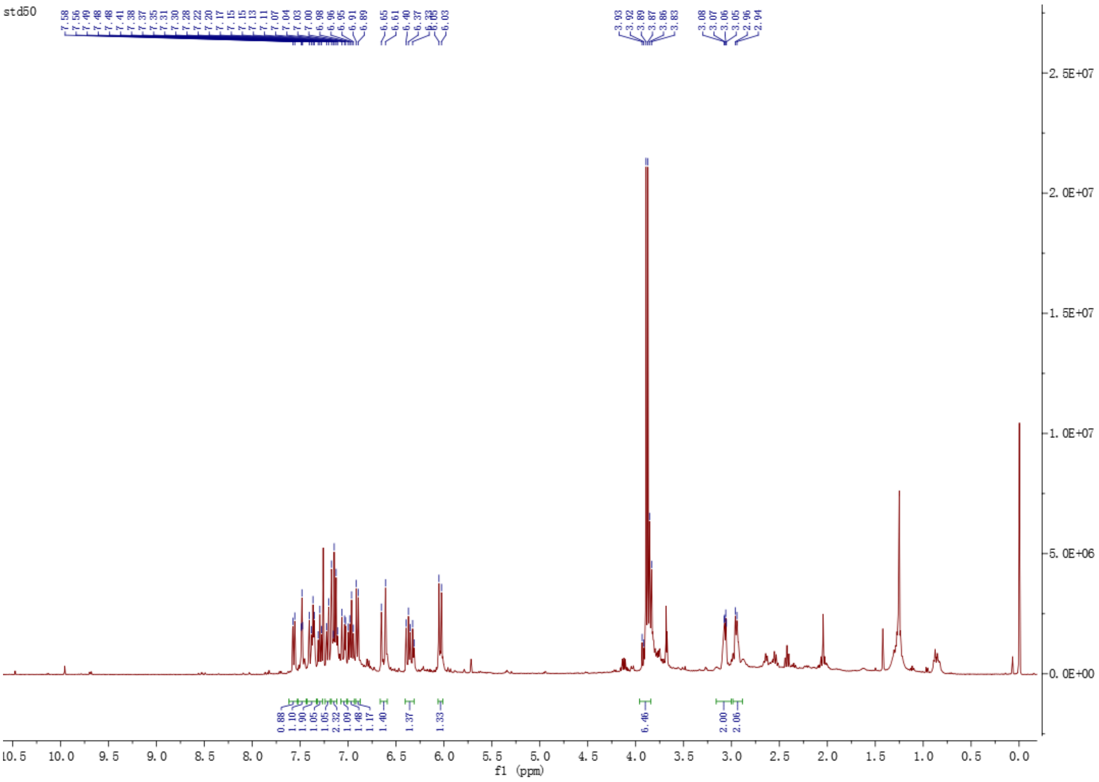
std50
f1 (ppm)

20

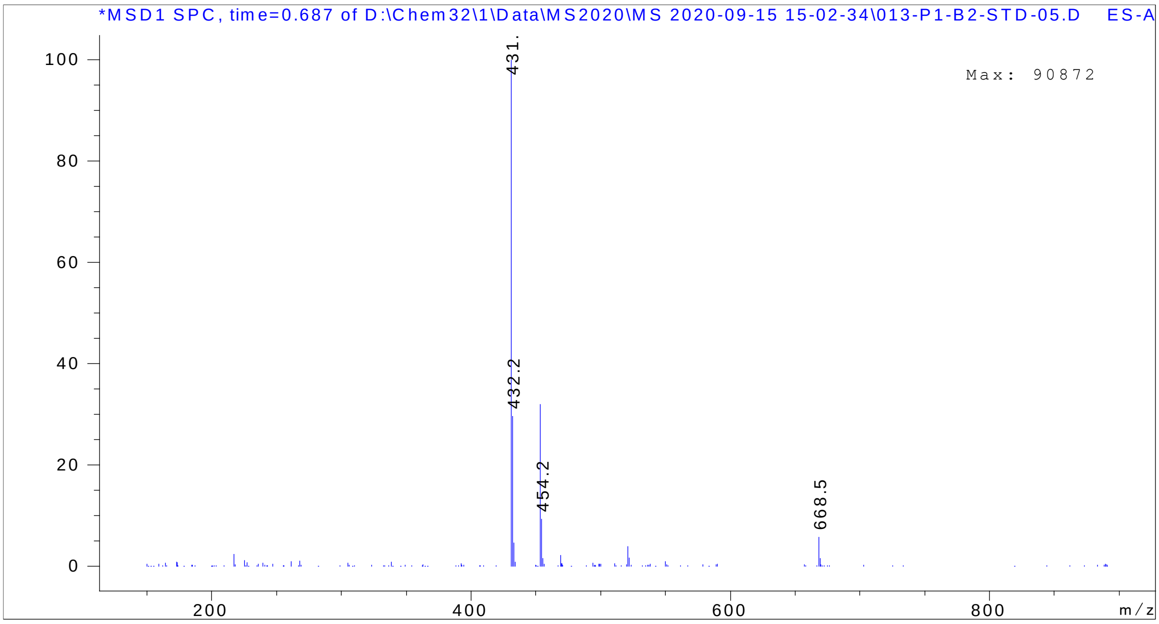
*MSD1 SPC, time=0.687 of D:\Chem32\1\Data\MS2020\MS 2020-09-15 15-02-34\013-P1-B2-STD-05.D ES-A
Max: 90872
431.
432.2
454.2
668.5
m/z

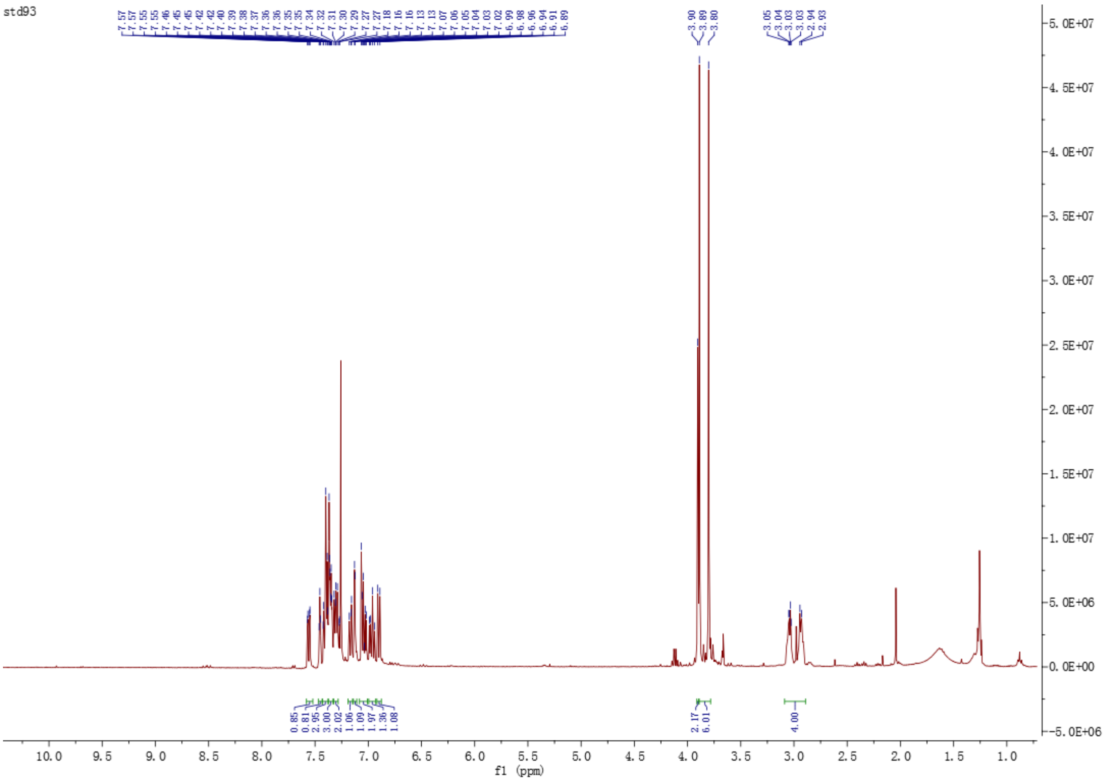
std93
f1 (ppm)
